# Coordinated activation of c-Src and FOXM1 drives tumor cell proliferation and breast cancer progression

**DOI:** 10.1172/JCI162324

**Published:** 2023-04-03

**Authors:** Ipshita Nandi, Harvey W. Smith, Virginie Sanguin-Gendreau, Linjia Ji, Alain Pacis, Vasilios Papavasiliou, Dongmei Zuo, Stella Nam, Sherif S. Attalla, Sung Hoon Kim, Sierra Lusson, Hellen Kuasne, Anne-Marie Fortier, Paul Savage, Constanza Martinez Ramirez, Morag Park, John A. Katzenellenbogen, Benita S. Katzenellenbogen, William J. Muller

**Affiliations:** 1 Rosalind and Morris Goodman Cancer Institute and; 2 Department of Biochemistry, McGill University, Montreal, Quebec, Canada.; 3 Canadian Centre for Computational Genomics, McGill Genome Centre, Montreal, Quebec, Canada.; 4 Department of Chemistry, University of Illinois at Urbana-Champaign, Champaign, Illinois, USA.; 5 Department of Surgery, University of Toronto, Toronto, Ontario, Canada.; 6 Department of Medicine and; 7 Department of Oncology, McGill University, Montreal, Quebec, Canada.; 8 Department of Chemistry and Cancer Center and; 9 Department of Molecular and Integrative Physiology, Cancer Center and Institute for Genomic Biology, University of Illinois and College of Medicine at Urbana-Champaign, Champaign, Illinois, USA.

**Keywords:** Oncology, Therapeutics, Breast cancer, Cell cycle, Mouse models

## Abstract

Activation of the tyrosine kinase c-Src promotes breast cancer progression and poor outcomes, yet the underlying mechanisms are incompletely understood. Here, we have shown that deletion of c-Src in a genetically engineered model mimicking the luminal B molecular subtype of breast cancer abrogated the activity of forkhead box M1 (FOXM1), a master transcriptional regulator of the cell cycle. We determined that c-Src phosphorylated FOXM1 on 2 tyrosine residues to stimulate its nuclear localization and target gene expression. These included key regulators of G_2_/M cell-cycle progression as well as c-Src itself, forming a positive feedback loop that drove proliferation in genetically engineered and patient-derived models of luminal B–like breast cancer. Using genetic approaches and small molecules that destabilize the FOXM1 protein, we found that targeting this mechanism induced G_2_/M cell-cycle arrest and apoptosis, blocked tumor progression, and impaired metastasis. We identified a positive correlation between FOXM1 and c-Src expression in human breast cancer and show that the expression of FOXM1 target genes predicts poor outcomes and associates with the luminal B subtype, which responds poorly to currently approved therapies. These findings revealed a regulatory network centered on c-Src and FOXM1 that is a targetable vulnerability in aggressive luminal breast cancers.

## Graphical abstract

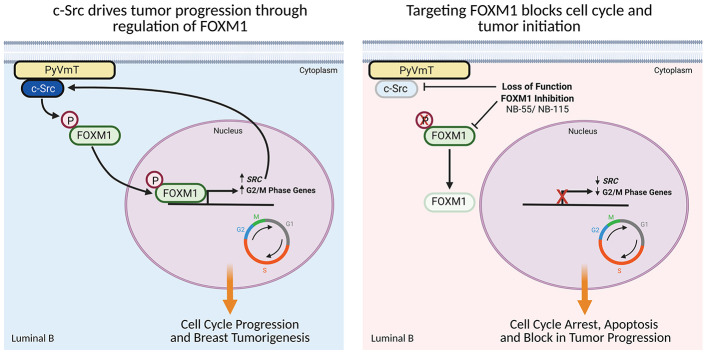

## Introduction

Breast cancer comprises a range of histopathological and molecular subtypes differing in metastatic capacity, response to therapies, and clinical outcomes (1). Transcriptomic profiling has identified at least 5 breast cancer subtypes, including human epidermal growth factor receptor 2–positive (HER2^+^), luminal A, luminal B, normal-like, and basal-like breast cancer (1, 2). Luminal subtypes are the most common and are characterized by gene expression patterns resembling those of luminal mammary epithelial cells and frequent expression of the steroid hormone receptors estrogen receptor α (ERα) and progesterone receptor (PR). The standard of care in these cases includes endocrine therapies targeting ERα and, more recently, inhibitors of cyclin-dependent kinases 4 and 6 (CDK4/-6), particularly in the metastatic setting (3). Luminal B tumors, which constitute 15%–20% of all cases, express lower levels of ERα than do luminal A tumors and exhibit a higher proliferative rate, more aggressive metastatic behavior, and a poor response to endocrine therapy. They present an urgent, unmet clinical need due to a lack of viable therapeutic options, leading to poor outcomes for patients (1, 4).

Preclinical and clinical evidence suggests that overexpression and activation of c-Src, a nonreceptor tyrosine kinase, is associated with breast cancer progression (5–10). c-Src activates mitogenic and proinvasive signaling pathways downstream of receptor tyrosine kinases, integrins, and other receptors (11, 12) including ERα, which is phosphorylated by c-Src on Y537 to promote its “nongenomic” scaffolding function that activates oncogenic signaling (13, 14). Elevated c-Src activity can also mediate resistance to targeted therapies such as anti-HER2 agents and endocrine therapies (15–17). Due partly to a lack of specific c-Src inhibitors suitable for clinical use, identifying the effectors and downstream processes through which c-Src drives breast cancer progression may be a more promising therapeutic strategy than directly targeting c-Src itself. This approach relies on a strong mechanistic understanding of c-Src function in specific breast cancer subtypes. However, previous studies targeting *Src* in genetically engineered mouse models (GEMMs) (8, 9) had technical limitations, including impaired mammary gland development in germline *Src*-knockout mice (18) and escape from Cre-LoxP–mediated conditional gene targeting in mammary epithelial cells (9), that affected data interpretation and hindered further advances.

Here, we describe an approach for efficient genetic targeting of c-Src in a GEMM expressing the polyomavirus middle-T antigen (PyVmT), in which premalignant mammary lesions progress to highly metastatic adenocarcinoma recapitulating pathological and molecular features of luminal B tumors (19–21). By combining this GEMM with organoid and patient-derived models, we showed that c-Src and forkhead box protein M1 (FOXM1), a key transcriptional regulator of the cell cycle (22), formed a positive feedback loop driving the cell-cycle progression that is required for efficient progression of luminal B–like (ER^+^Ki67^hi^) (23, 24) breast cancer beyond an early hyperplastic stage. We present evidence of closely coordinated expression and activation of c-Src and FOXM1 in human breast cancer and demonstrate that targeting c-Src/FOXM1 activity, including through small molecules that trigger FOXM1 degradation, arrested cell-cycle progression, induced apoptosis, and impaired tumor progression and metastasis in multiple preclinical models. These findings uncover a crucial mechanism of c-Src–dependent tumor progression that presents an important therapeutic target in luminal B–like breast cancers.

## Results

### c-Src ablation impairs tumor progression and metastasis in a model of aggressive luminal breast cancer.

**Figure 1:**
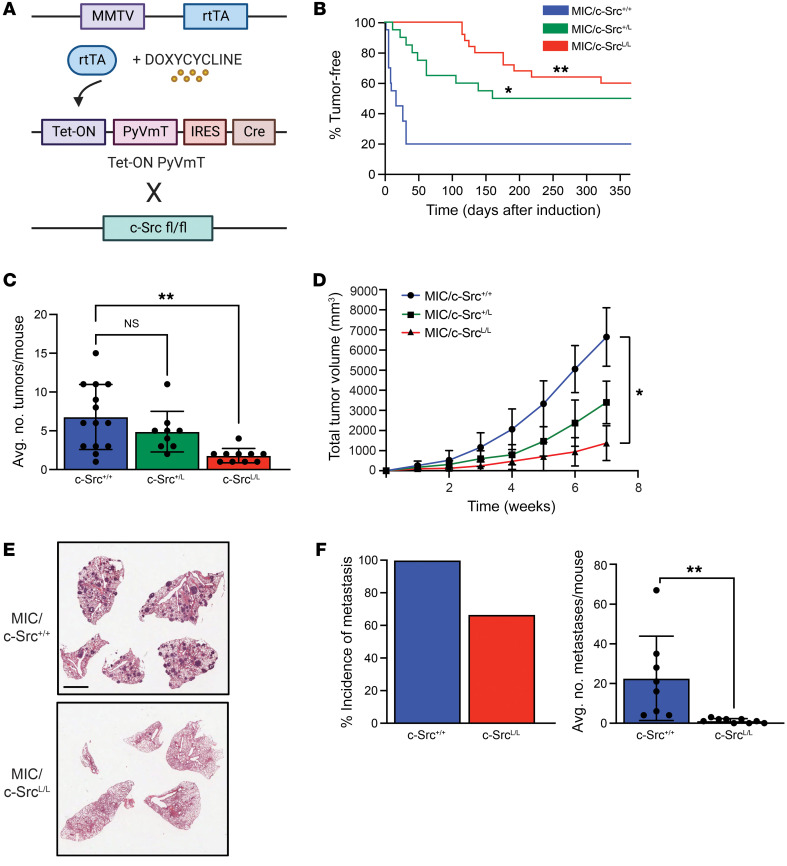
c-Src ablation impairs tumor progression and metastasis in a preclinical model of luminal B breast cancer. (**A**) Schematic of the genetically engineered mouse model. (**B**) Kaplan-Meier analysis of mammary tumor onset in mice with WT *Src* alleles (MIC/c-Src^+/+^, *n* = 20) and heterozygous (MIC/c-Src^+/L^, *n* = 20) or homozygous (MIC/c-Src^L/L^, *n* = 25) conditional *Src* alleles. *P < 0.05 and **P < 0.01, by log-rank test. (**C** and **D**) Average (Avg.) number of mammary tumors per mouse (**C**) and total tumor volume (**D**) for mice as in **A**. *P < 0.05 and **P < 0.01, by 1-way ANOVA with Dunnett’s post hoc test. (**E**) Representative images of H&E-stained lung sections at the endpoint. Scale bar: 5 mm. (**F**) Incidence and average burden of lung metastasis. **P < 0.01, by unpaired, 2-tailed Student’s *t* test.

We established a GEMM combining conditional *Src* alleles (c-Src^L/L^) (9) with the TetO-PyV mT-IRES-Cre (MIC) (25) and mouse mammary tumor virus long terminal repeat–reverse tetracycline transactivator (MMTV-rtTA) transgenes (26) (Figure 1A). In this model, PyVmT and Cre recombinase are expressed from a bi-cistronic transcript in a mammary epithelium–specific, doxycycline-inducible manner. This approach allows temporally controlled deletion of targeted alleles specifically in cells expressing the PyVmT oncogene, with no possibility of escape from Cre-mediated recombination (25). Ablation of one (c-Src^+/L^) or both (c-Src^L/L^) *Src* alleles extended the time to tumor onset and reduced tumor penetrance (Figure 1B). Tumors that eventually developed in mice with conditional *Src* alleles were more focal and slow-growing than were WT (MIC/c-Src^+/+^) controls (Figure 1, C and D). In all cases, we observed the most severe phenotypes in MIC/c-Src^L/L^ tumors. While MIC/c-Src^+/+^ tumors were highly metastatic to the lungs (25), c-Src ablation significantly reduced the penetrance and burden (number of metastases per mouse) of lung metastasis (Figure 1, E and F). In vitro genetic ablation of *Src* severely impaired PyVmT cell migration and invasion, as well as lung colonization in vivo, arguing that these effects on metastasis cannot be fully ascribed to reduced tumor burden (Supplemental Figure 1; supplemental material available online with this article; https://doi.org/10.1172/JCI162324DS1).

**Figure 2:**
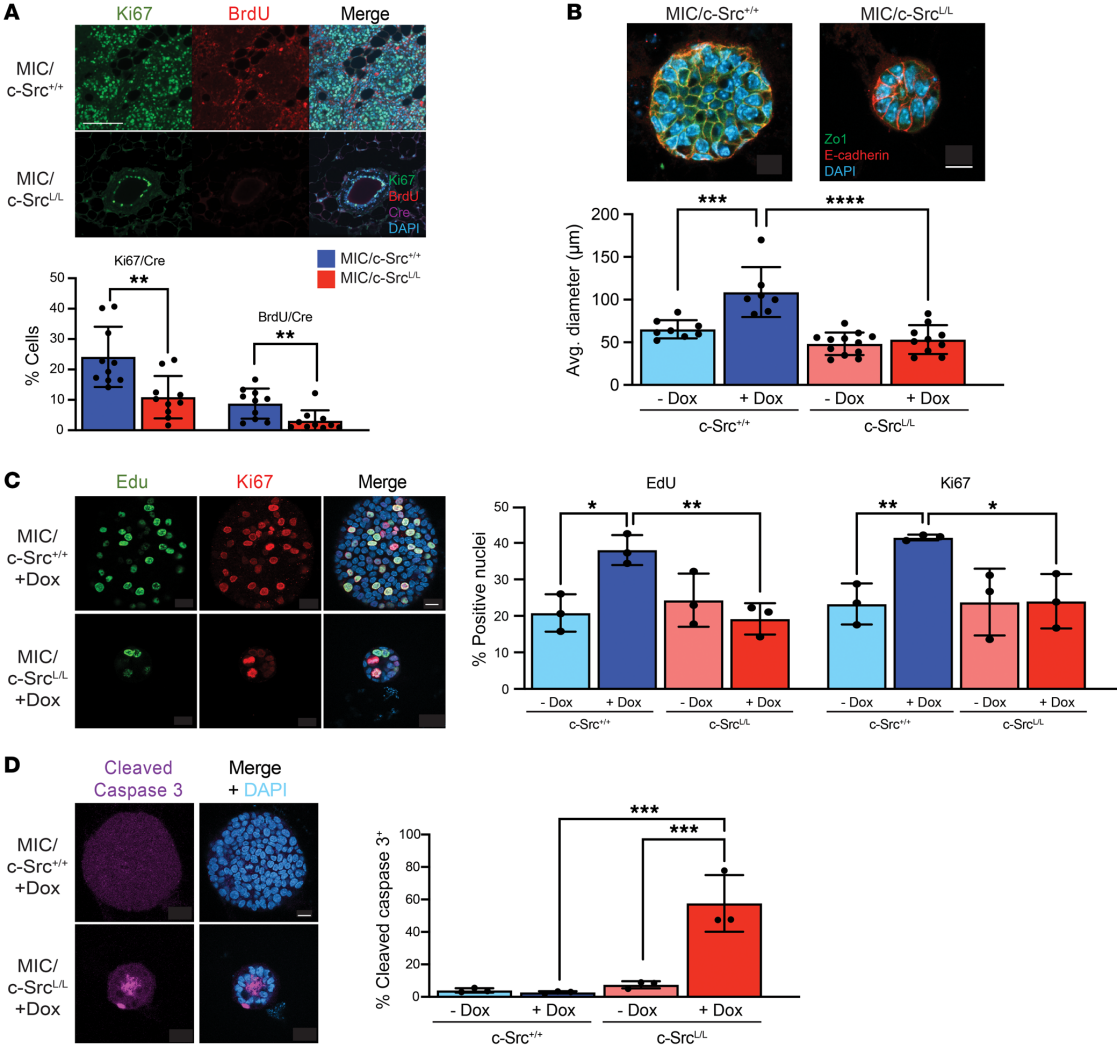
c-Src ablation impairs proliferation and increases apoptosis in an organoid model of early mammary tumor progression. (**A**) Mammary glands from c-Src^+/+^ and c-Src^L/L^ MIC mice were immunostained with the indicated antibodies and DAPI. Representative images and quantification of Ki67^+^ and BrdU^+^ nuclei, normalized to Cre. *n* = 10 mice per group (minimum of 10,000 total nuclei per sample). Scale bar: 100 μm. **P < 0.01, by unpaired, 2-tailed Student’s *t* test. (**B**) Organotypic cultures were immunostained with the indicated antibodies and DAPI, and the organoid diameter was measured. Scale bar: 10 μm. ***P < 0.001 and ****P < 0.0001, by 1-way ANOVA with Tukey’s post hoc test. (**C**) Organoids were immunostained with the indicated antibodies (left panel), and staining was quantified and normalized to total nuclei (DAPI) (right panel). Scale bar: 10 μm. *n* = 3 independent mice per genotype (minimum of 20 total nuclei analyzed per mouse). *P < 0.05 and **P < 0.01, by 1-way ANOVA with Tukey’s post hoc test. (**D**) Organoids were immunostained to detect cleaved caspase 3 (left panel). Staining was quantified and normalized to the total number of cells, as determined by DAPI staining (right panel). *n* = 3 independent mice per genotype (minimum of 20 total nuclei analyzed per mouse). Scale bar: 10 μm. ***P < 0.001, by 1-way ANOVA with Tukey’s post hoc test. Dox, doxycycline.

Examination of premalignant mammary glands confirmed that MIC/c-Src^L/L^ mammary epithelia lacked c-Src protein and exhibited minimal evidence of transformation, whereas MIC/c-Src^+/+^ mammary glands exhibited extensive hyperplasia and premalignant lesions (Supplemental Figure 2, A and B). This correlated with significantly reduced proliferation, impaired cell-cycle progression, and increased apoptosis in c-Src–deficient mammary epithelial cells (Figure 2A and Supplemental Figure 2C). Because Src family kinase–dependent (SFK-dependent) tyrosine phosphorylation of the PyV mT antigen itself is required to recruit signaling effectors that activate Ras/MAPK, PI-3K/Akt, and other pathways to drive tumorigenesis (27–29), we investigated the activation status of these pathways in c-Src–deficient versus –proficient mammary epithelium. We observed no differences in Erk or Akt phosphorylation, indicating that PyV mT oncogenic signaling was comparably activated in both contexts (Supplemental Figure 2A). Conversely, loss of c-Src affected the phosphorylation of the known SFK target sites focal adhesion kinase (FAK) (Y576) and STAT3 (Y705). This is consistent with previous findings that other SFKs such as Fyn and c-Yes can maintain canonical PyV mT signaling in the absence of c-Src (9, 10), implying that distinct pathways regulated specifically by c-Src are important for tumor progression.

### Loss of c-Src function blocks early breast cancer progression.

To examine tumor-initiating events further and provide a tractable system for molecular analysis, we established 3D organotypic cultures (30) from MIC/c-Src^+/+^ and MIC/c-Src^L/L^ mammary epithelia (Supplemental Figure 2D). Following induction of the MIC transgene, WT organoids formed solid structures with a collapsed lumen and epithelial stratification, whereas c-Src–deficient organoids were smaller with an intact lumen (Figure 2B) and retained membrane localization of the adherens junction marker E-cadherin and the tight junction marker ZO-1, both of which were mislocalized in MIC/c-Src^+/+^ organoids (Figure 2B and Supplemental Figure 2E). c-Src ablation also reduced proliferation and elevated apoptosis in organoids, with apoptotic cells accumulating within the lumen (Figure 2, C and D).

**Figure 3:**
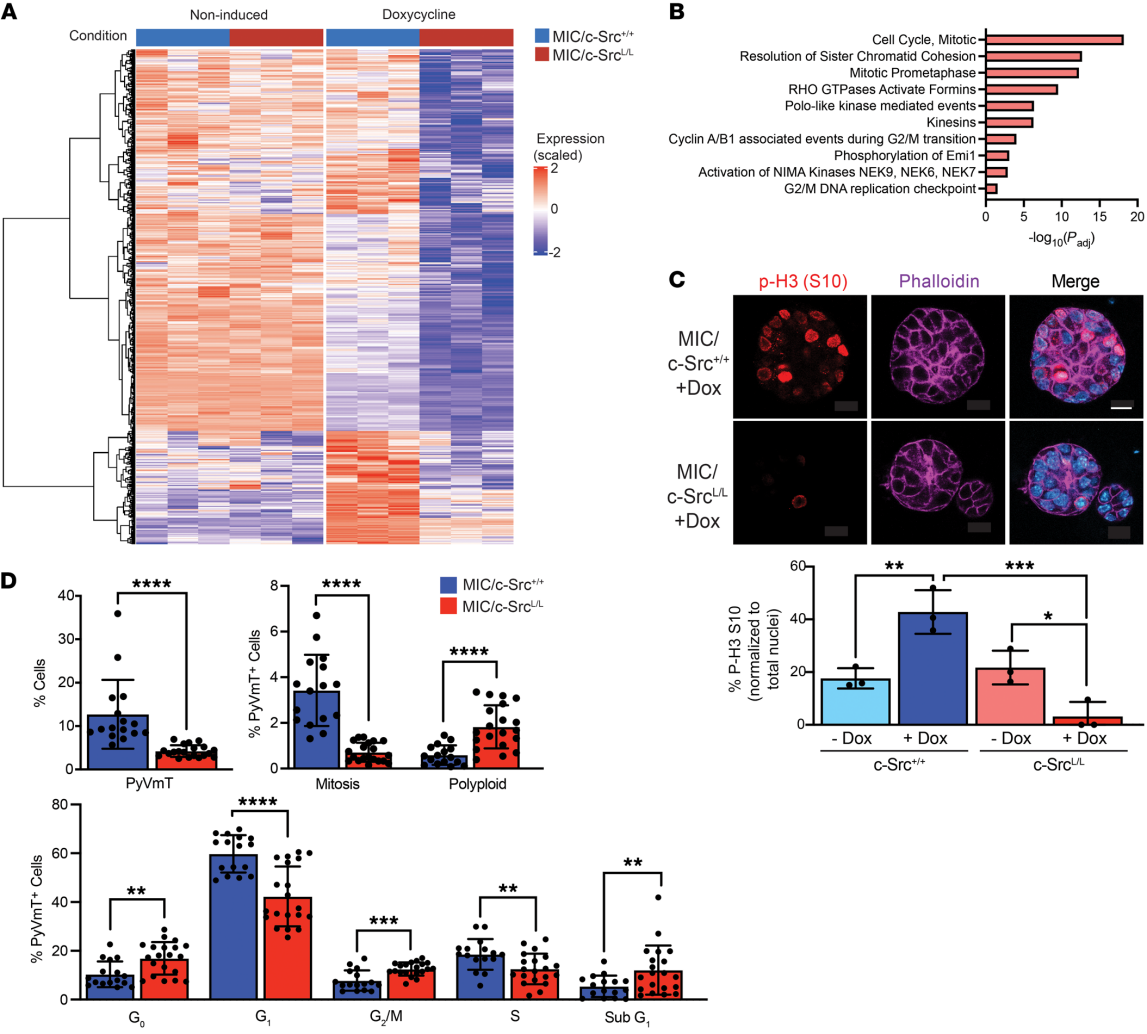
Deletion of c-Src inhibits mitosis and causes G_2_/M cell-cycle arrest. (**A**) Unsupervised hierarchical clustering analysis of genes differentially upregulated (red) and downregulated (blue) in doxycycline-induced and noninduced MIC/c-Src^L/L^ mammary organoids compared with MIC/c-Src^+/+^ controls (*n* = 3 per genotype). (**B**) Reactome pathway analysis of genes downregulated in c-Src^L/L^ organoids. Padj, adjusted *P* value. (**C**) Top panel: Organoids were stained with an antibody specific for p-H3 (S10), phalloidin (F-actin), and DAPI (nuclei). Scale bar: 10 μm. Bottom panel: p-H3 (S10) staining was quantified and normalized to total nuclei (DAPI). *n* = 3 mice per genotype (minimum of 20 total nuclei analyzed per mouse). *P < 0.05, **P < 0.01, and ***P < 0.001, by 1-way ANOVA with Tukey’s post hoc test. (**D**) Flow cytometric analysis of MIC/c-Src^+/+^ or c-Src^L/L^ mammary glands induced for 4 weeks with doxycycline (*n* = 20 per genotype; minimum of 250,000 cells per sample). **P < 0.01, ***P < 0.001, and ****P < 0.0001, by unpaired, 2-tailed Student’s *t* test.

To gain mechanistic insight into the phenotypes associated with c-Src deficiency, we used RNA-Seq to identify 248 transcripts upregulated and 175 transcripts downregulated in MIC/c-Src^L/L^ organoids compared with MIC/c-Src^+/+^ controls following doxycycline administration (Figure 3A). The downregulated genes were dominated by cell-cycle pathways, especially those specific to mitosis (Figure 3B and Supplemental Figure 3A). Accordingly, c-Src deletion significantly reduced histone H3 phosphorylation on serine 10 [p-H3 (S10)], a marker of mitosis (Figure 3C). Flow cytometric analysis (Supplemental Figure 3B) of premalignant mammary epithelia revealed that c-Src ablation increased quiescence (G_0_), reduced entry into G_1_ and S phases, and markedly increased the percentage of cells in G_2_ while decreasing the percentage of cells in mitosis (Figure 3D), indicating that loss of c-Src blocked cell-cycle progression in G_2_ or at the G_2_/M transition. We also identified increases in polyploid cells and sub-G_1_ cells (indicating cell death) in c-Src–deficient mammary glands. Collectively, these observations argue that c-Src ablation can induce quiescence, cell-cycle arrest, abnormal cell division leading to polyploidy, and apoptosis in premalignant mammary epithelial cells. Using genetically engineered cell lines as described above, we show that acute c-Src loss caused similar cell-cycle phenotypes in fully transformed breast cancer cells (Supplemental Figure 3C).

**Figure 4:**
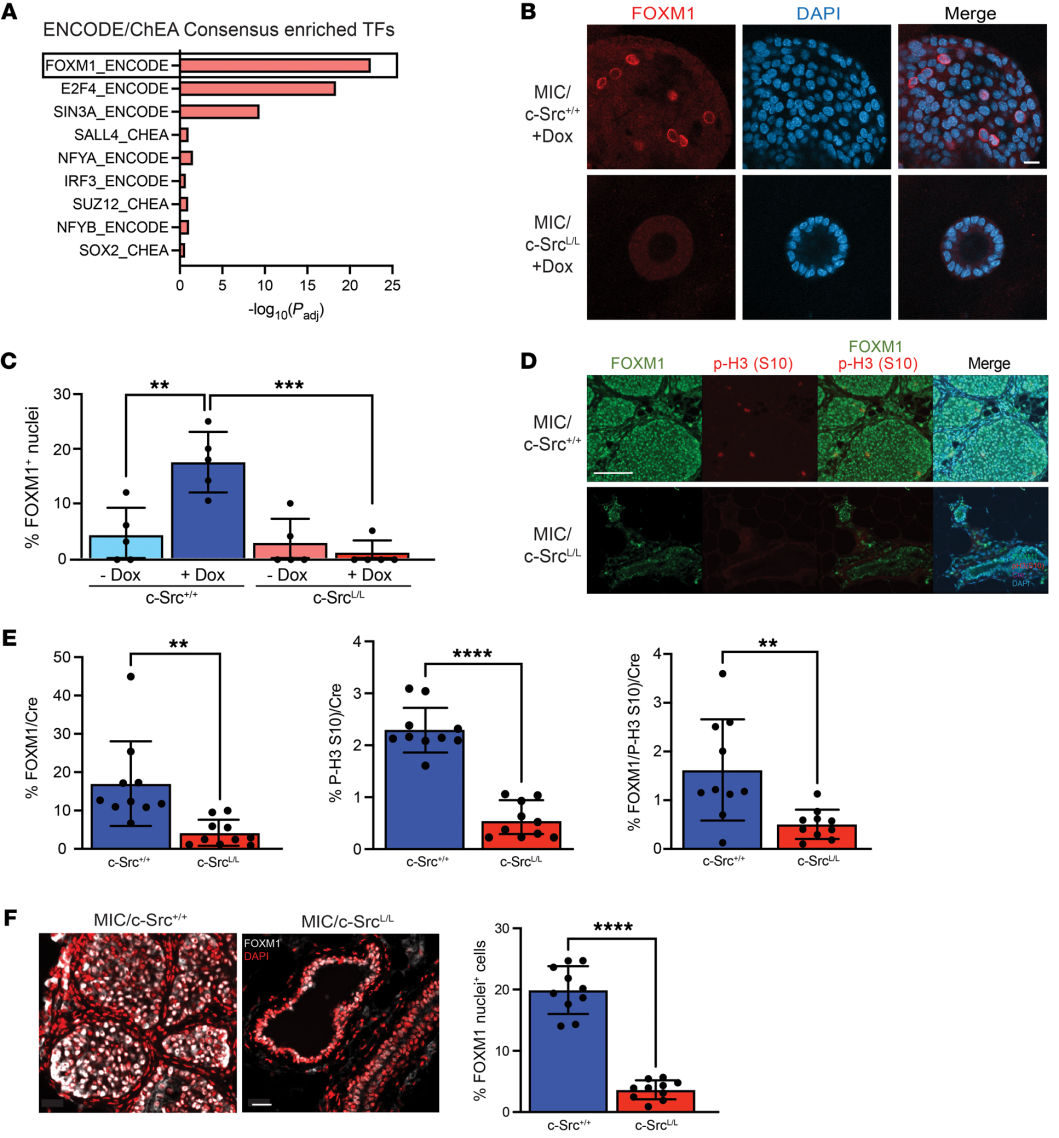
c-Src deletion reduces FOXM1 expression and activity in vivo. (**A**) Transcription factor signature analysis of genes downregulated in c-Src^L/L^ organoids compared with controls. (**B** and **C**) MIC organoids were immunostained to detect FOXM1, and nuclei were counterstained with DAPI. (**B**) Images are representative of 5 independent mice per genotype. Scale bar: 100 μm. (**C**) Staining was quantified, and FOXM1^+^ nuclei were normalized to total nuclei (minimum of 20 total nuclei per sample). **P < 0.01 and ***P < 0.001, by 1-way ANOVA with Tukey’s post hoc test. (**D** and **E**) MIC mammary glands were immunostained with the indicated antibodies and DAPI. Scale bar: 100 μm. (**D**) Images are representative of 10 independent mice of each genotype. (**E**) Staining was quantified and normalized to total cell numbers (DAPI). Minimum of 10,000 cells per sample. **P < 0.01 and ****P < 0.0001, by unpaired, 2-tailed Student’s *t* test. (**F**) MIC mammary gland sections were immunostained to detect FOXM1, and nuclei were counterstained with DAPI. Left panel: Images are representative of 10 independent mice of each genotype. Scale bar: 20 μm. Right panel: Nuclear staining was quantified by colocalization of FOXM1 immunofluorescence with DAPI. Minimum of 10,000 cells per sample. ****P < 0.0001, by unpaired, 2-tailed Student’s *t* test.

Further bioinformatics analysis of the transcriptomic data revealed that FOXM1, a critical regulator of G_2_/M-phase cell-cycle progression (22), was the transcription factor most substantially affected by c-Src deficiency (Figure 4A). Overall, 37.7% (66 of 175) of the genes downregulated in c-Src–deficient organoids were known FOXM1 targets (Supplemental Figure 4A), including *Foxm1* itself (31). Through binding motif analysis, cross-referencing with ChIP-Seq data from luminal breast cancer cells, and unsupervised hierarchal clustering, we confirmed that a FOXM1 target gene signature including many key regulators of the G_2_/M phase and mitotic progression was notably repressed in organoids lacking c-Src (Supplemental Figure 4B). c-Src ablation in organoids and in vivo led to downregulation of FOXM1 at the mRNA and protein levels (Supplemental Figure 4, C–E). Compared with controls, in which we detected FOXM1 in the nucleus and the cytoplasm, c-Src–deficient MIC organoids and mammary epithelia lacked significant nuclear localization of FOXM1 (Figure 4, B–F), correlating with reduced p-H3 (S10) (Figure 4, D and E, and Supplemental Figure 4, C and D) and loss of its colocalization with FOXM1 (Figure 4E). Together, these data argue that signals downstream of c-Src activation were required for nuclear localization and expression of FOXM1 in mammary epithelial premalignant lesions, resulting in loss of expression of FOXM1 target genes and impaired entry into mitosis in the absence of c-Src.

### c-Src phosphorylates FOXM1 to promote transcriptional activation of cell-cycle progression.

**Figure 5:**
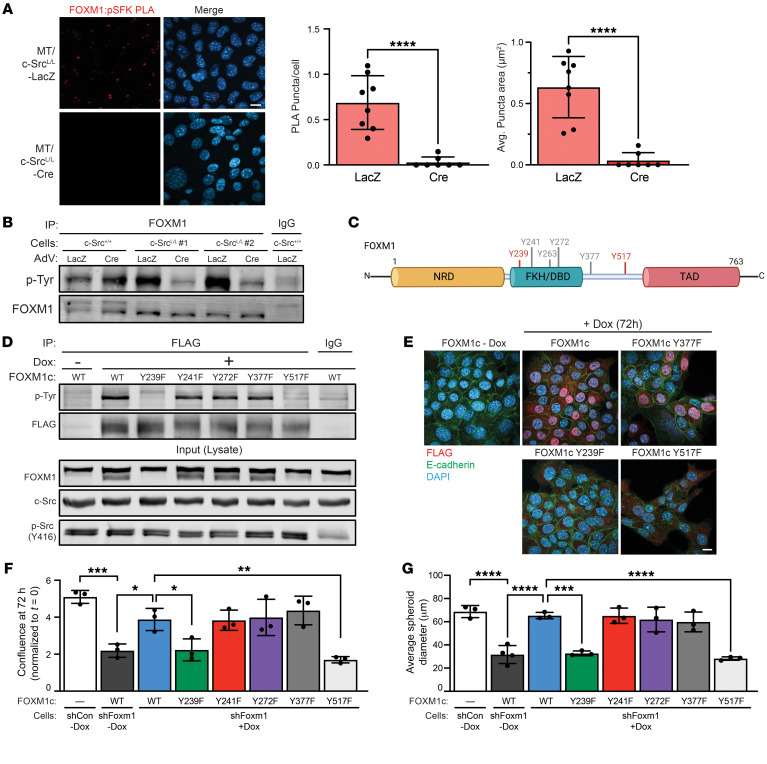
FOXM1 interacts with and is phosphorylated by c-Src. (**A**) PLA to detect interactions of p-SFK (Y416) and FOXM1 in PyVmT murine breast cancer cells with WT (c-Src^+/+^) or conditional (c-Src^L/L^) *Src* alleles, 96 hours after adenoviral (AdV) delivery of Cre or LacZ. Left panel: Representative images. Scale bar: 10 μm. Right panel: The number and size of PLA puncta were quantified in two PyVmT/c-Src^L/L^ cell lines (minimum 30 cells analyzed per sample). ****P < 0.0001, by unpaired, 2-tailed Student’s *t* test. (**B**) FOXM1 immunoprecipitates from cells as in **A** were immunoblotted with the indicated antibodies. (**C**) Schematic illustration of tyrosine residues conserved between murine and human FOXM1. c-Src–dependent tyrosine phosphorylation sites are indicated in red. NRD, N-terminal repressor domain; FKH/DBD, forkhead winged helix DNA-binding domain; TAD, transactivation domain. (**D**) WT and Y–F mutant FOXM1 expression was induced in shFOXM1 cells by doxycycline treatment. FLAG-FOXM1 immunoprecipitates and total cell lysates were immunoblotted with the indicated antibodies. Immunoprecipitation with nonspecific IgG and FLAG immunoprecipitation from untreated cells were used as controls. (**E**) MT/c-Src^+/+^ shFOXM1 cells stably transduced with FOXM1 constructs were treated with doxycycline and immunostained as indicated. Scale bar: 20 μm. (**F**) Proliferation was assayed in PyVmT cells expressing the indicated shRNAs and FOXM1 constructs using an imaging-based assay to measure cell confluence. *n* = 3 cell lines. *P < 0.05, **P < 0.01, and ***P < 0.001, by 1-way ANOVA with Tukey’s post hoc test. (**G**) Cells as in **F** were grown in 3D culture conditions as tumor spheroids, and spheroid diameters were measured by microscopy. *n* = 3 cell lines. ***P < 0.001 and ****P < 0.0001, by 1-way ANOVA with Tukey’s post hoc test.

FOXM1 is extensively posttranslationally modified (32–36), including by phosphorylation mainly on serine (88.1%) and threonine (11.4%) residues (37, 38). However, despite its low abundance, tyrosine phosphorylation is also important in mitotic progression (39), and c-Src has long been associated with aberrant cell-cycle regulation in cancer (40, 41), although the underlying mechanisms remain to be elucidated. Using a proximity ligation assay (PLA), we detected interactions involving endogenous FOXM1 and activated SFKs phosphorylated on Y416 [p-SFK (Y416)] in c-Src–proficient tumor cells and premalignant mammary epithelial cells (Figure 5A and Supplemental Figure 5A). PLA signals were largely absent in cells and tissue lacking c-Src, with residual signals in c-Src–null mammary glands likely occurring mainly in stromal cells (which retain c-Src) and/or involving other SFKs, which are expressed in epithelial and stromal cells (8, 9). Providing further evidence for an interaction, we also detected coimmunoprecipitation of FOXM1 and c-Src in PyVmT cell lysates (Supplemental Figure 5B).

We detected robust tyrosine phosphorylation of FOXM1 immunoprecipitates, which was markedly reduced by c-Src ablation (Figure 5B) and by stable shRNA-mediated silencing of FOXM1, commensurate with loss of FOXM1 protein expression (Supplemental Figure 5, B–D). c-Src also phosphorylated FOXM1 on tyrosine in vitro (Supplemental Figure 5E). To examine this further, we mutated each of the 6 FOXM1 tyrosine residues conserved between humans and mice (42) to phenylalanine and induced the expression of each epitope-tagged tyrosine-to-phenylalanine (Y–F) mutant in cells with stable silencing of endogenous FOXM1, using WT FOXM1 as a control (Supplemental Figure 5F) (43). Although all constructs were expressed comparably (Supplemental Figure 5G), mutation of Y239 (in the forkhead winged helix/DNA-binding domain) or Y517 (in the cysteine-rich domain) ablated tyrosine phosphorylation of FOXM1 in PyVmT cells (Figure 5, C and D). In cells lacking endogenous FOXM1, WT FOXM1 localized to the cytoplasm and nucleus, rescued proliferation, restored the expression of genes activated by FOXM1, and reduced the expression of *Atf3*, a gene suppressed by FOXM1 (44, 45) (Figure 5, E and F, and Supplemental Figure 6, A–C). While silencing endogenous FOXM1 impaired cell-cycle progression and tumor spheroid size in 3D conditions mimicking the organoid assays, these phenotypes were also rescued by expression of WT FOXM1 (Figure 5G and Supplemental Figure 6D). In contrast, the Y239F and Y517F mutants were largely excluded from the nucleus and did not rescue phenotypes associated with FOXM1 deficiency under 2D or 3D conditions (Figure 5, E and F, and Supplemental Figure 6, A–D). In all assays, the Y–F mutants retaining tyrosine phosphorylation (e.g., FOXM1 Y377F) rescued phenotypes of FOXM1-deficient cells to control levels in a manner indistinguishable from that of WT FOXM1. We also observed that WT FOXM1 or mutants retaining tyrosine phosphorylation, but not Y239F or Y517F, stimulated proliferation in cells transduced with nontargeting control shRNA (Supplemental Figure 6E), although the effect of increased FOXM1 expression beyond endogenous levels was minor. Together, these data indicate that c-Src stimulated cell-cycle progression and proliferation by interacting with FOXM1 and phosphorylating it on 2 tyrosine residues, promoting its nuclear translocation and activity.

### Targeting FOXM1 blocks proliferation and progression in models of luminal B–like breast cancer.

**Figure 6:**
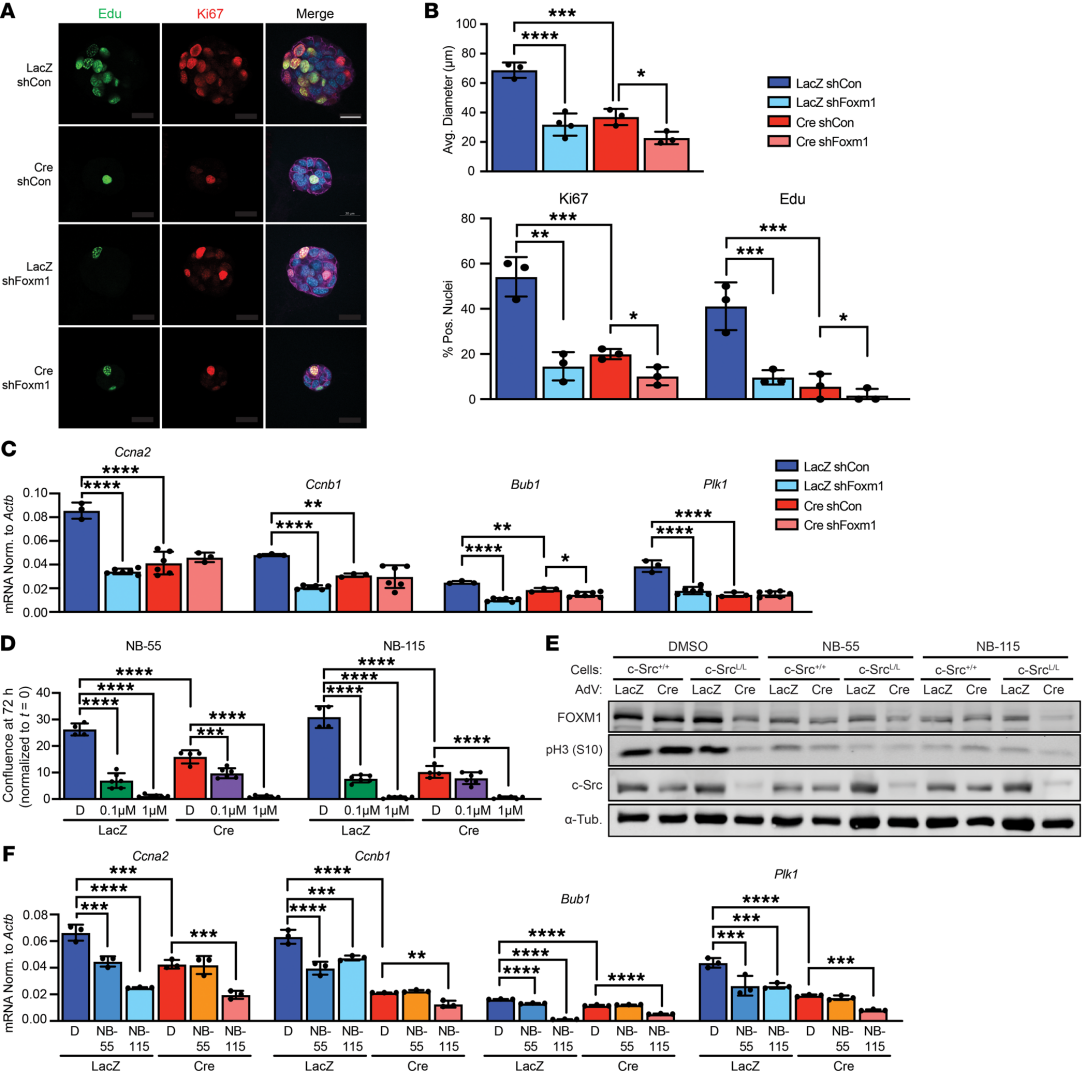
FOXM1 inhibition blocks the proliferation of luminal B breast cancer cells. (**A** and **B**) MT/c-Src^L/L^ cells were grown as 3D spheroids and immunostained to detect Ki67 expression and EdU incorporation. (**A**) Images are representative of 2 cell lines. Scale bar: 20 μm. (**B**) Quantification of spheroid size and Ki67 and EdU staining, normalized to total nuclei. Pos., positive. *P < 0.05, **P < 0.01, ***P < 0.001, and ****P < 0.0001, by 1-way ANOVA with Tukey’s post hoc test. (**C**) qRT-PCR analysis of FOXM1 target gene expression in MT/c-Src^L/L^ cells transduced with the indicated shRNAs and adenoviruses. FOXM1 target expression was normalized (Norm.) to that of *Actb*. Data show the mean of experiments performed in 2 different cell lines in triplicate. *P < 0.05, **P < 0.01, and ****P < 0.0001, by 1-way ANOVA with Tukey’s post hoc test. (**D**) Cells, as in **C**, were treated with FOXM1 inhibitors (NB-55 and NB-155) at the indicated concentrations or with DMSO, and proliferation was assayed using an imaging-based assay. *n* = 2 cell lines in triplicate. ***P < 0.001 and ****P < 0.0001, by 1-way ANOVA with Tukey’s post hoc test. (**E**) Lysates of cells, as in **D**, were immunoblotted with the indicated antibodies. FOXM1 inhibitors were used at 1 μM. α-Tub., α-Tubulin. (**F**) qRT-PCR analysis of FOXM1 target gene expression in cells as in **D** and **E** treated with DMSO (**D**) or FOXM1 inhibitors (NB-55 and NB-115) at 1 μM. Data indicate the mean of experiments performed in 2 different cell lines in triplicate. **P < 0.01, ***P < 0.001, and ****P < 0.0001, by 1-way ANOVA with Tukey’s post hoc test.

FOXM1 overexpression indicates a poor prognosis in many cancers (46) and promotes growth, invasion, metastasis, and resistance to endocrine therapy in breast cancer models (44, 47). As with stable FOXM1 silencing, ablation of c-Src impaired proliferation in PyVmT cells and blocked cell-cycle progression and growth in 3D tumor spheroids (Figure 6, A and B, and Supplemental Figure 7A). To confirm the requirement for SFK activity in these cells, we treated them with the SFK inhibitor (SFKi) dasatinib, the more SFK-specific eCF506 (48), and KB-SRC-4, which exhibits a degree of selectivity for c-Src over other SFKs (49). All 3 SFKi inhibited the growth of PyV mT cells to a similar extent (Supplemental Figure 7B). Since SFKi may affect PyVmT tyrosine phosphorylation (9), we treated the human luminal breast cancer cell line MCF7 and observed a similar effect on proliferation, as well as reduced FOXM1 expression, when SFKs were inhibited (Supplemental Figure 7B). Genetic ablation of c-Src in PyVmT cells reduced the expression of genes activated by FOXM1 and increased the expression of *Atf3*. Combining c-Src ablation with FOXM1 silencing had only a minor additional effect on the proliferation and expression of some targets (Figure 6, B and C, and Supplemental Figure 7C). We obtained similar results using NB-55 and NB-115, compounds that bind FOXM1 with high specificity and nanomolar affinity, enhancing the susceptibility of FOXM1 to proteolysis and triggering proteasomal degradation (50). Similar to observations from myeloma, breast, and ovarian cancer models (47, 51–53), both drugs caused dose-dependent suppression of proliferation and FOXM1 expression, while altering gene expression in a manner consistent with FOXM1 inhibition (Figure 6, D–F, and Supplemental Figure 7, D and E). As with FOXM1 silencing, the response of c-Src–deficient cells to FOXM1 inhibition was markedly weaker, arguing that FOXM1 activity was largely compromised by prior loss of c-Src.

**Figure 7:**
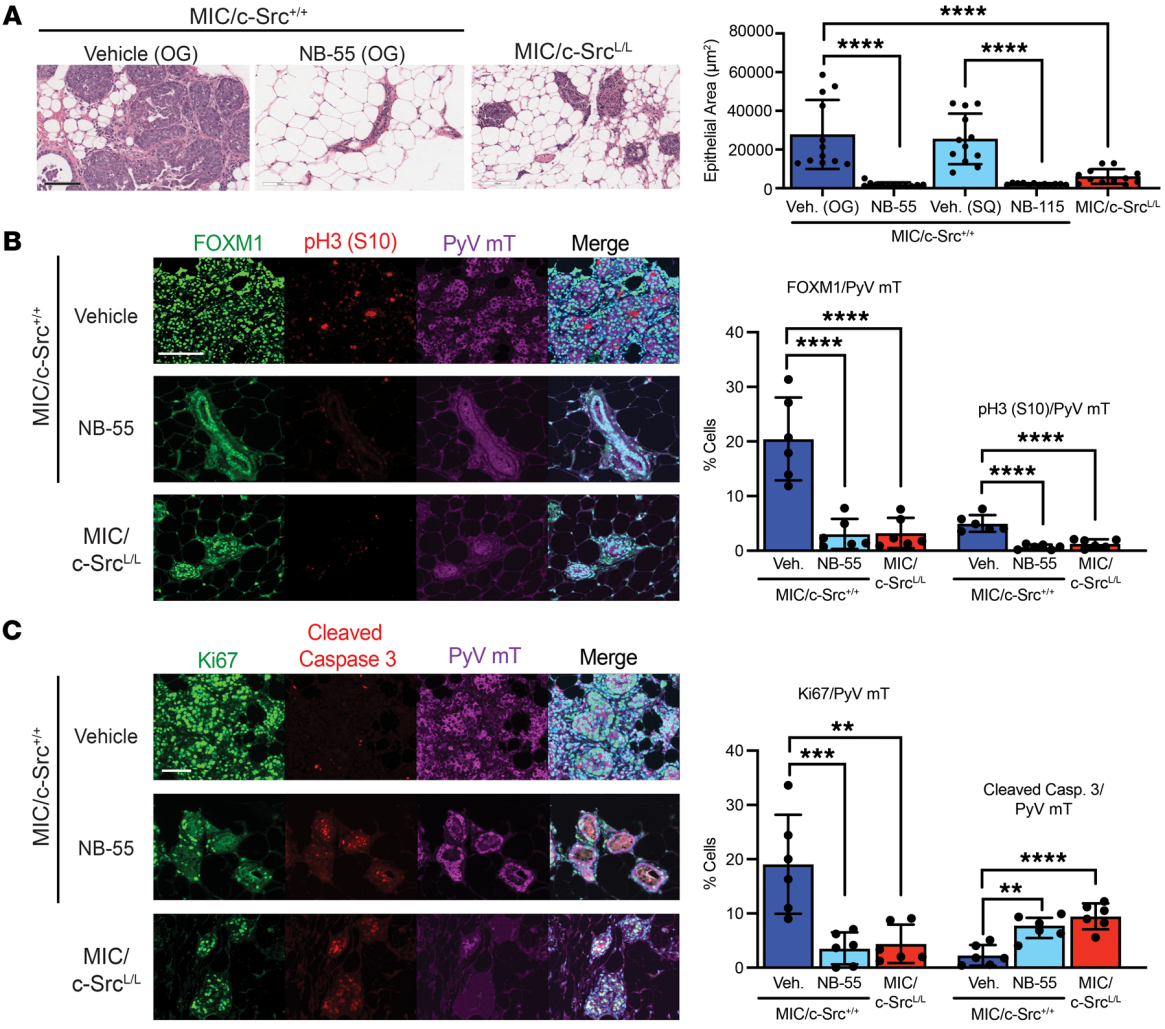
FOXM1 inhibition impairs in vivo cell-cycle progression and abrogates tumorigenesis. (**A**) H&E staining of mammary glands from MIC/c-Src^+/+^ mice treated with doxycycline for 2 weeks and then additionally with vehicle (Veh.) or a FOXM1 inhibitor (NB-55) by oral gavage (OG) for 3 weeks, or of mammary glands from MIC/c-Src^L/L^ mice treated with doxycycline for 5 weeks. Left panels: Images are representative of 6 mice per treatment group. Scale bar: 100 μm. Right panel: Mean area occupied by epithelial cells. *n* = 6 per group. ****P < 0.0001, by 1-way ANOVA with Tukey’s post hoc test. (**B** and **C**) Samples as in **A** were immunostained using the indicated antibodies and DAPI. Scale bars: 100 μm. Left panel: Images are representative of 6 mice per treatment group. Right panel: Quantification of immunostaining. **P < 0.01, ***P < 0.001, and ****P < 0.0001, by 1-way ANOVA with Tukey’s post hoc test.

**Figure 8:**
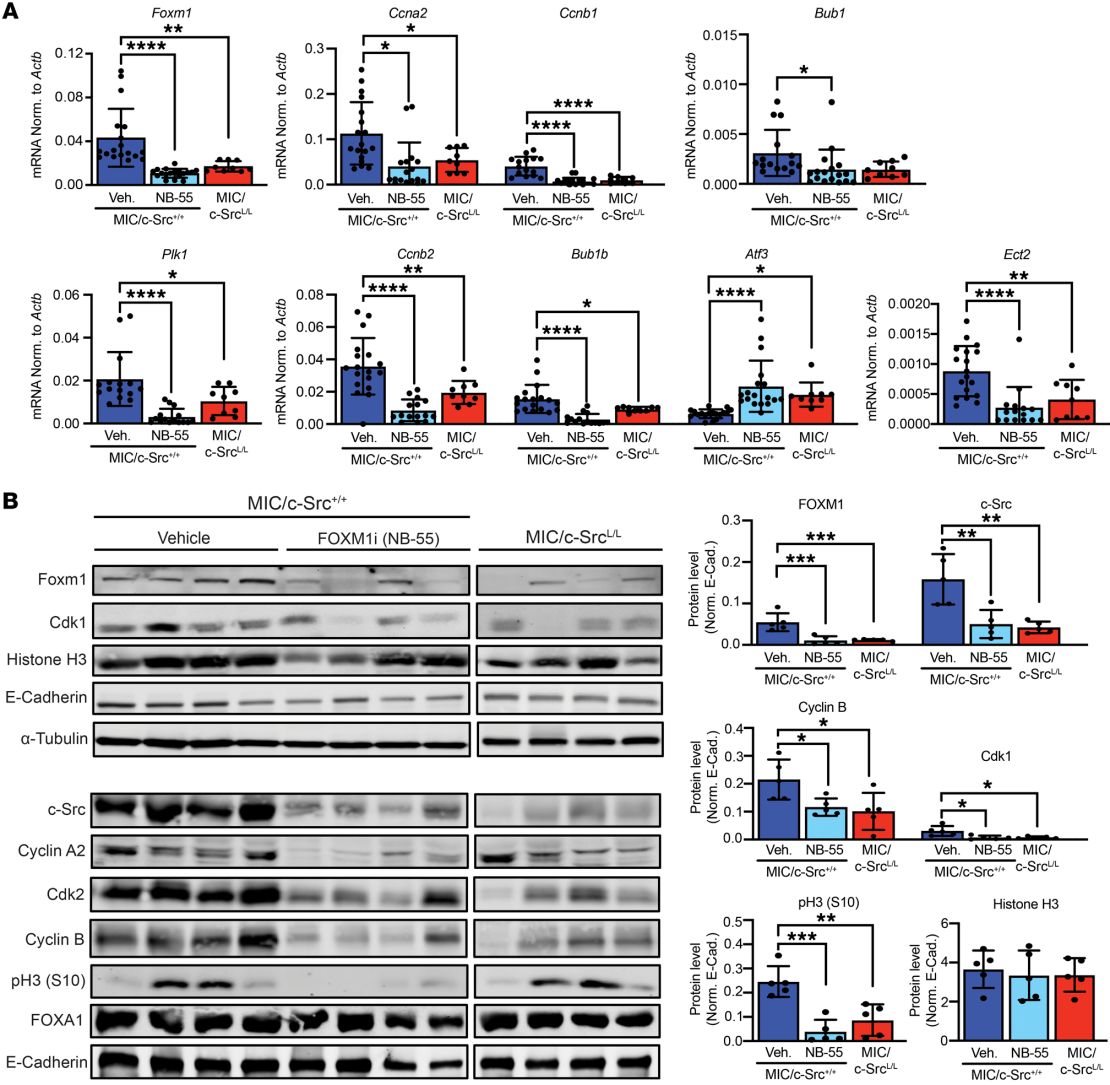
FOXM1 inhibition diminishes the expression of cell-cycle regulators in vivo. (**A**) qRT-PCR analysis of FOXM1 target gene expression in mammary gland samples from MIC/c-Src^+/+^ mice treated with vehicle or a FOXM1 inhibitor (NB-55) or from untreated MIC/c-Src^L/L^ mice. FOXM1 target expression was normalized to that of *Actb*. *n* = 6 per treatment group. *P < 0.05, **P < 0.01, and ****P < 0.0001, by 1-way ANOVA with Tukey’s post hoc test. (**B**) Lysates from mammary glands as in **A** were immunoblotted with the indicated antibodies. Left panel: Representative immunoblot. Right panel: Quantification (fluorescence immunoblotting). Data were normalized to E-cadherin (E-Cad.). *n* = 4 per genotype. *P < 0.05, **P < 0.01, and ***P < 0.001, by 1-way ANOVA with Tukey’s post hoc test.

To study FOXM1 inhibition in vivo, we treated MIC mice bearing palpable mammary lesions with NB-55, NB-115, or controls (Supplemental Figure 8A). FOXM1 inhibition significantly attenuated tumor progression (Figure 7A), phenocopying the largely normal ductal morphology of the MIC/c-Src^L/L^ mammary glands (Supplemental Figure 8, B and C), while reducing p-H3 (S10) and Ki67 levels and increasing apoptosis compared with vehicle controls (Figure 7, B and C). Inhibition of FOXM1 decreased its expression and that of its targets at the transcript and protein levels (Figure 8, A and B), without affecting its nuclear/cytoplasmic localization (Supplemental Figure 8D). Supporting the specificity of FOXM1 inhibitors, they did not alter the expression of FOXA1, a forkhead protein implicated in luminal breast cancers (54) (Figure 8B). Consistent with early metastatic dissemination in this model (25), in vehicle-treated MIC mice, we observed lung micrometastases that were largely absent in FOXM1 inhibitor–treated cohorts (Supplemental Figure 8E). In all quantitative analyses performed, differences in the phenotypes of FOXM1 inhibitor–treated mice and MIC/c-Src^L/L^ mice were not statistically significant, arguing that FOXM1 inhibition phenocopied c-Src ablation in this model. Stable silencing of FOXM1 in established PyVmT cells severely impaired orthotopic tumor outgrowth and prevented lung metastasis, correlating with suppression of in vitro migration and invasion (Supplemental Figure 9). Taken together, these findings support the efficacy of targeting FOXM1 in luminal B–like breast cancer.

**Figure 9:**
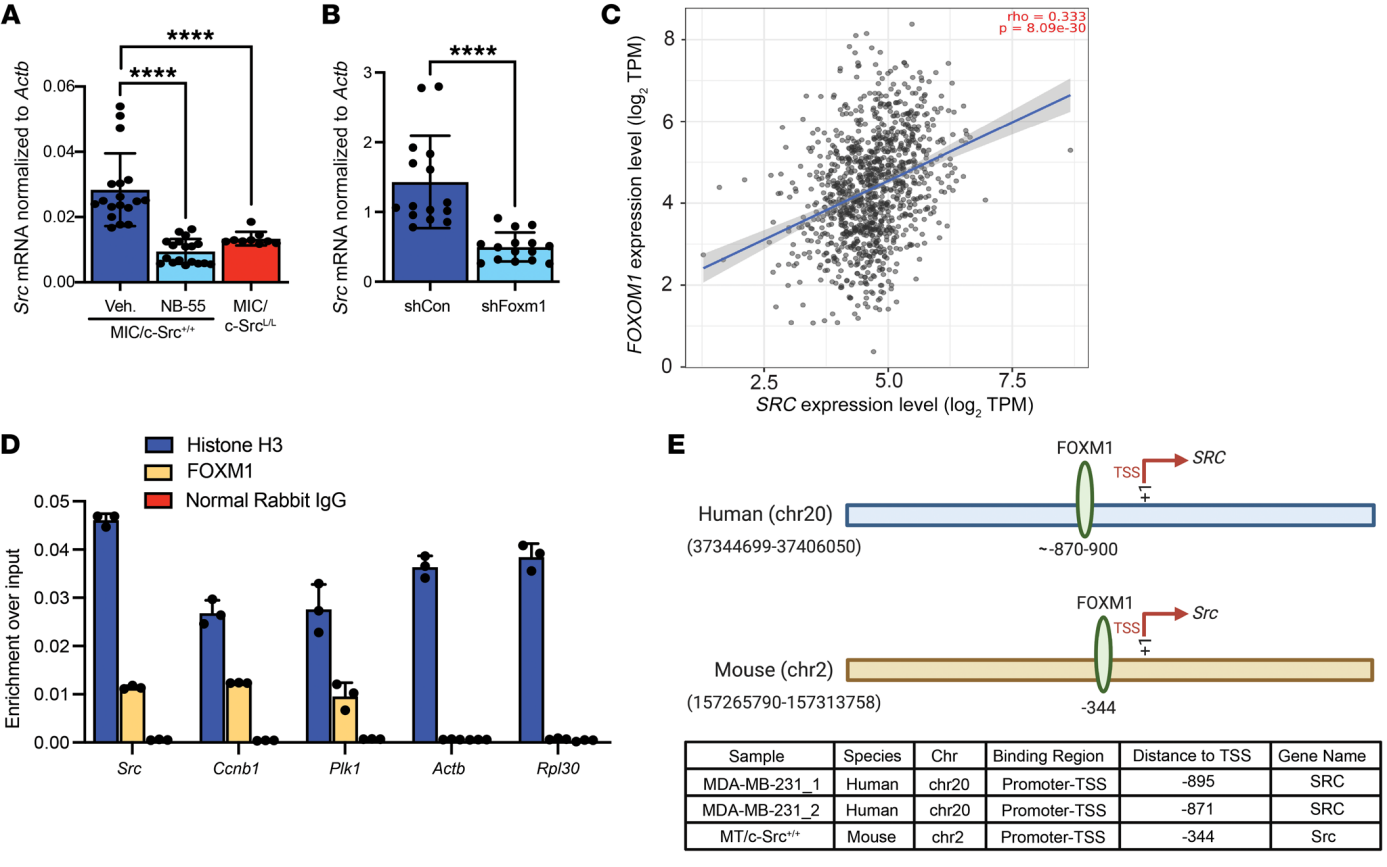
FOXM1 binds to sites in the *SRC* promoter and stimulates its expression in breast cancer. (**A**) qRT-PCR analysis of *Src* mRNA levels, normalized to *Actb*, in mammary glands from MIC/c-Src^+/+^ mice treated with vehicle or a FOXM1 inhibitor (NB-55) or from untreated MIC/c-Src^L/L^ mice. *n* = 6 per treatment group. ****P < 0.0001, by 1-way ANOVA with Tukey’s post hoc test. (**B**) qRT-PCR analysis of *Src* mRNA levels, normalized to *Actb*, in PyVmT tumors stably expressing the indicated shRNAs. *n* = 6 per treatment group. ****P < 0.0001, by unpaired, 2-tailed Student’s *t* test. shCon, control shRNA. (**C**) Correlation between *FOXM1* and *SRC* expression in a transcriptomic data set from a patient with breast cancer (*n* = 1,100; Spearman’s rank correlation analysis). TPM, transcripts per million. (**D**) ChIP and qRT-PCR analysis of FOXM1 binding to the promoters of the indicated genes in PyVmT breast cancer cells. *n* = 2 cell lines in triplicate. Anti–histone H3 antibody and normal rabbit IgG were used as positive and negative controls, respectively. (**E**) Schematic diagram illustrating FOXM1 binding to the promoter regions of human and murine *SRC*.

Throughout these studies, we observed that FOXM1 inhibition was associated with reduced c-Src protein expression (Figure 6D, Figure 8B, and Supplemental Figure 5B). We determined that this correlated with decreased steady-state levels of *Src* mRNA in PyVmT tumors upon FOXM1 inhibition or silencing (Figure 9, A and B). Accordingly, analysis of The Cancer Genome Atlas (TCGA) breast cancer data revealed that expression of *FOXM1* and *SRC* mRNAs correlated positively in patients’ tumor samples, particularly in luminal subtypes (Figure 9C and Supplemental Figure 10A). c-Src overexpression is well documented in many cancers, including breast cancer, where it correlates with disease progression and poor outcomes (5, 7), although the mechanisms are incompletely understood. Notably, FOXM1 binds genomic sites containing a forkhead consensus binding site (T/CAAACA) as well as a range of nonconsensus sites that do not contain this motif (45). Nonetheless, through genomic sequence analysis, we identified a candidate FOXM1-binding site 344 bp upstream of the murine *Src* transcription start site (TSS). Using a ChIP/quantitative reverse transcriptase PCR (qRT-PCR) strategy, we detected binding of FOXM1 to this site in PyVmT cells with enrichment similar to that of known FOXM1 targets (Figure 9D). We also observed that a peak indicating FOXM1 binding in the promoter region of human *SRC* was detected in publicly available ChIP-Seq data sets (55) (Figure 9E). Overall, these findings establish that c-Src is a direct transcriptional target as well as an upstream activator of FOXM1, indicating that a positive feedback loop may reinforce the expression and activity of both proteins in some breast cancer subtypes.

### The c-Src/FOXM1 axis is a therapeutic target that correlates with poor outcomes for patients with luminal B breast cancer.

**Figure 10:**
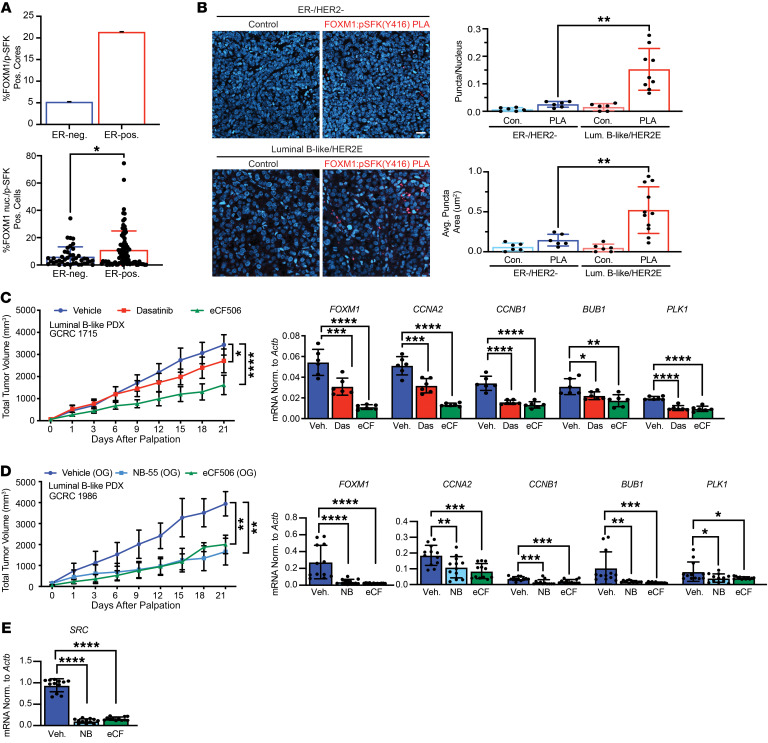
Inhibition of FOXM1 or c-Src impairs human luminal B breast cancer growth in vivo. (**A**) Quantification of FOXM1/p-SFK (Y416) double-positive cores and nuclear FOXM1/p-SFK (Y416) double-positive cells in ER^–^ (ER-neg.) (*n* = 76) and ER^+^ (ER-pos.) (*n* = 84) breast tumor samples from a TMA. (**B**) PLA of FOXM1/p-SFK (Y416) interaction in PDX models. Left panels: Images representative of PLA on 3 tumors from each model. Scale bar: 20 μm. Plot on the right shows quantification of the number and size of PLA puncta. Data are representative of 3 fields of view per tumor (minimum of 200 cells analyzed per sample). **P < 0.01, by 1-way ANOVA with Tukey’s post hoc test. Con., control; Lum., luminal. (**C**) Left panel: Tumor growth in PDX-bearing mice treated with the indicated SFK inhibitors or vehicle. *n* = 10 per treatment group. *P < 0.05 and ****P < 0.0001, by 1-way ANOVA with Dunnett’s post hoc test. Right panel: qRT-PCR analysis of the indicated mRNAs, normalized to *Actb*, in tumor samples. *n* = 6 per treatment group. *P < 0.05, **P < 0.01, ***P < 0.001, and *****P < 0.0001, by 1-way ANOVA with Dunnett’s post hoc test. Das, dasatinib; eCF, eCF506. (**D**) Left panel: Tumor growth in mice bearing luminal B–like PDX tumors and treated with a FOXM1 inhibitor (NB-55), SFK inhibitor (eCF506) or vehicle (*n* = 5 per treatment group). **P < 0.01, by 1-way ANOVA with Dunnett’s post hoc test. Right panel: qRT-PCR analysis of the indicated mRNAs in tumor samples. *n* = 4 per treatment group. *P < 0.05, **P < 0.01, ***P < 0.001, and ****P < 0.0001, by 1-way ANOVA with Dunnett’s post hoc test. (**E**) qRT-PCR analysis of *SRC* mRNA levels in PDX tumors as in **D**. *n* = 5 per treatment group. ****P < 0.0001, by 1-way ANOVA with Dunnett’s post hoc test.

We found that higher expression of *FOXM1* was associated with poor overall survival for ER^+^ breast cancers, which are mainly luminal tumors, from a large (*n* = 627) patient cohort (56) (Supplemental Figure 10B). Using TCGA breast cancer data (57), we demonstrated that a FOXM1 target gene signature (including *FOXM1*) was enriched in poor-outcome luminal B, HER2^+^, and triple-negative breast cancer (TNBC) subtypes, but not in luminal A or normal-like subtypes (Supplemental Figure 10C). After validating the specificity of the anti-FOXM1 antibody using tissue from tumors with FOXM1 silencing (Supplemental Figure 9E), we used it to show that nuclear FOXM1 protein expression correlated positively with SFK activity [p-SFK (Y416)] in ERα^+^Ki67^hi^ luminal B–like tumors (23, 24) from an independent cohort of 160 patients with breast cancer (Figure 10A and Supplemental Figure 11A). By using a PLA approach to interrogate patient-derived xenograft (PDX) models with distinct histopathological profiles (58), we confirmed the interaction between activated SFKs and FOXM1 in a PDX with features of aggressive luminal tumors (luminal cytokeratin expression and high Ki67 expression) but not in a PDX model of TNBC (Figure 10B). The SFKi dasatinib and eCF506 significantly attenuated tumor growth, downregulated *FOXM1* expression, and induced gene expression changes consistent with FOXM1 inhibition in the luminal-like PDX (Figure 10C and Supplemental Figure 11B). Both the FOXM1 inhibitor NB-55 and the SFKi eCF506 also significantly impaired the growth of a second PDX model established from a luminal B–like tumor (58) (Figure 10D), suppressed the expression of *FOXM1* and its targets, including *SRC*, and significantly reduced lung metastasis in this model (Figure 10, D and E, and Supplemental Figure 11, C and D). These findings further support the therapeutic potential of targeting the c-Src–FOXM1 feedback loop in luminal B–like breast cancer.

## Discussion

**Figure 11:**
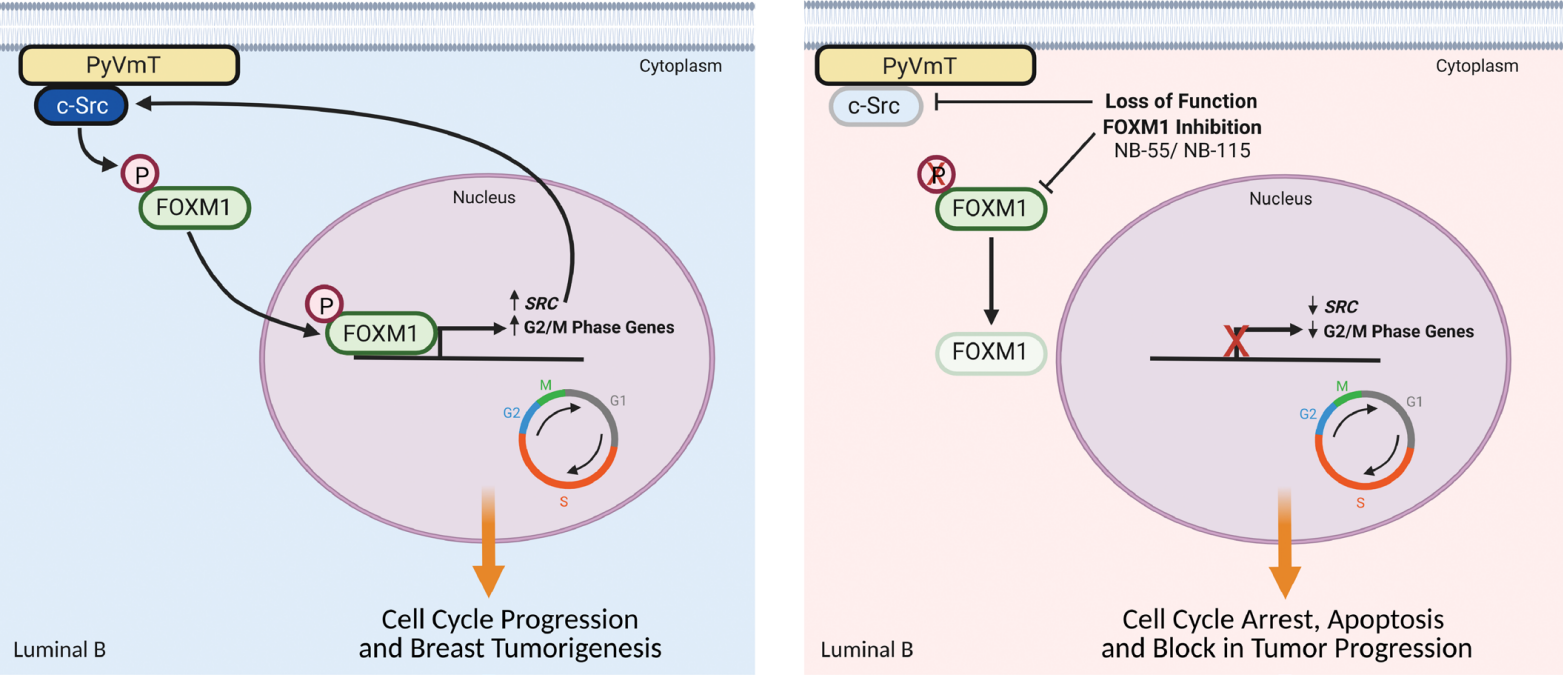
Coordinated c-Src/FOXM1 activity drives breast cancer progression. Left panel: Schematic diagram illustrating coordinated activation of c-Src and FOXM1 in breast cancer, leading to cell-cycle deregulation, proliferation, and tumor progression. Right panel: Loss of c-Src–mediated phosphorylation of FOXM1 or pharmacological/genetic targeting of FOXM1 blocks the cell cycle by suppressing the expression of G_2_/M-phase genes and of c-Src itself, disrupting positive feedback and impairing the progression of luminal B–like breast cancers.

Early studies associated the v-Src oncogene with cell-cycle deregulation and proliferation (59–61). Although mutations constitutively activating c-Src are very rare, c-Src overexpression and activation in cancer has multifaceted and context-dependent effects on the cell cycle (17, 62–65). Here, we have shown that activated c-Src interacts with and phosphorylates the transcription factor FOXM1 on 2 sites, Y239 and Y517, promoting its nuclear localization and activation of target gene transcription. The c-Src/FOXM1–dependent transcriptional program includes many key regulators of G_2_/M and mitotic progression as well as c-Src itself, forming a positive feedback loop that drives breast cancer cell proliferation and metastasis (Figure 11). Importantly, while our data argue that c-Src directly regulates FOXM1, they do not rule out the involvement of adaptor or scaffold proteins in mediating the interaction between the 2 proteins or preclude a role for other mechanisms. For example, tyrosine phosphorylation of FOXM1 may affect the phosphorylation of proximal serine/threonine residues and subsequent recruitment of the phosphoserine/threonine-binding protein 14-3-3ζ, which is a c-Src target that promotes FOXM1 function (66, 67).

Luminal B–like breast cancers present a significant clinical challenge due to their high rate of proliferation, low expression of hormone receptors, and tendency to resist endocrine and other therapies. Targeting c-Src in multiple preclinical models of luminal B–like breast cancer induced cellular fates incompatible with tumor progression, including G_2_/M cell-cycle arrest and apoptosis. This is consistent with recent studies indicating that SFKi, which have a spectrum of SFK and non-SFK targets, may be efficacious in advanced, endocrine therapy–resistant breast cancer, particularly in combination with other therapies (68). However, clinical trials of SFKi in solid tumors have yielded mostly mixed or poor results (69–71), possibly due to their lack of specificity, which may lead to dose-limiting toxicities or complex effects on both tumor and stromal cells, including immune cells (72, 73), that may limit efficacy. Newer drugs such as eCF506, which binds to and stabilizes the inactive conformation of SFKs to achieve specificity superior to ATP-competitive inhibitors (48, 74), are a significant advance. However, specific targeting of individual SFKs remains a considerable challenge. Identifying and targeting essential functions downstream of c-Src therefore remains important in developing effective therapeutic strategies.

The data presented here argue that coordinated activation of c-Src and FOXM1 is a targetable vulnerability in luminal B–like breast cancer. Although transcription factors are often considered “undruggable” because of their lack of defined binding pockets where small molecules could disrupt function, important in chemical biology work has identified compounds with an “induced degradation” mechanism of action that does not require a focus on any specific binding site (75). This approach, which engages cellular protein quality control machinery to downregulate targets, has shown considerable promise for inhibiting transcription factors. For example, the selective estrogen receptor degraders (SERDs), such as fulvestrant (76), bind to ERα and induce structural changes that result in its proteosome-mediated degradation. Importantly, FOXM1 acts as both a transcriptional activator and a repressor, with the latter activity recently shown to support metastatic progression in a model of luminal B breast cancer (77). Induced degradation of FOXM1 is therefore an attractive strategy, as it would interfere with both aspects of FOXM1 function. We demonstrate that recently discovered compounds that bind FOXM1 and trigger small-molecule–induced degradation via the proteasome (50) are effective across multiple models of luminal B–like breast cancer, establishing a potential indication for the clinical translation of these compounds. These findings are highly topical, given that existing strategies targeting aberrant cell-cycle regulation such as CDK4/-6 inhibitors, which have become the standard of care for metastatic hormone receptor–positive cancers, are limited by the development of resistance (3, 78, 79) and may be less effective against luminal B–like tumors compared with luminal A types (80, 81). By providing an alternative means of targeting the cell-cycle deregulation and accelerated proliferation typical of aggressive luminal breast cancers, interfering with the c-Src–FOXM1 positive feedback loop may improve patient outcomes.

## Methods

### Animal models

For all mammary tumor studies, palpation to detect tumor onset and caliper measurements of tumors were performed twice weekly. Mice were euthanized according to approved facility protocols when their tumor volume reached 2.5 cm^3^ for a single mass or a total of 5 cm^3^ for multifocal tumors.

#### Transgenic models.

MIC, MMTV-PyVmT, MMTV-rtTA, and *Src-*conditional mice (9, 20, 25, 26) were on a pure FVB/ NJ (Friend leukemia virus B/NIH Jackson) background and were genotyped by qRT-PCR (see Supplemental Table 1). All transgenic mice were generated through an in-house breeding program. Female littermates were group-housed under specific pathogen–free conditions with a 12-hour day/23-hour night cycle and ad libitum access to food and water. Transgene expression was induced in 8- to 12-week-old MIC mice via administration of 2 mg/mL doxycycline (Wisent, 450-185-EG) in light-blocking bottles.

#### PDX models.

Freshly excised pieces of early-passage PDX tumors of approximately 8 mm^3^ in size were implanted into the left inguinal mammary fat pad of 12-week-old female NOD/SCID/γ (NSG) immunocompromised mice (Charles River Laboratories).

#### Experimental lung metastasis.

A total of 250,000 cells were suspended in 100 μL PBS and injected into the tail vein of female athymic nude (NCr) mice (Taconic). After 3 weeks, mice were euthanized, and lung tissues were collected for histological examination.

#### Orthotopic allografts.

A total of 500,000 cells were suspended in 30 μL PBS and injected s.c. into the mammary fat pads of 12-week-old female NSG mice (Charles River Laboratories).

#### In vivo therapeutic studies.

Mice were randomly assigned to treatment groups and weighed twice weekly, with the doses adjusted according to body weight. Drug administration, tumor measurement, and data analysis were performed by different individuals who were each blinded to the treatment groups. Treatment of PDX models was initiated when tumors reached 65 mm^3^, with termination of the experiment when all vehicle controls had reached the endpoint. Eight-week-old MIC mice were treated with drugs following 2 weeks of doxycycline administration, and experiments were terminated after 3 weeks. Dasatinib (MedChemExpress, HY-10181) and eCF506 (MedChemExpress, HY-112096) were formulated in 80 mM citric acid (made in water) and administered daily by oral gavage at 10 mg/kg. NB-55 and NB-115 were gifts of John and Belinda Katzenellenbogen (University of Illinois at Urbana Champaign, Champaign, Illinois, USA). NB-55 was dissolved in a vehicle containing 9/0.5/0.5/90 parts PEG400/Tween-80/povidone/0.5% carboxymethylcellulose in deionized water and administered daily by oral gavage at 100 mg/kg (50). NB-115 was dissolved in 10% DMSO and 90% corn oil and administered daily by s.c. injection at 6 mg/kg (50).

### Histology and immunostaining

Tissues were fixed for 24 hours in 10% neutral buffered formalin, paraffin embedded, and sectioned at 4 μm. Sections were stained with H&E or processed further as indicated.

#### Lung metastasis.

Three H&E-stained 10 μm step sections per sample were scanned using an Aperio XT slide scanner (Leica Biosystems) and analyzed using ImageScope software (Leica Biosystems).

#### BrdU Incorporation.

BrdU (GE Healthcare, RPN 201) was administered at 0.02 mg/g bodyweight by i.p. injection. Tissues were collected for analysis 2 hours after injection.

#### Immunofluorescence.

Sections were deparaffinized in xylene, and antigen retrieval was performed in 10 mM EDTA (pH 9) using a pressure cooker. Sections were treated with 10% Power Block (BioGenex, HK083) in TBS for 10 minutes at room temperature and then with a primary antibody in 2% (wt/vol) BSA in PBS at 4°C overnight. Secondary antibody incubation was performed with ImmPRESS polymer detection kit (Vector Laboratories, VECTMP745250, VECTMP740150, VECTMP740450) and tyramide signal amplification substrates (Akoya Biosciences, OP-001001, OP-001303; PerkinElmer, FP1489). Three washes in PBS were performed between each step. Slides were incubated with DAPI (Thermo Fisher Scientific, D1306) for 10 minutes at room temperature, washed 3 times in water, mounted in ImmuMount (Thermo Fisher Scientific, 9990412), imaged using a Zeiss LSM800 confocal microscope or a Zeiss Axioscan slide scanner, and analyzed using HALO software (Indica Labs) with quantification of 5 independent fields of view from 4 mice per experimental group. The primary antibodies used are detailed in Supplemental Table 2.

### Tissue microarrays

Tissue microarrays (TMAs) were obtained from US Biomax (BR2082c, BR1503f, and BR806). The status of the ER, PR, HER2, and Ki67 samples is publicly available: BR2082c, https://www.tissuearray.com/tissue-arrays/Breast/BR2082c; BR1503f, https://www.tissuearray.com/tissue-arrays/Breast/BR1503f; and BR806, https://www.tissuearray.com/tissue-arrays/Breast/BR806 Sections were cut at 4 μm, mounted onto glass slides, and baked for 2 hours at 79°C. Immunofluorescence was performed and quantified as described above.

### Mammary gland whole mounts

Inguinal mammary glands were excised, placed on glass slides, fixed overnight in acetone, stained in hematoxylin for 24 hours, destained in 70% ethanol/1% HCl, washed in 100% ethanol, dehydrated overnight in xylenes, mounted using Permount (Thermo Fisher Scientific, SP15-100), and imaged using a Zeiss AxioZoom V16 microscope.

### Cell culture

Mammary tumors 8 weeks after palpation were excised from female MMTV-PyMT mice (20), dissociated in collagenase B (Roche, 11088831001)/Dispase II (Roche, 4942078001) (2.4 mg/mL each) for 2 hours at 37°C, washed 3 times with PBS and 1 mM EDTA, and plated in complete media consisting of DMEM (Wisent, 319-005-CL) supplemented with 2% FBS (Wisent, 080-150), 5 ng/mL EGF (Wisent, 511-110-UM), 1 μg/mL hydrocortisone (MilliporeSigma, H4001), 5 μg/mL insulin (Wisent, 511-016-UG), 35 μg/mL bovine pituitary extract (BPE) (Hammond CellTech, 1078-NZ), and 50 μg/mL penicillin/streptomycin (Wisent, 450-200-EL). Cells were maintained in a humidified 5% CO_2_, 37°C incubator in complete media. Transduction with the adenoviruses Ad5CMVCre and Ad5CMVCytoLacZ (University of Iowa Viral Vector Core, Iowa City, Iowa, USA) was done according to the manufacturer’s instructions at a MOI of 25. Cells were authenticated by PCR-based genotyping (oligonucleotide details are provided in Supplemental Table 1) and by immunoblotting to detect c-Src expression. The human cell lines 293T (CRL-3216) and MCF7 (HTB-22) were purchased from ATCC, used at early passage, and maintained in DMEM with 10% FBS. All cell lines were tested biweekly for mycoplasma using the MycoAlert Kit (Lonza, LT07-118), and all cells used in this study were negative for mycoplasma contamination.

### In vitro migration and invasion assays

Boyden chambers (8 μm pore, BD Falcon) were coated with 50 μL DMEM (migration) or DMEM containing 5% growth factor–reduced Matrigel (invasion) (BD, 356234) in the upper chamber and incubated in 24-well plates (Falcon, 353047) with 1 mL complete media in the lower chamber for 1 hour at 37°C. Cells (150,000 cells/500μL in DMEM) were plated in triplicate in the upper chamber and incubated for 24 hours at 37°C in 5% CO_2_. Boyden chambers were then fixed in 10% neutral-buffered formalin for 30 minutes, washed 3 times with water, and stained with crystal violet for 30 minutes. Images were acquired using an AxioZoom V16 (Zeiss) and analyzed (positive pixel) with ImageJ software (NIH).

### 3D organotypic culture

3D organotypic cultures were prepared as described elsewhere (30, 82). Briefly, mammary glands from 8- to 12-week-old mice were dissociated in 10 mL DMEM/F12, 1× penicillin/streptomycin, 50 mg/mL gentamycin (Wisent, 450-135-XL), and 2 mg/mL collagenase B (Roche, 11088831001) for 1 hour at 37°C, and then washed in PBS with 5% FBS (Wisent, 080-150), centrifuged at 1,500*g* for 15 seconds, and resuspended in 0.25% trypsin (Wisent, 325-143-ES) for 20 minutes at 37°C. Trypsin was quenched with FBS, and cells were resuspended in 3D medium consisting of Epicult-B (STEMCELL Technologies, 05610), with 1% (vol/vol) knockout serum replacement (Gibco, Thermo Fisher Scientific, 10828010), 50 μg/mL penicillin/streptomycin (Wisent, 450-200-EL), 10 ng/mL EGF, 25 μg/mL insulin, 1 μg/mL hydrocortisone, and 2% Geltrex (Thermo Fisher Scientific, A1413202) and then filtered through a 40 μm mesh. Single cells (10,000 cells/well) were plated on Geltrex-coated coverslips in a 24-well plate and grown in 3D medium until organoids formed (5–7 days), and then MIC transgene expression was induced by treatment with 2 μg/mL doxycycline for 12 days.

### Immunofluorescence staining of 3D cultures

Organotypic cultures were fixed in 4% paraformaldehyde for 20 minutes at room temperature, permeabilized in 1× PBS with 0.2% Triton X-100 for 20 minutes at room temperature, blocked with Immunofluorescence (IF) Buffer (1× PBS with 0.2% Triton X-100, 0.05% Tween-20, and 2% BSA), and incubated with primary antibodies (described above) overnight at 4°C in IF Buffer. Samples were stained with secondary antibodies in IF Buffer for 45–60 minutes at room temperature and with DAPI (1 μg/mL in water) for 20 minutes at room temperature. Three washes in IF buffer were performed between each step. Organoids were mounted onto slides with ImmunoMount and imaged using a Zeiss LSM800 confocal microscope. Staining was quantified in at least 5 organoids (>20 nuclei per organoid) per condition using HALO software. The primary and secondary antibodies used are detailed in Supplemental Table 2.

### qRT-PCR

Total RNA was extracted from cultured cells, organoids, or flash-frozen tissue using an RNeasy Mini Kit (QIAGEN, 74106). cDNA was prepared using the ProtoScript First Strand cDNA Synthesis Kit (New England BioLabs, E6300). Real-time qRT-PCR was performed on the LightCycler 480 instrument (Roche) using LightCycler 480 SYBR Green 1 MasterMix (Roche, 04887352001) and analyzed using the associated software. Samples were run in triplicate and normalized to *Actb* as a control. The primer sequences are listed in Supplemental Table 3.

### Transcriptomic analysis

RNA was isolated as described above from 3 independent pools of organoids per genotype, with and without doxycycline induction, and quality was assessed using a NanoDrop 2000 (Thermo Fisher Scientific, ND2000CLAPTOP). RNA was sequenced and analyzed as previously described (10). Differential expression analysis was performed using the DEGseq2 R package (2_1.6.3), with *P* values adjusted using Benjamini and Hochberg’s method. Genes with adjusted *P* values of less than 0.05 were considered to be differentially expressed. Analysis of transcriptional regulation and pathway representations in differentially expressed genes was performed using Enrichr (83).

### Flow cytometry

Cells were treated with 10 μm 5-ethynyl-2′-deoxyuridine (EdU) (MedChemExpress, HY-118411) for 3 hours. For in vivo analysis, doxycycline was administered to MIC mice for 4 weeks, at which point they were injected with EdU (0.1 mg/1 g body weight) for 3 hours. Mammary glands were then excised and dissociated as described above. Samples were washed with FACS buffer (1× PBS with 2 mM EDTA and 2% FBS) passed through a 70 μm strainer and incubated with TruStain FcX block (BioLegend, 422302). Cells were incubated with anti–PyV mT antibody for 30 minutes on ice and washed twice with FACS buffer prior to fixation. For all other antigens, cells were fixed in BD Cytofix Fixation Buffer (BD, 554655) for 15 minutes at room temperature, washed twice with BD Perm/Wash (BD, 554723), incubated with primary antibodies for 1 hour at room temperature, and incubated with 5 μg/mL 7-amino-actinomycin D (7-AAD) (BioLegend, 420403) in FACS buffer to stain DNA. EdU incorporation was detected using the Invitrogen Click-iT EdU Alexa Fluor 488 Flow Cytometry Kit according to the manufacturer’s instructions (Thermo Fisher Scientific). A minimum of 250,000 events per sample were acquired using a LSR Fortessa Flow Cytometer with FACSDiva Software, version 8 (BD Biosciences) in slow-rate mode to avoid doublets. Cell populations were gated as shown in Supplemental Figure 3B. Data were analyzed with FlowJo Software. Cell debris and aggregates were excluded from the analysis using pulse processing SSC-H vs SSC-W and 7-AAD-A versus 7-AAD-W. Supplemental Table 4 provides antibody and reagent details.

### PLA

Tissue sections and cells were prepared as described above for IF staining and incubated with antibodies against FOXM1 (Santa Cruz Biotechnology, sc-376471, 1:50) and p-Src family kinases (Y416) (Cell Signaling Technology, 2101, 1:200). PLA probe hybridization, ligation, and amplification were performed according to the manufacturer’s protocol (DuoLink In Situ, MilliporeSigma, DUO92008). For mouse and human samples, mouse IgG1 (Cell Signaling Technology, 2367) and rabbit IgG (Cell Signaling Technology, 3724) isotype controls were used. Fluorescent signals were quantified using HALO software.

### Protein extraction and immunoblotting

Excised tumor tissue was flash-frozen in liquid nitrogen, crushed under liquid nitrogen, allowed to thaw briefly, and then lysed in ice-cold RIPA buffer containing 50 mM Tris-HCl, pH 7.4, 150 mM sodium chloride, 1% Nonidet P-40, 1% sodium deoxycholate, 0.1% SDS, 2 mM EDTA, 0.5 mM AEBSF (Santa Cruz Biotechnology, sc-202041), 25 mM β-glycerophosphate (MilliporeSigma, G5422), 1 mM sodium orthovanadate (BioShop, SOV664), and 10 mM sodium fluoride (MilliporeSigma, S7920)). Cultured cells were lysed in RIPA buffer. For immunoblotting of histones, the Episeeker Histone Extraction Kit (Abcam, ab113476) was used to extract histones from 5 × 10^6^ to 6 × 10^6^ cells according to the manufacturer’s protocol. Lysates were cleared by centrifugation at 4°C and 15,000*g* for 10 minutes, protein concentrations were determined by Bradford assay (Bio-Rad, 5000006), and 40 μg total protein was analyzed by SDS PAGE and fluorescence immunoblotting using the Li-COR Odyssey system and associated software (Li-COR Biosciences). The primary and secondary antibodies used are detailed in Supplemental Table 2. (See supplemental materials for uncropped images of all immunoblots.) 

### Immunoprecipitation

Cell lysates were prepared as described above, diluted with lysis buffer to 500 μg total cellular protein in a volume of 1 mL, and incubated with 1 μg antibody against FOXM1 (Proteintech, 13147-1-AP), c-Src (Santa Cruz Biotechnology, sc-8056), or FLAG (Cell Signaling Technology, 14793) overnight at 4°C with end-over-end mixing. Normal rabbit IgG (Cell Signaling Technology, 2729) was used as a negative control. Immunoprecipitates were incubated with PureProteome protein A/G magnetic beads (MilliporeSigma, LSKMAGAG10) at 4°C for 1 hour, washed 5 times in lysis buffer, and analyzed by SDS PAGE and immunoblotting as described above.

### ChIP

MT/c-Src^+/+^ cells in 15 cm plates (Nunc, 168381) were fixed in 1% formaldehyde for 10 minutes, and the SimpleChIP Enzymatic Chromatin IP Kit (Cell Signaling Technology, 9003) was used to prepare chromatin according to the manufacturer’s protocol. Equal amounts of digested, cross-linked chromatin were diluted in 1× ChIP Buffer (Cell Signaling Technology, 7008) and incubated with antibodies against FOXM1 (Proteintech, sc-376471, 1:100), Histone H3 (Cell Signaling Technology, 4620, 1:50), or normal rabbit IgG (Cell Signaling Technology, 2729, 1:500) overnight at 4°C with end-over-end mixing, and then incubated with ChIP-Grade Protein G Magnetic Beads (Cell Signaling Technology, 9006) for 2 hours at 4°C with end-over-end mixing. Beads were washed 4 times with ChIP Buffer, and DNA was eluted with 50 μL DNA Elution Buffer (Cell Signaling Technology, 10009). ChIP enrichment was quantified by qRT-PCR as described above (primer details are provided in Supplemental Table 5). *CCNB1* and *PLK1* were used as positive controls and *ACTB* and *RPL30* as negative controls for FOXM1 binding.

### Plasmid constructs, site-directed mutagenesis, and lentiviral transduction

pCW57.1-FOXM1c (Addgene, 68810) was used to express FLAG-tagged human FOXM1c in mammalian cells in a doxycycline-inducible manner (43). The Q5 Site-Directed Mutagenesis Kit (New England BioLabs, E0554) was used according to the manufacturer’s instructions to mutate tyrosine to phenylalanine. Mutations were confirmed by Sanger sequencing of the full FOXM1 sequence (see Supplemental Table 6 for the list of mutagenesis and sequencing primers). Lentiviruses were produced in HEK293T cells by cotransfection with pMD2.G (Addgene, 12259) and psPAX2 (Addgene, 12260) using Lipofectamine 3000 (Invitrogen, Thermo Fisher Scientific, L3000075) according to the manufacturer’s protocol. Virus-containing media were harvested 24 hours and 48 hours after transfection and filtered through a 0.45 μm filter. Cells were transduced with lentiviruses in the presence of 10 μg/mL polybrene (MilliporeSigma, 107689) and selected with and maintained in complete media with 2 μg/mL puromycin (BioShop, PUR333).

### Cellular fractionation

The NE-PER Cell Fractionation Kit (Thermo Fisher Scientific, 78833) was used according to the manufacturer’s instructions. Fractions and whole-cell lysate, collected simultaneously at the time of fractionation, were analyzed by immunoblotting as described above.

### In vitro kinase assay

HEK293T cells were transiently transfected with pCW57.1 constructs bearing WT FOXM1c or Y-F mutants, and expression was induced by treatment with doxycycline for 72 hours. Cells were lysed and anti-FLAG immunoprecipitation was performed as above. Immunoprecipitates were exchanged into a buffer containing 20 mM HEPES (pH 7.5), 150 mM NaCL, 5 mM MgCl_2_, 2 mM MnCl_2_, 1 mM DTT, and 100 μmol/L ATP, and 200 ng purified active c-Src kinase (MilliporeSigma, 14-326) was added to a total reaction volume of 20 μL. Reactions were incubated at 37°C for 2 hours and terminated by adding SDS-PAGE lysis buffer, vortexing, and boiling for 10 minutes. Assays were analyzed by immunoblotting as described above.

### RNAi and lentiviral transduction

Sigma MISSION pLKO.1 constructs harboring shRNAs against mouse FOXM1 (clone IDs: shFOXM1-1, TRCN0000084773; shFOXM1-2, TRCN0000084774) and the nonmammalian target luciferase (clone ID: TRCN0000072259) were obtained from the McGill Platform for Cellular Perturbation. The shRNA sequences were subcloned into the pLKO.1-Blast vector (oligonucleotides are listed in Supplemental Table 7). Lentivirus production and cellular transduction were performed as described above, with cells selected and maintained in complete media with 8 μg/mL blasticidin (BioShop, BLA477).

### In vitro drug and IncuCyte cell proliferation assays

NB-55 and NB-115 were reconstituted in DMSO as described previously (50). For immunoblotting and qRT-PCR, cells were treated at the indicated concentrations for 24 hours. For proliferation assays, 5,000 cells/well were seeded in triplicate in 96-well, optical-bottomed plates (Nunc, 167008), drugs or vehicle controls were added after 24 hours, and the IncuCyte S3 system (ESSEN BioSciences) was used for live cell imaging at ×10 magnification every 6 hours for a total of 96 hours (*n* = 2 images per well per time point; *n* = 17 total time points). The confluence percentage was determined using IncuCyte S3 Analysis software.

### Patient data analysis

FOXM1 ChIP-Seq data from the NCBI’s Gene Expression Omnibus (GEO) Accession Viewer database (GEO GSE72977) (55) were used to identify FOXM1 peaks (±50 kb from the TSS). To correlate *FOXM1* and *SRC* expression, we used TIMER2.0 (84). Kaplan-Meier survival curves were generated from overall survival data using KM Plotter (85). Transcriptomic and reversed-phase protein array (RPPA) data from The Cancer Genome Atlas (TCGA) were accessed using UCSC Xena (86).

### Data availability

RNA-Seq data have been deposited in the NCBI’s GEO Database (GEO GSE224876).

### Statistics

An unpaired, 2-tailed Student’s *t* test or 2-way ANOVA with Dunnett’s or Tukey’s post hoc test for multiple comparisons was performed as appropriate using GraphPad Prism 9 (GraphPad Software) or Microsoft Excel. Kaplan-Meier analysis with a log-rank test (Mantel-Haenszel) was performed using GraphPad Prism 9. Throughout the study, the data are plotted as the mean ± SD, and a *P* value of less than 0.05 was considered significant. Statistical comparisons not indicated in the figures were found to be insignificant.

### Study approval

Experiments involving mice were conducted in accordance with McGill University and the Canadian Council on Animal Care (CCAC) ethics guidelines under protocol MCGL-5518, approved by the McGill University Downtown Campus Facility Animal Care Committee (FACC), a branch of the McGill University Animal Care Committee (UACC) (Montreal, Quebec, Canada). Human tumor samples, including those used for PDX models (58), were collected with written and informed consent at the McGill University Health Center using protocols (A08-M39-19B) approved by the Research Ethics Office and the IRB of the McGill University Faculty of Medicine and Health Sciences.

## Author contributions

IN, HWS, and WJM conceptualized the study. IN, HWS, VSG, LJ, SSA, SN, VP, AP, DZ, and SL performed experiments. IN, HWS, VSG, SN, and AP performed formal analysis. SHK, JAK, BSK, HK, AMF, PS, CMR, and MP provided resources. IN, HWS, and WJM wrote the original draft of the manuscript. IN, HWS, BSK, JAK, and WJM reviewed and edited the manuscript. IN, HWS, VSG, and AP prepared figures. IN, HWS, BSK, JAK, and WJM acquired funding. HWS and WJM supervised the study. IN, and HWS share first authorship. The order of the first authors’ names was assigned alphabetically.

## Supplementary Material

Supplemental data

## Version 1 02/16/2023

In-Press Preview

## Version 2 04/03/2023

Electronic publication
