# Supplementary material for: Coordinated activation of c-Src and FOXM1 drives tumor cell proliferation and breast cancer progression
Source: J Clin Invest. 2023 Apr 3;133(7):e162324. doi: 10.1172/JCI162324 (PMC10065076; doi:10.1172/JCI162324)
Supplement: Supplemental data [file jci-133-162324-s167.pdf]

Supplemental Data

Coordinated Activation of c-Src and FOXM1 Drives Tumor Cell Proliferation and Breast Cancer Progression

Nandi et al.

- Supplemental Figures 1-11
- Supplemental Tables 1-7
- Uncropped immunoblot images

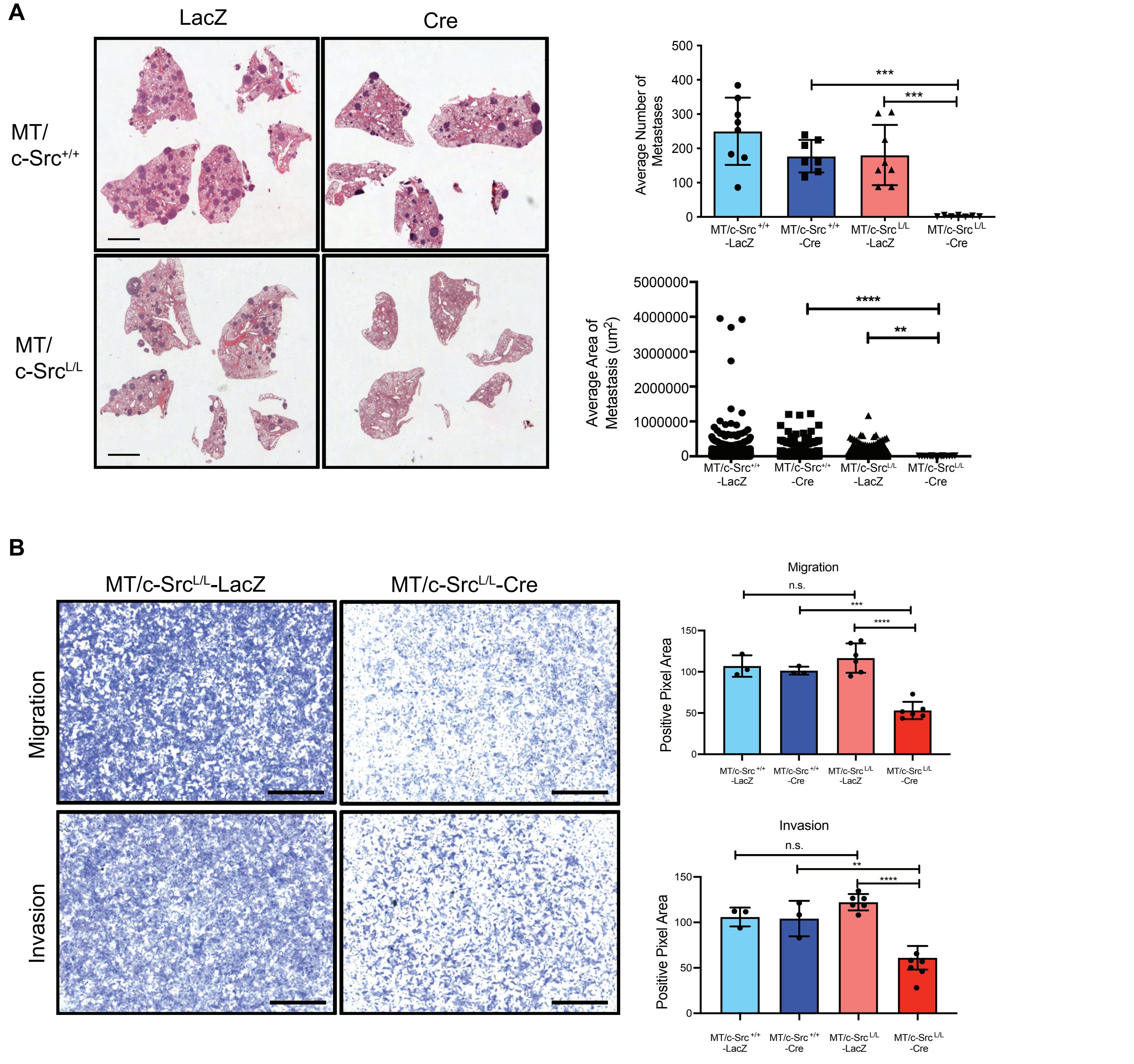

Supplemental Figure 1: Acute c-Src ablation decreases the metastatic capacity of PyV mT cells.

(A) Left panel – representative H&E staining of lungs 3 weeks post-tail vein injection of MT/c-Src$^{+/+}$ and MT/c-Src$^{L/L}$ cells transduced *in vitro* with adenoviruses bearing Cre recombinase or LacZ. Scale bar represents 5mm. Right panel – quantification of lung lesions (n = 8 mice per genotype – **$p < 0.01$, ***$p < 0.001$, ****$p < 0.0001$; one-way ANOVA with Tukey's post hoc-test). **(B)** Left panel –Representative images of cell migration and invasion (Boyden chamber) assays. Scale bar represents 1000 µm. Right Panel – quantification (positive pixel area) of cell migration and invasion (n = 2 cell lines per genotype in triplicate – **$p < 0.01$, ***$p < 0.001$, ****$p < 0.0001$; one-way ANOVA with Tukey's post hoc-test).

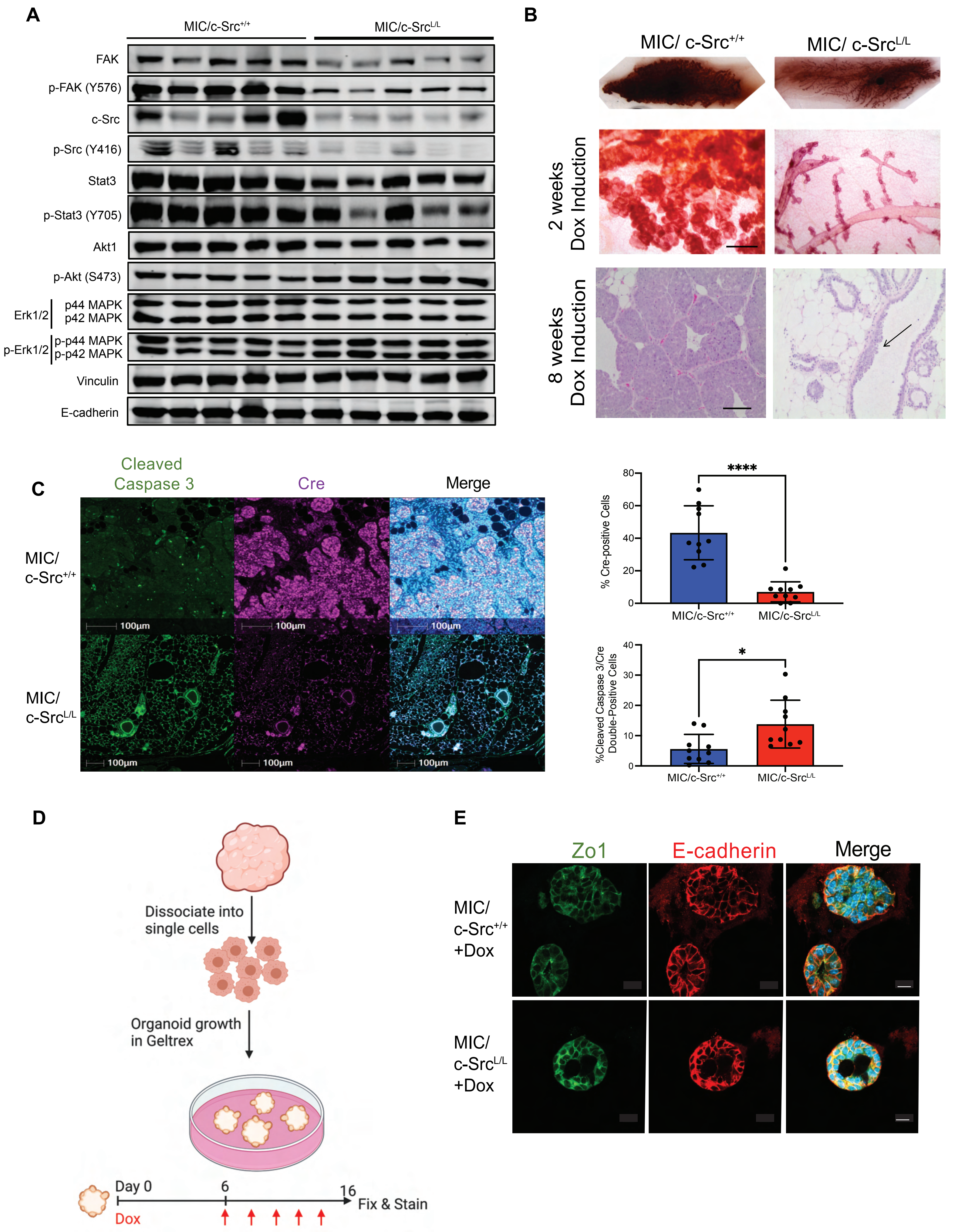
A
MIC/c-Src+/+
MIC/c-SrcL/L
FAK
p-FAK (Y576)
c-Src
p-Src (Y416)
Stat3
p-Stat3 (Y705)
Akt1
p-Akt (S473)
Erk1/2
p44 MAPK
p42 MAPK
p-Erk1/2
p-p44 MAPK
p-p42 MAPK
Vinculin
E-cadherin
B
MIC/ c-Src+/+
MIC/ c-SrcL/L
2 weeks Dox Induction
8 weeks Dox Induction
C
Cleaved Caspase 3
Cre
Merge
MIC/ c-Src+/+
MIC/ c-SrcL/L
100µm
% Cre-positive Cells
****
MIC/c-Src+/+
MIC/c-SrcL/L
%Cleaved Caspase 3/Cre Double-Positive Cells
*
D
Dissociate into single cells
Organoid growth in Geltrex
Day 0
6
16
Fix & Stain
Dox
E
Zo1
E-cadherin
Merge
MIC/ c-Src+/+ +Dox
MIC/ c-SrcL/L +Dox

Supplemental Figure 2: c-Src ablation impairs early tumor progression without affecting canonical PyV mT signaling.
(A) Lysates from 4-week doxycycline induced MIC/c-Src$^{+/+}$ and MIC/c-Src$^{L/L}$ mice were immunoblotted with the indicated antibodies. **(B)** Representative histological images of mammary glands from MIC/c-Src$^{+/+}$ and MIC/c-Src$^{L/L}$ mice. Top 4 images - hematoxylin-stained, wholemounted mammary glands from 10-week old mice induced with doxycycline for 2 weeks Scale bar represents 500 µm. Bottom 2 images - H&E staining of mammary glands following 8 weeks of doxycycline induction. Scale bar represents 100 µm. Images are representative of 10 independent mammary glands per genotype. **(C)** Left panel – Mammary gland sections from mice as in (A) were immunofluorescently stained with the indicated antibodies and DAPI. Representative images from 10 mice per genotype. Scale bar represents 100 µm. Right panels – quantification of Cre-positive and Cleaved Caspase 3/Cre double-positive cells (n = 10 per genotype, minimum of 10,000 total nuclei analyzed. *$p <$ 0.05, ****$p < 0.0001$; unpaired, two-tailed Student's t-test. **(D)** Schematic illustrating the 3D organotypic culture model. **(E)** Organoids were immunofluorescently stained with the indicated antibodies. Scale bar represents 10 µm. Images are representative of organoids from three independent mice per genotype.

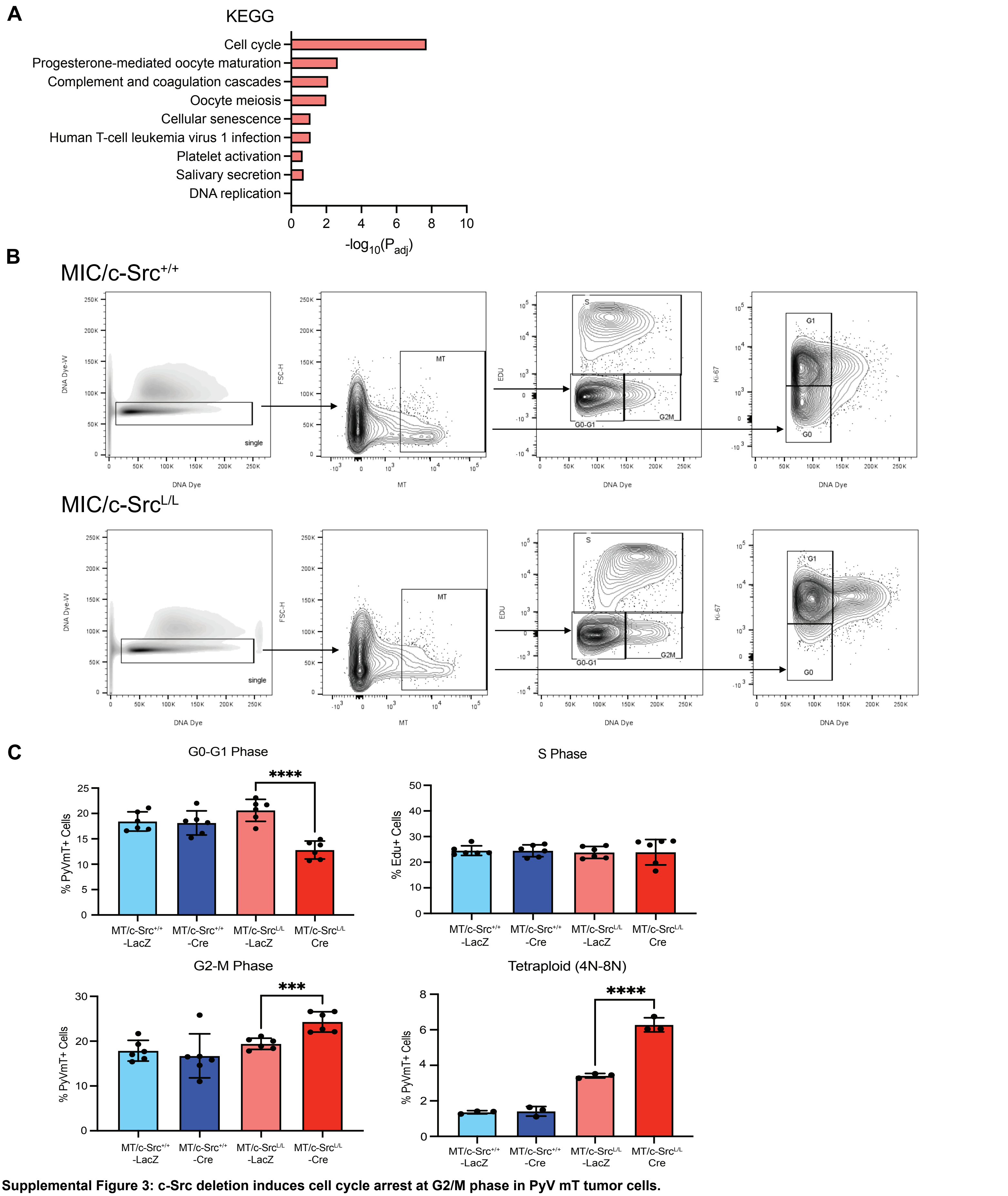

Supplemental Figure 3: c-Src deletion induces cell cycle arrest at G2/M phase in PyV mT tumor cells.
(A) KEGG pathway analysis of transcriptomic data from MIC/c-Src$^{+/+}$ and MIC/c-Src$^{L/L}$ organoids. **(B)** Gating strategy for flow cytometric analysis assessing the cell cycle in mammary epithelial cells from MIC/c-Src$^{+/+}$ and MIC/c-Src$^{L/L}$ mice. **(C)** Flow cytometric analysis of MT/c-Src$^{+/+}$ and MT/c-Src$^{L/L}$ cells, transduced *in vitro* with adenoviruses bearing Cre recombinase or LacZ. Quantitative analysis of EdU incorporation and cell cycle stage was performed on at least 250,000 cells per sample (n = 2 cell lines per genotype in triplicate). (***p < 0.001, ****p <0.0001; one-way ANOVA with Tukey's post hoc-test).

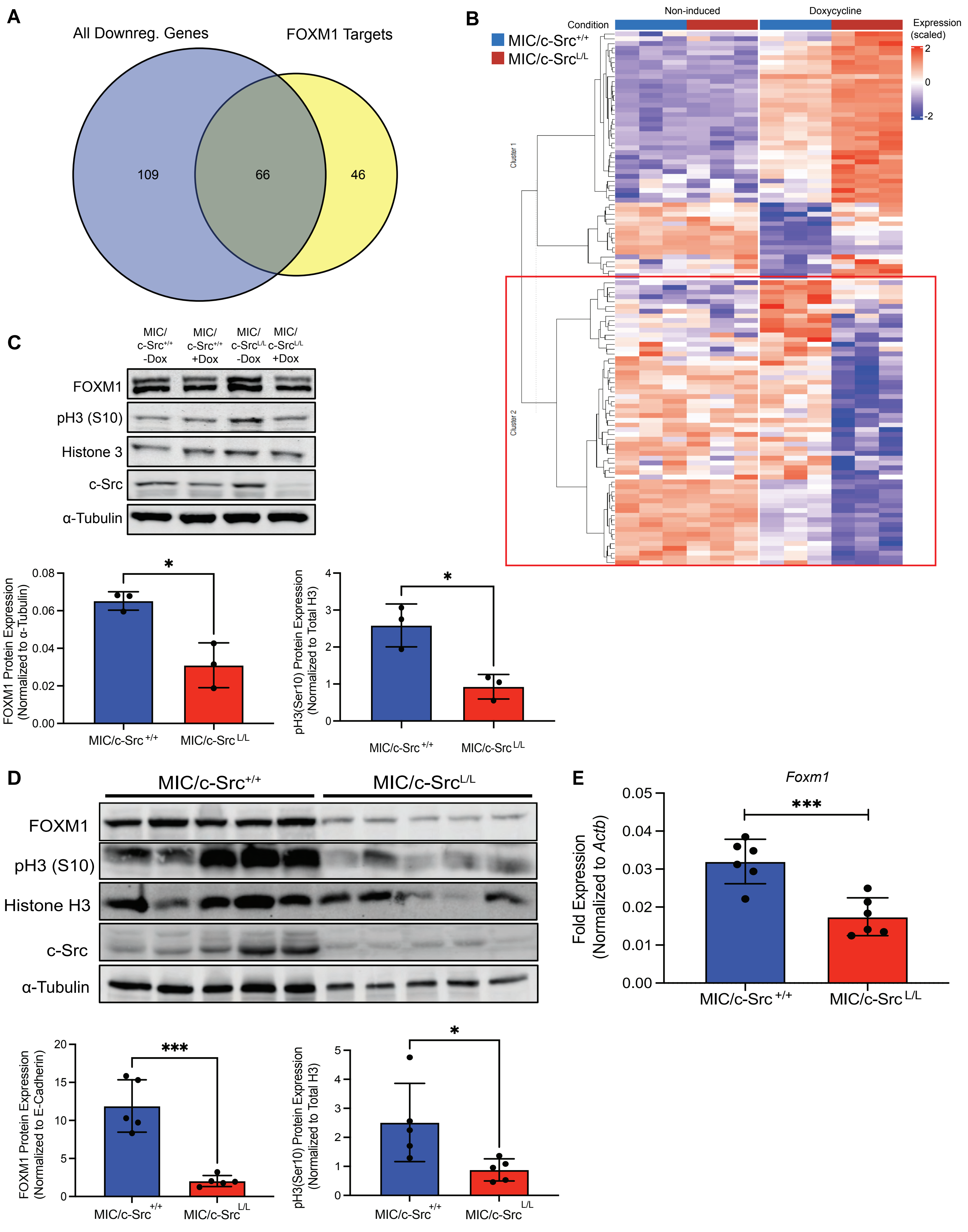

A
All Downreg. Genes
FOXM1 Targets
109
66
46
B
Non-induced
Doxycycline
Condition
MIC/c-Src+/+
MIC/c-SrcL/L
Expression (scaled)
2
0
-2
Cluster 1
Cluster 2
C
MIC/ c-Src+/+ -Dox
MIC/ c-Src+/+ +Dox
MIC/ c-SrcL/L -Dox
MIC/ c-SrcL/L +Dox
FOXM1
pH3 (S10)
Histone 3
c-Src
α-Tubulin
FOXM1 Protein Expression (Normalized to α-Tubulin)
pH3(Ser10) Protein Expression (Normalized to Total H3)
MIC/c-Src +/+
MIC/c-Src L/L
D
MIC/c-Src+/+
MIC/c-SrcL/L
FOXM1
pH3 (S10)
Histone H3
c-Src
α-Tubulin
FOXM1 Protein Expression (Normalized to E-Cadherin)
pH3(Ser10) Protein Expression (Normalized to Total H3)
E
Foxm1
Fold Expression (Normalized to Actb)
MIC/c-Src +/+
MIC/c-Src L/L

Supplemental Figure 4: c-Src ablation blocks cell cycle progression by repressing FOXM1 to block mitotic entry.
(A) Venn diagram illustrating the overlap of transcripts downregulated in doxycycline-induced MIC/c-Src$^{L/L}$ organoids compared to wild-type controls with a FOXM1 target gene signature. **(B)** Hierarchical clustering analysis of FOXM1 target gene expression (up-regulated (red) and down-regulated (blue)) in organoids derived from doxycycline-induced and uninduced MIC/c-Src$^{L/L}$ mammary glands compared to MIC/c-Src$^{+/+}$ controls (n = 3 per genotype). **(C-D)** Doxycycline-induced and uninduced MIC/c-Src$^{L/L}$ and MIC/c-Src$^{+/+}$ organoid (C) and mammary gland (D) lysates were immunoblotted with the indicated antibodies. Representative immunoblots and quantification (fluorescent immunoblotting – LiCOR Odyssey) of FOXM1 and phospho-Histone 3 (Serine 10) expression are shown. 3 independent organoid preparations and five independent mammary glands were analyzed per genotype. *$p < 0.05$, ***$p < 0.001$; unpaired, two-tailed Student's *t*-test. **(E)** *Foxm1* mRNA levels in tumor samples as in (D) were determined using QRT-PCR and normalized to *Actb*. n =5 per genotype, analyzed in triplicate - ***$p < 0.001$; unpaired, two-tailed Student's *t*-test.

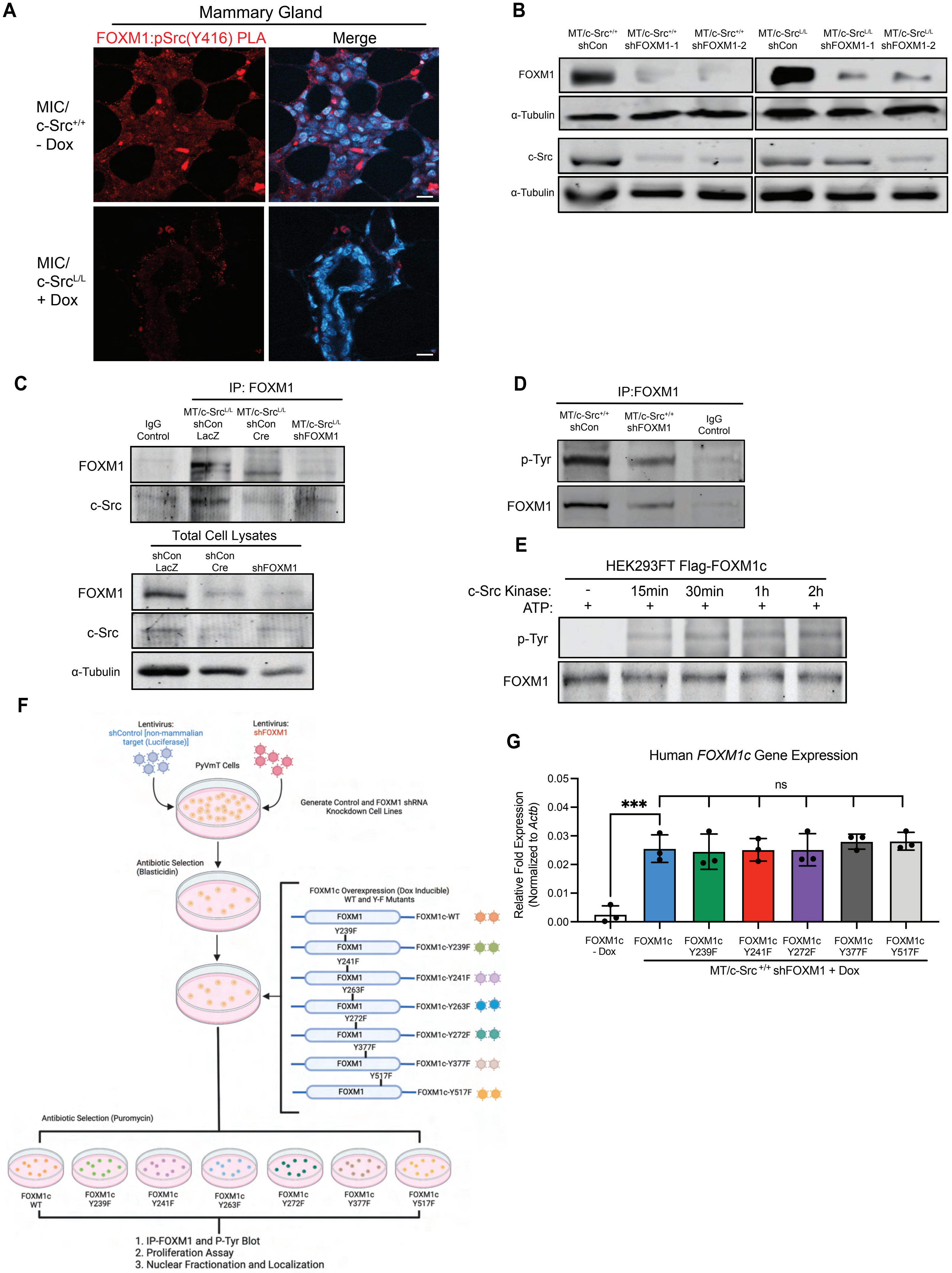
A
Mammary Gland
FOXM1:pSrc(Y416) PLA
Merge
MIC/ c-Src+/+ - Dox
MIC/ c-SrcL/L + Dox
B
MT/c-Src+/+ shCon
MT/c-Src+/+ shFOXM1-1
MT/c-Src+/+ shFOXM1-2
MT/c-SrcL/L shCon
MT/c-SrcL/L shFOXM1-1
MT/c-SrcL/L shFOXM1-2
FOXM1
α-Tubulin
c-Src
α-Tubulin
C
IP: FOXM1
IgG Control
MT/c-SrcL/L shCon LacZ
MT/c-SrcL/L shCon Cre
MT/c-SrcL/L shFOXM1
FOXM1
c-Src
Total Cell Lysates
shCon LacZ
shCon Cre
shFOXM1
FOXM1
c-Src
α-Tubulin
D
IP:FOXM1
MT/c-Src+/+ shCon
MT/c-Src+/+ shFOXM1
IgG Control
p-Tyr
FOXM1
E
HEK293FT Flag-FOXM1c
c-Src Kinase: - 15min 30min 1h 2h
ATP: + + + + +
p-Tyr
FOXM1
F
Lentivirus: shControl [non-mammalian target (Luciferase)]
Lentivirus: shFOXM1
PyVmT Cells
Generate Control and FOXM1 shRNA Knockdown Cell Lines
Antibiotic Selection (Blasticidin)
FOXM1c Overexpression (Dox Inducible) WT and Y-F Mutants
FOXM1 — FOXM1c-WT
Y239F FOXM1 — FOXM1c-Y239F
Y241F FOXM1 — FOXM1c-Y241F
Y263F FOXM1 — FOXM1c-Y263F
Y272F FOXM1 — FOXM1c-Y272F
Y377F FOXM1 — FOXM1c-Y377F
Y517F FOXM1 — FOXM1c-Y517F
Antibiotic Selection (Puromycin)
FOXM1c WT
FOXM1c Y239F
FOXM1c Y241F
FOXM1c Y263F
FOXM1c Y272F
FOXM1c Y377F
FOXM1c Y517F
1. IP-FOXM1 and P-Tyr Blot
2. Proliferation Assay
3. Nuclear Fractionation and Localization
G
Human FOXM1c Gene Expression
Relative Fold Expression (Normalized to Actb)
0.05
0.04
0.03
0.02
0.01
0.00
***
ns
FOXM1c - Dox
FOXM1c
FOXM1c Y239F
FOXM1c Y241F
FOXM1c Y272F
FOXM1c Y377F
FOXM1c Y517F
MT/c-Src+/+ shFOXM1 + Dox

Supplemental Figure 5: FOXM1 interacts with and is phosphorylated by c-Src.
(A) Association between p-SFK (Y416) (Active Src family kinases) and FOXM1 was assessed *in vivo* in doxycycline-induced MIC/c-Src$^{+/+}$ and MIC/c-Src$^{L/L}$ mammary glands using a proximity ligation assay (PLA). Images representative of 5 mice per genotype. Scale bar represents 10 µm. **(B)** PyV mT cells stably expressing shRNAs against Foxm1 or controls were immunoblotted with the indicated antibodies **(C-D)** MT/c-Src$^{L/L}$ cells were transduced with adenoviruses as shown. Foxm1 immunoprecipitates and total cell lysates were immunoblotted with the indicated antibodies. **(E)** FLAG-tagged FOXM1c was expressed in HEK293FT cells, immunoprecipitated, and incubated with purified activated c-Src in an *in vitro* kinase assay. Assays were immunoblotted with the indicated antibodies. **(F)** Schematic illustration of the FOXM1 rescue experimental system involving stable silencing of endogenous Foxm1 and expression of wild-type (WT) and tyrosine-to-phenylalanine (Y-F) mutant forms of FOXM1c. **(G)** Doxycycline-inducible expression of FOXM1c constructs in PyV mT cells stably expressing shRNA against endogenous Foxm1 was determined using QRT-PCR.

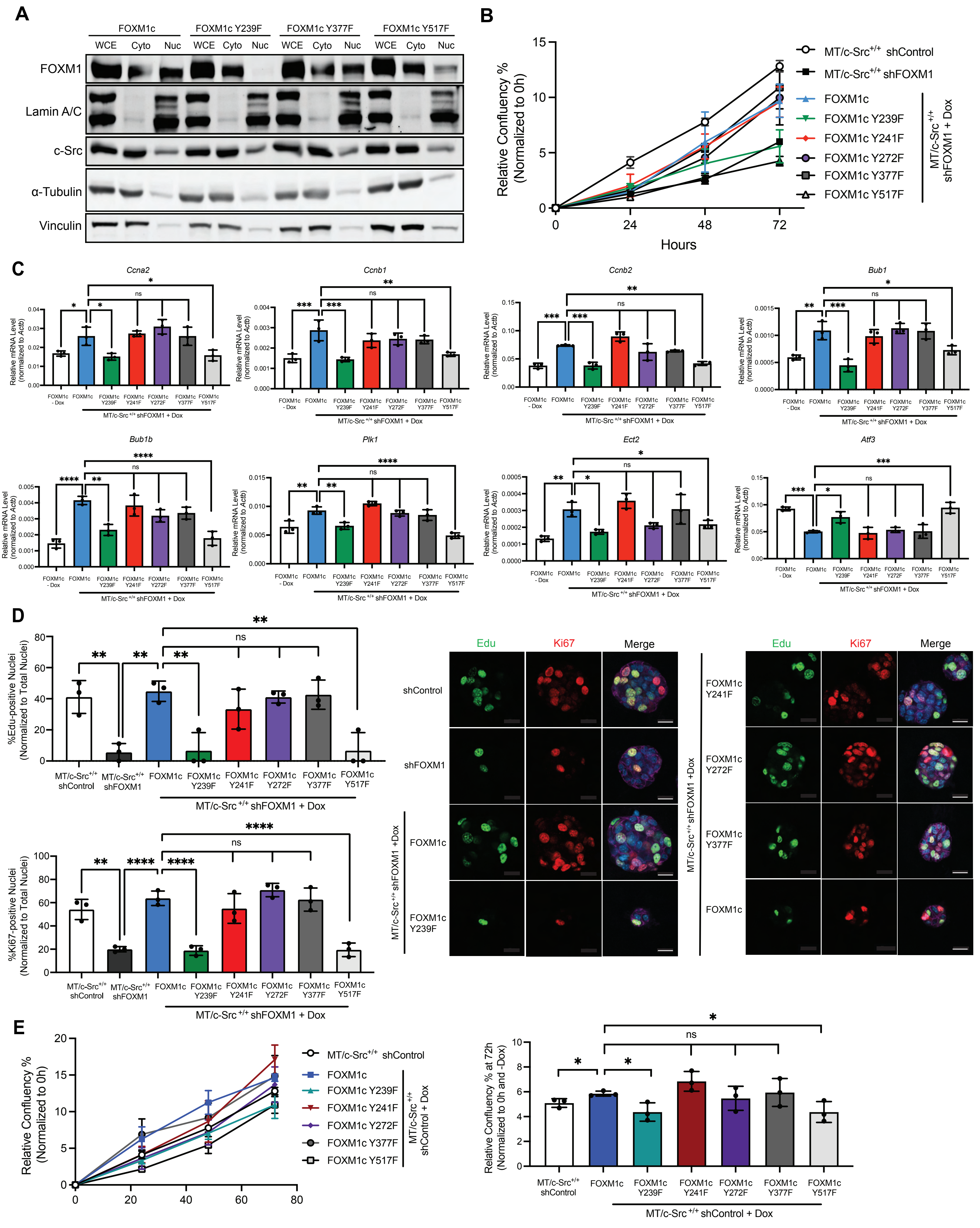

A
FOXM1c
FOXM1c Y239F
FOXM1c Y377F
FOXM1c Y517F
WCE
Cyto
Nuc
FOXM1
Lamin A/C
c-Src
α-Tubulin
Vinculin
B
Relative Confluency % (Normalized to 0h)
Hours
MT/c-Src+/+ shControl
MT/c-Src+/+ shFOXM1
FOXM1c
FOXM1c Y239F
FOXM1c Y241F
FOXM1c Y272F
FOXM1c Y377F
FOXM1c Y517F
MT/c-Src+/+ shFOXM1 + Dox
C
Ccna2
Ccnb1
Ccnb2
Bub1
Bub1b
Plk1
Ect2
Atf3
Relative mRNA Level (normalized to Actb)
FOXM1c - Dox
MT/c-Src+/+ shFOXM1 + Dox
D
%Edu-positive Nuclei (Normalized to Total Nuclei)
%Ki67-positive Nuclei (Normalized to Total Nuclei)
MT/c-Src+/+ shControl
MT/c-Src+/+ shFOXM1
Edu
Ki67
Merge
shControl
shFOXM1
MT/c-Src+/+ shFOXM1 +Dox
E
Relative Confluency % (Normalized to 0h)
Relative Confluency % at 72h (Normalized to 0h and -Dox)
MT/c-Src+/+ shControl + Dox

Supplemental Figure 6: c-Src-dependent tyrosine phosphorylation is required for FOXM1 nuclear localization and function.
(A) Cytoplasmic (cyto) and nuclear (Nuc) fractions were prepared from MT/c-Src$^{+/+}$ cells expressing FOXM1c WT and Y-F mutant constructs and immunoblotted with the indicated antibodies. WCE – whole cell extract. **(B)** Growth curves from the proliferation assay shown in Figure 5F. **(C)** FOXM1 target gene expression was determined by QRT-PCR in cells as in (B) (n = 2 cell lines in triplicate – *$p < 0.05$, **$p < 0.01$, ***$p < 0.001$, ****$p < 0.0001$; one-way ANOVA with Tukey's post hoc-test). **(D)** Cells as in (B-C) were grown in 3D conditions as tumor spheroids and immunostained with the indicated antibodies and DAPI. Left panels – quantification of staining relative to total nuclei (DAPI). Right panels – representative images. **(E)** MT/c-Src$^{+/+}$ cells stably expressing a non-targeting shRNA (shControl) were transduced with the panel of FOXM1c constructs as in (A-D). Proliferation was assessed using an imaging-based assay to monitor cell confluency in real time. Left panel – growth curves. Right panel – endpoint analysis of cell growth at 72h post-doxycycline treatment. n = 2 cell lines in triplicate - *$p < 0.05$; one-way ANOVA with Tukey's post hoc-test.

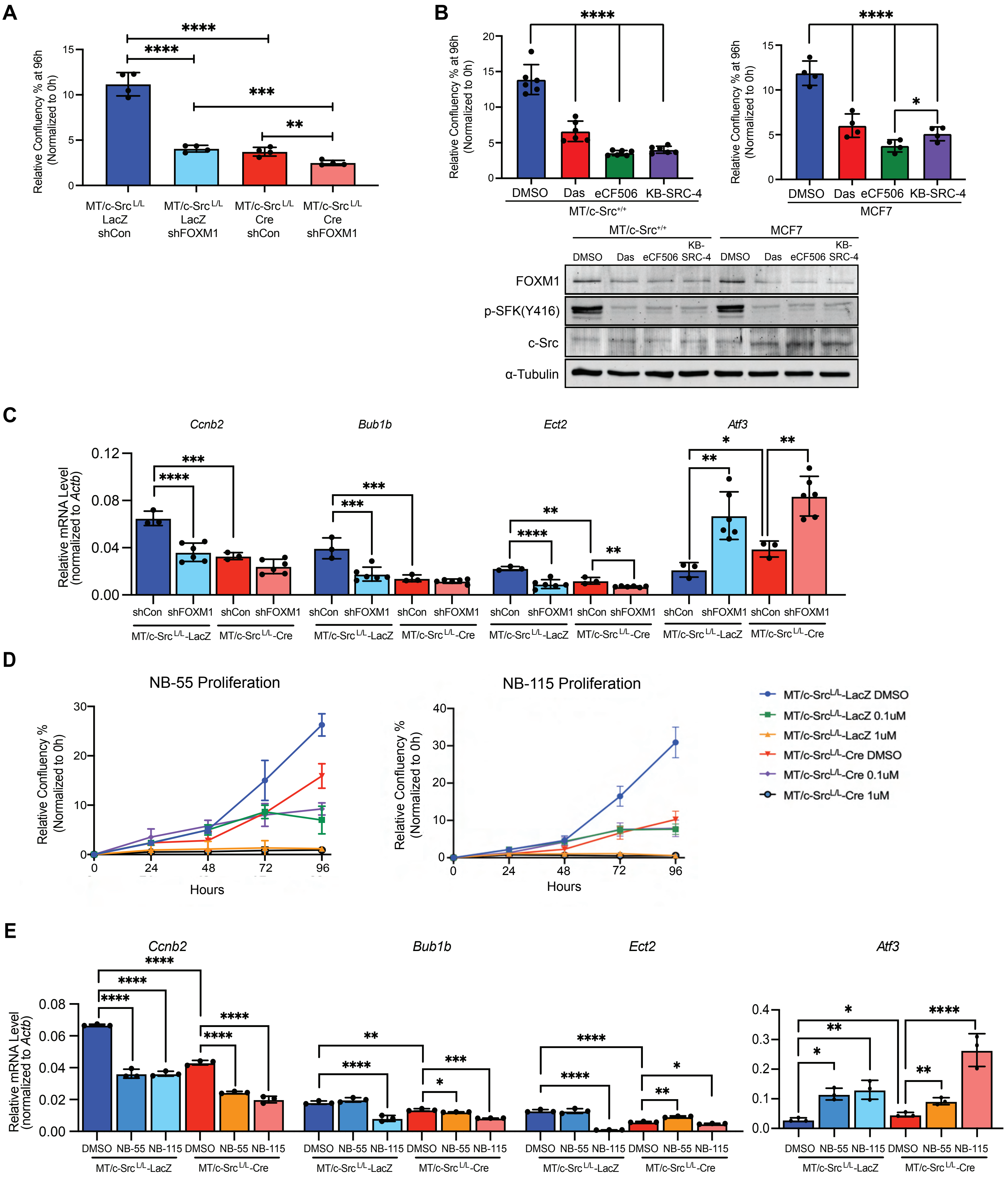
A
Relative Confluency % at 96h
(Normalized to 0h)
****
****
***
**
MT/c-Src L/L
LacZ
shCon
MT/c-Src L/L
LacZ
shFOXM1
MT/c-Src L/L
Cre
shCon
MT/c-Src L/L
Cre
shFOXM1
B
Relative Confluency % at 96h
(Normalized to 0h)
****
*
DMSO
Das
eCF506
KB-SRC-4
MT/c-Src+/+
MCF7
KB-
SRC-4
FOXM1
p-SFK(Y416)
c-Src
α-Tubulin
C
Ccnb2
Bub1b
Ect2
Atf3
Relative mRNA Level
(normalized to Actb)
shCon
shFOXM1
MT/c-Src L/L-LacZ
MT/c-Src L/L-Cre
D
NB-55 Proliferation
NB-115 Proliferation
Relative Confluency %
(Normalized to 0h)
Hours
MT/c-Src L/L-LacZ DMSO
MT/c-Src L/L-LacZ 0.1uM
MT/c-Src L/L-LacZ 1uM
MT/c-Src L/L-Cre DMSO
MT/c-Src L/L-Cre 0.1uM
MT/c-Src L/L-Cre 1uM
E
Ccnb2
Bub1b
Ect2
Atf3
Relative mRNA Level
(normalized to Actb)
DMSO
NB-55
NB-115
MT/c-Src L/L-LacZ
MT/c-Src L/L-Cre

Supplemental Figure 7: Genetic or pharmacological targeting of c-Src or FOXM1 suppresses proliferation and the expression of key cell cycle regulators. **(A)** MT/c-Src$^{L/L}$ cells stably expressing shRNAs against luciferase (control - shCon) or Foxm1 were transduced *in vitro* with adenoviruses bearing Cre recombinase or LacZ. Proliferation was assessed using an imaging-based assay to measure cell confluency in real time. Endpoint analysis after 96h of imaging is shown. Data were normalized to confluency at t=0. **(B)** Wild-type PyV mT cells (MT/c-Src$^{+/+}$) and human MCF7 luminal breast cancer cells were treated with DMSO and the indicated Src family kinase inhibitors, each at 100nM. Das – Dasatinib. Top panels - proliferation was assessed using an imaging-based assay to measure cell confluency in real time. Endpoint analysis after 96h of imaging is shown. Data were normalized to confluency at t=0. *$p < 0.05$, ****$p < 0.0001$; one-way ANOVA with Tukey's post hoc-test. Bottom panel – cell extracts after 48h of treatment were immunoblotted with the indicated antibodies. **(C)** QRT-PCR analysis of FOXM1 target gene mRNA levels in cells as in (A). Mean of experiments performed in two different cell lines in triplicate (*$p < 0.05$, **$p < 0.01$, ***$p < 0.001$, ***$p < 0.0001$; one-way ANOVA with Tukey's post hoc-test). **(D)** MT/c-Src$^{L/L}$ cells were transduced with adenoviruses bearing Cre recombinase or LacZ and treated with FOXM1 inhibitors (NB-55, left panel; NB-115 – right panel) at the indicated concentrations, or DMSO as a control. Proliferation was assessed using an imaging-based assay to measure cell confluency in real time. Growth curves correspond to endpoint data shown in Figure 6D. **(E)** QRT-PCR analysis of FOXM1 target gene expression in cells as in (D). n = 2 cell lines in triplicate - *$p < 0.05$, **$p < 0.01$, ***$p < 0.001$, ****$p < 0.0001$; one-way ANOVA with Tukey's post hoc-test.

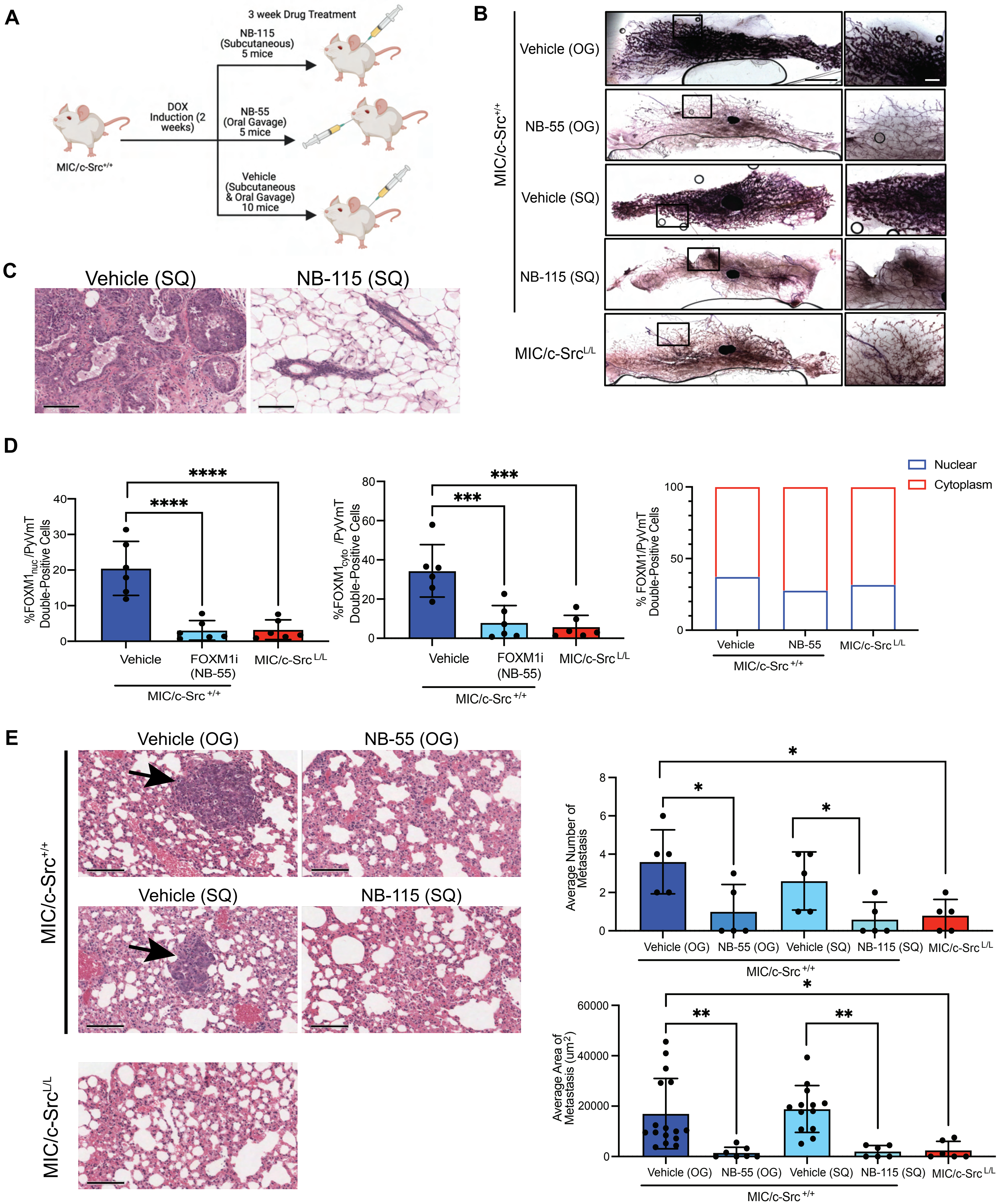

A
3 week Drug Treatment
NB-115
(Subcutaneous)
5 mice
DOX
Induction (2
weeks)
NB-55
(Oral Gavage)
5 mice
Vehicle
(Subcutaneous
& Oral Gavage)
10 mice
MIC/c-Src+/+
B
MIC/c-Src+/+
Vehicle (OG)
NB-55 (OG)
Vehicle (SQ)
NB-115 (SQ)
MIC/c-SrcL/L
C
Vehicle (SQ)
NB-115 (SQ)
D
%FOXM1nuc/PyVmT Double-Positive Cells
****
****
Vehicle
FOXM1i (NB-55)
MIC/c-Src L/L
MIC/c-Src +/+
%FOXM1cyto/PyVmT Double-Positive Cells
***
***
Vehicle
FOXM1i (NB-55)
MIC/c-Src L/L
MIC/c-Src +/+
Nuclear
Cytoplasm
% FOXM1/PyVmT Double-Positive Cells
Vehicle
NB-55
MIC/c-Src L/L
MIC/c-Src +/+
E
Vehicle (OG)
NB-55 (OG)
Vehicle (SQ)
NB-115 (SQ)
MIC/c-Src+/+
MIC/c-SrcL/L
Average Number of Metastasis
*
*
*
Vehicle (OG)
NB-55 (OG)
Vehicle (SQ)
NB-115 (SQ)
MIC/c-Src L/L
MIC/c-Src +/+
Average Area of Metastasis (um2)
*
**
**
Vehicle (OG)
NB-55 (OG)
Vehicle (SQ)
NB-115 (SQ)
MIC/c-Src L/L
MIC/c-Src +/+

Supplemental Figure 8: FOXM1 inhibitors block tumor progression and metastasis *in vivo.*

(A) Schematic of the preclinical model. **(B)** Representative haematoxylin-stained images of wholemounted mammary glands from MIC/c-Src$^{+/+}$ mice induced with doxycycline for 2 weeks and treated with Vehicle or FOXM1 inhibitors or 3 weeks, or MIC/c-Src$^{L/L}$ mice induced with doxycycline for 5-weeks as a comparison (n = 5 per treatment group). Scale bars represent 5000 and 1000 μm, respectively. **(C)** H&E staining of mammary gland samples from vehicle and NB-115-treated mice as in A-B. Scale bars indicate 100 μm. **(D)** Quantification of Foxm1 immunofluorescent staining shown in Fig. 7B, using digital pathology analysis to detect FOXM1 (nuclear)/PyVmT and FOXM1 (cytoplasmic)/PyVmT double-positive cells in mammary glands from MIC/c-Src$^{+/+}$ mice treated as in (A) and MIC/c-Src$^{L/L}$ mice. Stacked column graph represents the ratio of PyVmT-positive cells with FOXM1 nuclear and cytoplasmic staining. n = 6 per treatment group – ***$p < 0.001$, ****$p < 0.0001$; one-way ANOVA with Tukey's post hoc-test. **(E)** Left panel – representative H&E images of lungs from MIC/c-Src$^{+/+}$ mice induced with doxycycline and treated with vehicle or FOXM1 inhibitor (NB-55, NB-115), with lungs from MIC/c-Src$^{L/L}$ mice induced with doxycycline as a comparison. Images are representative of five independent lung sections of each treatment group (n = 5 mice). Scale bar represents 100 μm. Right panel – Average number and total area of lung metastases n = 5 per treatment group, *$p < 0.05$, **$p < 0.01$; one-way ANOVA with Tukey's post hoc-test.

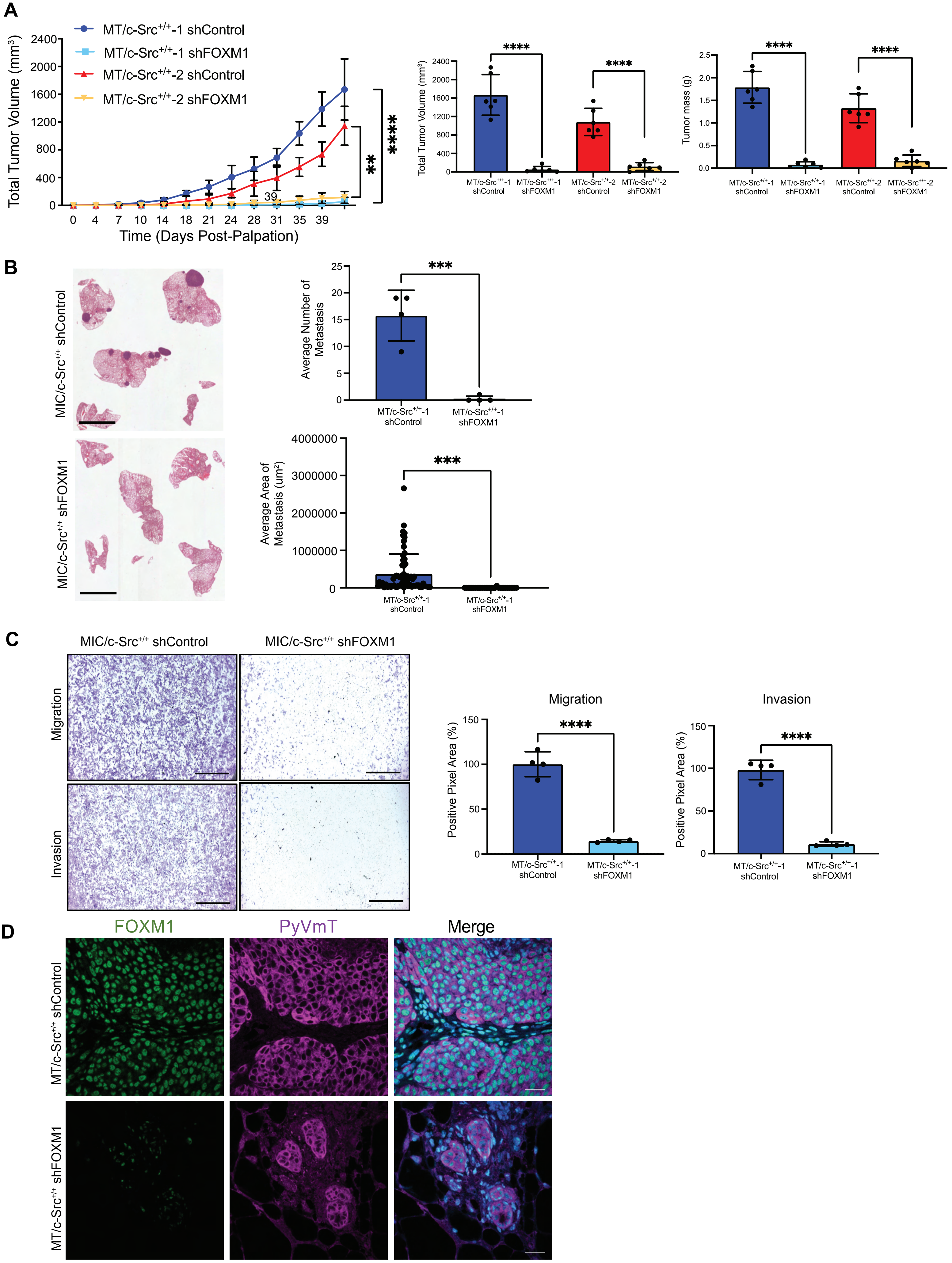
A
MT/c-Src+/+-1 shControl
MT/c-Src+/+-1 shFOXM1
MT/c-Src+/+-2 shControl
MT/c-Src+/+-2 shFOXM1
Total Tumor Volume (mm3)
Time (Days Post-Palpation)
0 4 7 10 14 18 21 24 28 31 35 39
39
**
****
Total Tumor Volume (mm3)
Tumor mass (g)
MT/c-Src+/+-1 shControl
MT/c-Src+/+-1 shFOXM1
MT/c-Src+/+-2 shControl
MT/c-Src+/+-2 shFOXM1
B
MIC/c-Src+/+ shControl
MIC/c-Src+/+ shFOXM1
Average Number of Metastasis
***
Average Area of Metastasis (um2)
MT/c-Src+/+-1 shControl
MT/c-Src+/+-1 shFOXM1
C
MIC/c-Src+/+ shControl
MIC/c-Src+/+ shFOXM1
Migration
Invasion
Positive Pixel Area (%)
****
D
FOXM1
PyVmT
Merge
MT/c-Src+/+ shControl
MT/c-Src+/+ shFOXM1

Supplemental Figure 9: Genetic targeting of Foxm1 suppresses tumor growth and metastasis.
(A) Two independent MT/c-Src$^{+/+}$ cell lines (MT/c-Src$^{+/+}$-1 and MT/c-Src$^{+/+}$-2) stably expressing control or Foxm1-targeting shRNAs were injected orthotopically into the mammary fat pads of immunocompromised mice. Left panel – tumor burden was determined by weekly caliper measurements. **p <0.01, ****p < 0.0001; one-way ANOVA with Tukey's post hoc-test. Right panel – Tumor volume and tumor mass at endpoint. n = 6 mice per treatment group, ****p < 0.0001; one-way ANOVA with Tukey's post hoc-test. **(B)** Left panels - Left panel – representative H&E images of lungs from mice as in (A). Right panels – average number and total area of lung metastases. n = 6 per treatment group, ***p < 0.001; unpaired, two-tailed Student's t-test. **(C)** Left panel – representative images of cell migration and invasion (Boyden chamber) assays. Scale bar represents 1000 µm. Right panels – quantification (positive pixel area) of cell migration and invasion. **(D)** Mammary tumors from the experiment shown in (A) were immunostained with the indicated antibodies. Scale bar indicates 50 µm.

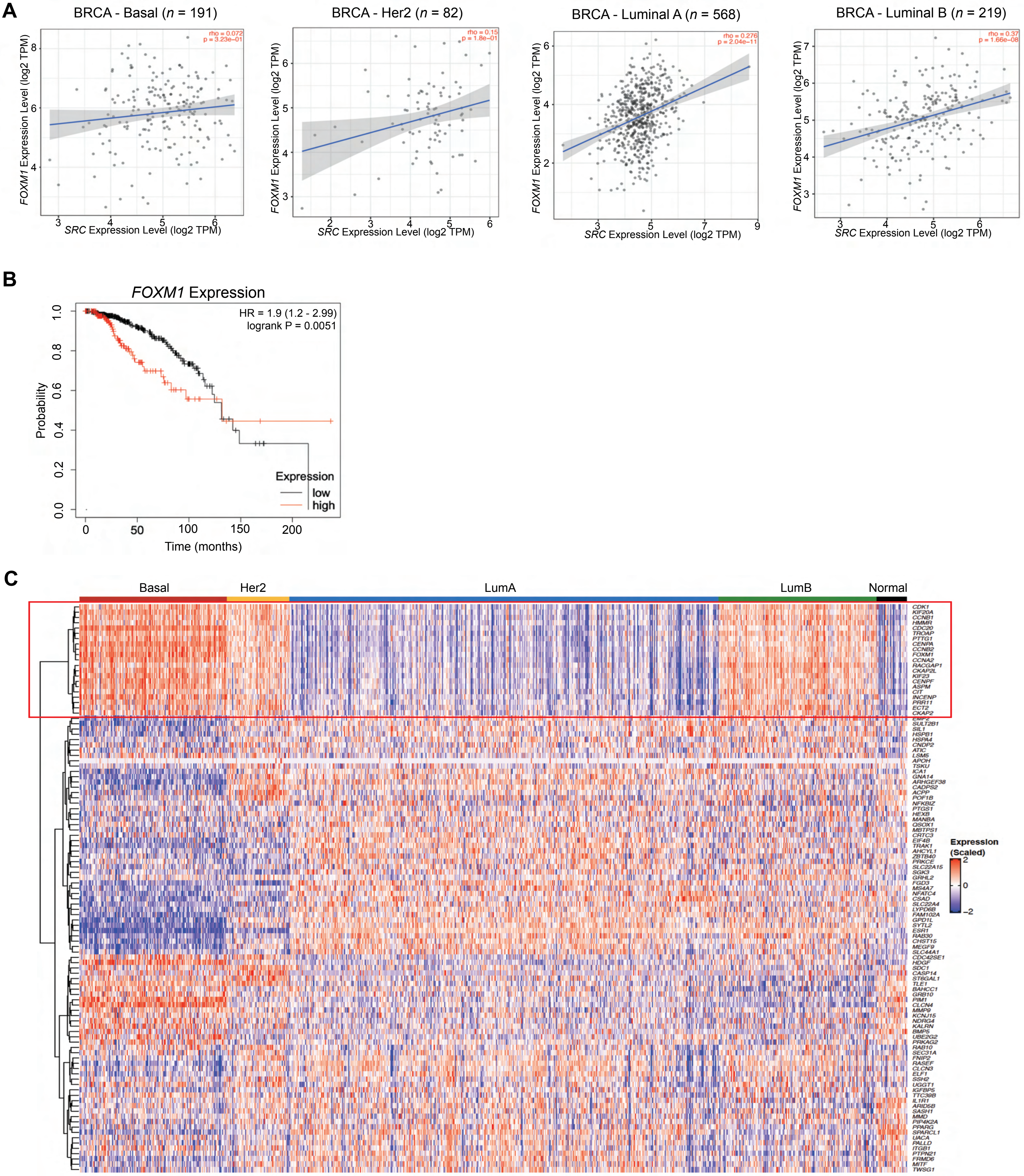

Supplemental Figure 10: FOXM1 expression correlates with SRC expression and poor outcome in Luminal B-like human breast cancer.
(A) Spearman's correlation analysis of FOXM1 and SRC expression levels in TCGA cohorts of Basal (n = 191), HER2 (n = 82), Luminal A (n = 568), and Luminal B (n = 219) patients. **(B)** Kaplan-Meier overall survival curve of Luminal B tumors (n = 627) with high or low FOXM1 expression (log rank p = 0.0051). **(C)** Heatmap of differentially expressed FOXM1 targets in patients clustered by breast cancer subtype (up-regulated genes in red and down-regulated genes in blue).

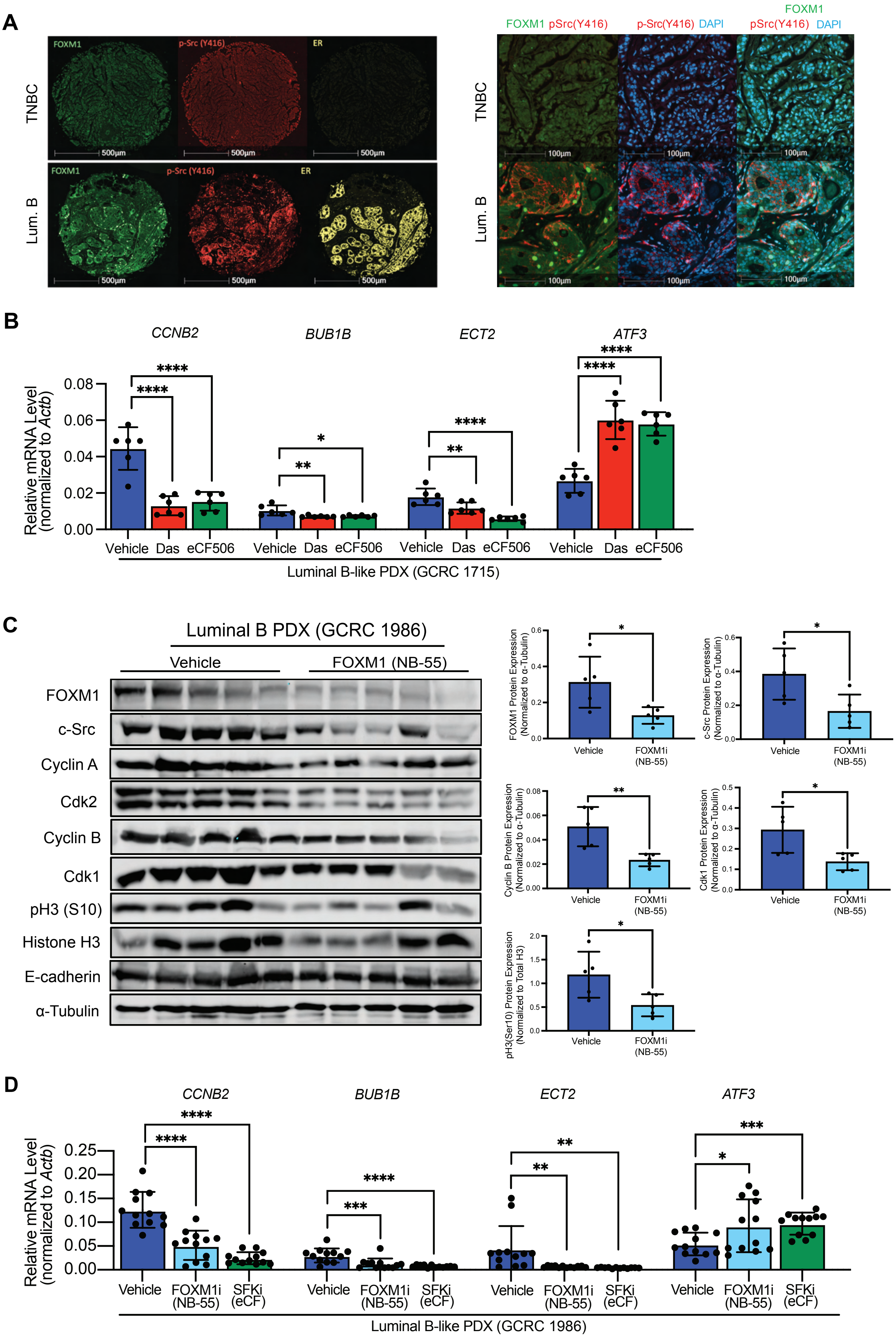
A
FOXM1
p-Src (Y416)
ER
TNBC
Lum. B
500μm
FOXM1 pSrc(Y416)
p-Src(Y416) DAPI
FOXM1 pSrc(Y416) DAPI
100μm
B
CCNB2
BUB1B
ECT2
ATF3
Relative mRNA Level (normalized to Actb)
Vehicle
Das
eCF506
Luminal B-like PDX (GCRC 1715)
C
Luminal B PDX (GCRC 1986)
Vehicle
FOXM1 (NB-55)
FOXM1
c-Src
Cyclin A
Cdk2
Cyclin B
Cdk1
pH3 (S10)
Histone H3
E-cadherin
α-Tubulin
FOXM1 Protein Expression (Normalized to α-Tubulin)
c-Src Protein Expression (Normalized to α-Tubulin)
Cyclin B Protein Expression (Normalized to α-Tubulin)
Cdk1 Protein Expression (Normalized to α-Tubulin)
pH3(Ser10) Protein Expression (Normalized to Total H3)
FOXM1i (NB-55)
D
FOXM1i (NB-55)
SFKi (eCF)
Luminal B-like PDX (GCRC 1986)

Supplemental Figure 11: Targeting coordinated c-Src/FOXM1 activity in PDX models of Luminal B-like breast cancer suppresses cell cycle progression and FOXM1 target gene expression.

(A) Estrogen receptor-negative (ER-) (*n* = 76) and -positive (ER+) (*n* = 84) breast tumor tissue TMA samples stained with the indicated antibodies. Left panel – representative immunofluorescence (IF) images of FOXM1, p-SFK (Y416) and ER staining in TNBC and Luminal B TMA cores. Scale bar represents 500 μm. Right panel – representative image depicting FOXM1 colocalization with p-SFK (Y416) and DAPI (nuclei). Images are representative of quantitative data shown in Figure 10A. **(B)** QRT-PCR analysis of FOXM1 target gene expression in Luminal B-like PDX tumors from mice treated with SFK inhibitors (Dasatinib, eCF506) or vehicle. *n* = 6 per treatment – **p* <0.05, ***p* < 0.0001, *****p* < 0.0001, one-way ANOVA with Dunnett's post hoc-test. **(C)** Left panel - Lysates from Luminal B PDX tumors treated with FOXM1 inhibitor (NB-55) or vehicle (*n* = 5 per treatment) were immunoblotted with the indicated antibodies. Right panel – Quantification (fluorescent immunoblotting – LiCOR Odyssey) (*p < 0.05, **p < 0.01; unpaired, two-tailed Student's *t*-test). **(D)** QRT-PCR analysis of FOXM1 target gene mRNA levels in Luminal B PDX tumors treated with FOXM1 inhibitor (NB-55) or SFK inhibitor (eCF506). *n* = 4 per treatment in triplicate - **p* < 0.05, ***p* < 0.01, ****p* < 0.001, *****p* < 0.0001; one-way ANOVA with Dunnett's post hoc-test.

Supplemental Tables

Supplemental Table 1: Genotyping Primers

Gene	Sequences	Species
MTB Genotyping Forward Primer	5'-ACCGTACTCGTCAATTCCAAGGG-3'	Mouse
MTB Genotyping Reverse Primer	5'-TGCCGCCATTATTACGACAAGC-3'	Mouse
PyVmT Genotyping Forward Primer	5'-GGAAGCAAGTACTTCACAAGGG-3'	Mouse
PyVmT Genotyping Reverse Primer	5'-GGAAAGTCACTAGGAGCAGGG-3'	Mouse
Cre Genotyping Forward Primer	5'-TGCTCTGTCCGTTTGCCG-3'	Mouse
Cre Genotyping Reverse Primer	5'-ACTGTGTCCAGACCAGGC-3'	Mouse
FloxSrc Genotyping Forward Primer	5'-GGTCTTGTCATGGCTCTGTC-3'	Mouse
FloxSrc Genotyping Reverse Primer	5'-CATCTCTGCTCACCTGATAG-3'	Mouse

Supplemental Table 2: Primary and Secondary Antibodies

Antibody	Source	Catalogue #	Dilution
Ki67	Cell Signaling	12202	IF: 1/200
BrdU	Cell Signaling	5292	IF: 1/100
E-Cadherin	BD Transduction	610182	IF: 1/100<br>IB: 1/1000
Zo1	Invitrogen	61-7300	IF: 1/100
phospho-Histone 3 (Serine 10)	Cell Signaling	3377	IF: 1/100<br>IB: 1/1000
Cleaved Caspase 3	Cell Signaling	9661	IF: 1/200
FOXM1	Santa Cruz	sc-376471	IF: 1/50<br>IB: 1/500
Cre Recombinase	Cell Signaling	15036	IF: 1/100
PyVmT	Santa Cruz	53481	IF: 1/100
FOXM1	Cell Signaling	20459	IF: 1/300
phospho-Src Family (Tyr416)	Cell Signaling	2101	IF: 1/200<br>IB: 1:1/1000
Estrogen Receptor α	Santa Cruz	sc-8002	IF: 1/100
HER2/ ErbB2	DAKO	A0485	IF: 1/400
Progesterone Receptor	DAKO	A0098	IF: 1/200
Alexa Fluor 488 Goat anti-Rabbit	Fisher Scientific	A32731	IF: 1/1000
Alexa Fluor 488 Goat anti-Mouse	Fisher Scientific	A32723	IF: 1/1000
Alexa Fluor 555 Goat anti-Rabbit	Fisher Scientific	A32732	IF: 1/1000
Alexa Fluor 555 Goat anti-Mouse	Fisher Scientific	A32727	IF: 1/1000
Alexa Fluor 647 Goat anti-Rabbit	Fisher Scientific	A32733	IF: 1/1000
Alexa Fluor 647 Goat anti-Mouse	Fisher Scientific	A32728	IF: 1/1000
Alexa Fluor 647 Phalloidin	Fisher Scientific	A22287	IF: 1/1000
FOXM1	Proteintech	13147-1-AP	IB: 1:1000
Histone H3	Cell Signaling	14269	IB: 1/1000
c-Src (clone GD-11)	Millipore	05-184	IB: 1/1000

α-Tubulin	Cell Signaling	2144	IB: 1/2000
phospho-Tyrosine-1000 Rabbit mAb	Cell Signaling	8954	IB: 1/1000
phospho-Tyrosine-1000 Mouse mAb	Cell Signaling	9411	IB: 1/1000
Flag-Tag (DYKDDDDK Tag)	Cell Signaling	14793	IB: 1/1000
Cyclin A	Santa Cruz	sc-751	IB: 1/1000
Cdk2	BD Transduction	610145	IB: 1/1000
Cyclin B1	Cell Signaling	4138	IB: 1/1000
phospho-Cdc2 (Tyr15)	Cell Signaling	9111	IB: 1/1000
FOXA1	Abcam	ab23738	IB: 1/1000
FAK	BD Transduction	610088	IB: 1/1000
phospho-FAK (Tyr576)	Millipore	07-157	IB: 1/1000
Stat3	Cell Signaling	9139	IB: 1/1000
phospho-Stat3 (Tyr705)	Cell Signaling	9145	IB: 1/1000
Akt1	Cell Signaling	2938	IB: 1/1000
phospho-Akt1 (Ser473)	Cell Signaling	9271	IB: 1/1000
Erk1/2	Cell Signaling	9102	IB: 1/1000
phospho-Erk1/2 (Thr202/Tyr204)	Cell Signaling	9101	IB: 1/1000
Vinculin	Chemicon	MAB3574	IB: 1/5000
IRDye 800CW Donkey anti-Rabbit	Li-COR Biosciences	925-32213	IB: 1/10000
IRDye 680RD Donkey anti-Mouse	Li-COR Biosciences	926-68073	IB: 1/10000

Supplemental Table 3: Mouse and Human quantitative RT-PCR primer sequences

**Gene**	**Sequences**	**Species**
Foxm1 qPCR Forward Primer	5'-CTGATTCTCAAAAGACGGAGGC-3'	Mouse
Foxm1 qPCR Reverse Primer	5'- TTGATAATCTTGATTCCGGCTGG-3'	Mouse
Ccna2 qPCR Forward Primer	5'-GCCTTCACCATTCATGTGGAT-3'	Mouse
Ccna2 qPCR Reverse Primer	5'-TTGCTGCGGGTAAAGAGACAG-3'	Mouse
Ccnb1 qPCR Forward Primer	5'-AAGGTGCCTGTGTGTGAACC-3'	Mouse
Ccnb1 qPCR Reverse Primer	5'-GTCAGCCCCATCATCTGCG-3'	Mouse
Ccnb2 qPCR Forward Primer	5'-GCCAAGAGCCATGTGACTATC-3'	Mouse
Ccnb2 qPCR Reverse Primer	5'-CAGAGCTGGTACTTTGGTGTTC-3'	Mouse
Bub1 qPCR Forward Primer	5'-AGAATGCTCTGTCAGCTCATCT-3'	Mouse
Bub1 qPCR Reverse Primer	5'-TGTCTTCACTAACCCACTGCT-3'	Mouse
Bub1b qPCR Forward Primer	5'-GAGGCGAGTGAAGCCATGT-3'	Mouse
Bub1b qPCR Reverse Primer	5'-TCCAGAGTAAAAGCGGATTTCAG-3'	Mouse
Plk1 qPCR Forward Primer	5'-CTAGCACACCAACACGTCGTA-3'	Mouse
Plk1 qPCR Reverse Primer	5'-ACCTCCAGATCCTCGTTCAGG-3'	Mouse
Ect2 qPCR Forward Primer	5'-GGCCTTAAAGGAAATGAAAGTGC-3'	Mouse
Ect2 qPCR Reverse Primer	5'-ACCAGGTTCAGCATACTCGTA-3'	Mouse
Atf3 qPCR Forward Primer	5'- GAGGATTTTGCTAACCTGACACC-3'	Mouse
Atf3 qPCR Reverse Primer	5'-TTGACGGTAACTGACTCCAGC-3'	Mouse
Src qPCR Forward Primer	5'-GAACCCGAGAGGGACCTTC-3'	Mouse
Src qPCR Reverse Primer	5'-GAGGCAGTAGGCACCTTTTGT-3'	Mouse
Actb qPCR Forward Primer	5'- TCCATCATGAAGTGTGACGT-3'	Mouse

Actb qPCR Reverse Primer	5'- GAGCAATGATCTTGATCTTCAT-3'	Mouse
FOXM1 qPCR Forward Primer	5'-ACGTCCCCAAGCCAGGCTC-3'	Human
FOXM1 qPCR Reverse Primer	5'-CTACTGTAGCTCAGGAATAA-3'	Human
CCNA2 qPCR Forward Primer	5'-GGATGGTAGTTTTGAGTCACCAC-3'	Human
CCNA2 qPCR Reverse Primer	5'-CACGAGGATAGCTCTCATACTGT-3'	Human
CCNB1 qPCR Forward Primer	5'- AACTTTCGCCTGAGCCTATTTT-3'	Human
CCNB1 qPCR Reverse Primer	5'-TTGGTCTGACTGCTTGCTCTT-3'	Human
CCNB2 qPCR Forward Primer	5'-TTGGCTGGTACAAGTCCACTC-3'	Human
CCNB2 qPCR Reverse Primer	5'-TGGGAACTGGTATAAGCATTGTC-3'	Human
BUB1 qPCR Forward Primer	5'-ACAATCAACGGAGAAAGCATGA-3'	Human
BUB1 qPCR Reverse Primer	5'-CTCCACCACCTGATGCAACT-3'	Human
BUB1B qPCR Forward Primer	5'-TAGGGCGTTTATGCAATGAGC-3'	Human
BUB1B qPCR Reverse Primer	5'-TCCTGAAATATCGCATCTGCTTT-3'	Human
PLK1 qPCR Forward Primer	5'-AAAGAGATCCCGGAGGTCCTA-3'	Human
PLK1 qPCR Reverse Primer	5'-GGCTGCGGTGAATGGATATTTC-3'	Human
ECT2 qPCR Forward Primer	5'-TGTAGTCACGGACTTTCAGGA-3'	Human
ECT2 qPCR Reverse Primer	5'-GTACAATACAACGGGCGACAT-3'	Human
ATF3 qPCR Forward Primer	5'-CCTCTGCGCTGGAATCAGTC-3'	Human
ATF3 qPCR Reverse Primer	5'-TTCTTTCTCGTCGCCTCTTTTT-3'	Human
FOXM1c qPCR Forward Primer	5'-CAATTGCCCGAGCACTTGGAATCA-3'	Human
FOXM1c qPCR Reverse Primer	5'-TCCTCAGCTAGCAGCACCTTG-3'	Human
SRC qPCR Forward Primer	5'-GAGCGGCTCCAGATTGTCAA-3'	Human
SRC qPCR Reverse Primer	5'-CTGGGGATGTAGCCTGTCTGT-3'	Human
Actb qPCR Forward Primer	5'-AGAGCTACGAGCTGCCTGAC-3'	Human
Actb qPCR Reverse Primer	5'-AGCACTGTGTTGGCGTACAG-3'	Human

Supplemental Table 4: Cell Cycle Flow Cytometry Analysis - Antibody and Reagent List

**Antibody/ Reagent**	**Company**	**Catalogue Number**
Anti-PyVmT Antibody Alexa Fluor® 647	Santa Cruz	sc-53481
PE/Cyanine7 anti-mouse Ki-67 Antibody	Biolegend	652426
TruStain FcX™ (anti-mouse CD16/32) Antibody	Biolegend	101320
BD Cytofix Fixation buffer	BD Biosciences	554655
Copper (II) Sulfate Solution 0.1M 50 mL	Aldon Corp SE	470300-896
BD Perm/Wash™ buffer	BD Biosciences	554723
CF®405M Dye azide	Biotium	92092
7-AAD (7-amino-actinomycin D)	Biolegend	420404

Supplemental Table 5: ChIP-qPCR Primer Sequences

**Gene Name**	**Sequences**	**Species**
Src ChIP-qPCR Forward Primer	5'-TTCCAGCCCTGTTTCCCCAACC-3'	Mouse
Src ChIP-qPCR Reverse Primer	5'-GCAGCGCCGCTTTCAGTTGTTC -3'	Mouse
Ccnb1 ChIP-qPCR Forward Primer	5'-ACCGTCTCTGCAACAAAGCTTTCG-3'	Mouse
Ccnb1 ChIP-qPCR Reverse Primer	5'-CCCAGGCGACTCCGTTGGACTT-3'	Mouse

Plk1 ChIP-qPCR Forward Primer	5'-CTCTGTGTGGCCACTCTTTT-3'	Mouse
Plk1 ChIP-qPCR Reverse Primer	5'-CCGTTCAGCCTTCACTTAGG-3'	Mouse
Actb ChIP-qPCR Forward Primer	5'- TCCATCATGAAGTGTGACGT-3'	Mouse
Actb ChIP-qPCR Reverse Primer	5'- GAGCAATGATCTTGATCTTCAT-3'	Mouse

Supplemental Table 6: Site-Directed Mutagenesis Primers for pCW57.1-FOXM1c

**Gene**	**Sequences**
FOXM1c SDM Y239F Forward Primer	5'-GCGGCCACCCTTCTCTTACATGG-3'
FOXM1c SDM Y239F Reverse Primer	5'-TCAGACACAGAGTTCTGCCAG-3'
FOXM1c SDM Y241F Forward Primer	5'-ACCCTACTCTTTCATGGCCATGATAC-3'
FOXM1c SDM Y241F Reverse Primer	5'-GGCCGCTCAGACACAGAG-3'
FOXM1c SDM Y263F Forward Primer	5'-GAAAGACATCTTTACGTGGATTGAG-3'
FOXM1c SDM Y263F Reverse Primer	5'-AAAGTCATGCGCTTCCTC-3'
FOXM1c SDM Y272F Forward Primer	5'-CCACTTTCCCTTCTTTAAGCACATTG-3'
FOXM1c SDM Y272F Reverse Primer	5'-TCCTCAATCCACGTATAG-3'
FOXM1c SDM Y377F Forward Primer	5'-GGTCAGCTCATTCCTGGTACCTATC-3'
FOXM1c SDM Y377F Reverse Primer	5'-CGTGGTAGCAGTGGCTTC-3'
FOXM1c SDM Y517F Forward Primer	5'- CAAGAAGTCCTTCAGTGGGCTTAG-3'
FOXM1c SDM Y517F Reverse Primer	5'-GGTCTTGGGGTGGGAGAT-3'
FOXM1c Sequencing Forward Primer-1	5'-GCACTGACTGCCAAGGGAAA-3'
FOXM1c Sequencing Forward Primer-2	5'-ACTGAGAGGAAGCGCATGAC-3'
FOXM1c Sequencing Forward Primer-3	5'-AGCTGAGGAGGGGATAGCTC-3'
FOXM1c Sequencing Forward Primer-4	5'-AGCTCAGCTACTCCCAGGAA-3'

Supplemental Table 7: Oligos for pLKO.1-Blast Cloning of FOXM1 shRNAs

**Gene**	**Sequences**
shFOXM1-1 (TRCN0000084773) Forward Primer	5'-CCGGGCTCCATAGAAATGTGACCATCTCGAGA TGGTCACATTTCTATGGAGCTTTTTG-3'
shFOXM1-1 (TRCN0000084773) Reverse Primer	5'-AATTCAAAAAGCTCCATAGAAATGTGACCATC TCGAGATGGTCACATTTCTATGGAGC-3'
shFOXM1-2 (TRCN0000084774) Forward Primer	5'-CCGGGCTGGACAACAGCTTAACCAACTCGAGT TGGTTAAGCTGTTGTCCAGCTTTTTG-3'
shFOXM1-2 (TRCN0000084774) Reverse Primer	5'-AATTCAAAAAGCTGGACAACAGCTTAACCAAC TCGAGTTGGTTAAGCTGTTGTCCAGC-3'
shFOXM1-3 (TRCN0000304361) Forward Primer	5'- CCGGACTTCCTATTCAGTCCATTAACTCGAGT TAATGGACTGAATAGGAAGTTTTTG-3'
shFOXM1-3 (TRCN0000304361) Reverse Primer	5'-AATTCAAAAAACTTCCTATTCAGTCCATTAAC TCGAGTTAATGGACTGAATAGGAAGT-3'
LKO.1 5' (Weinberg Lab)	5'-GACTATCATATGCTTACCGT-3'

Full unedited gel for Figure 5B

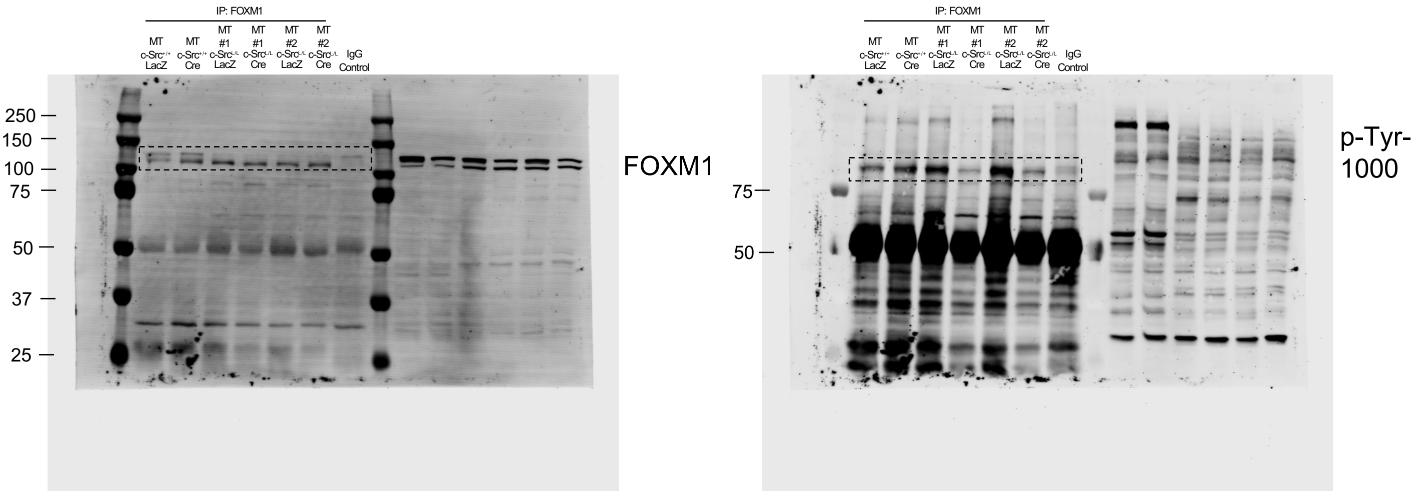

Full unedited gel for Figure 5D

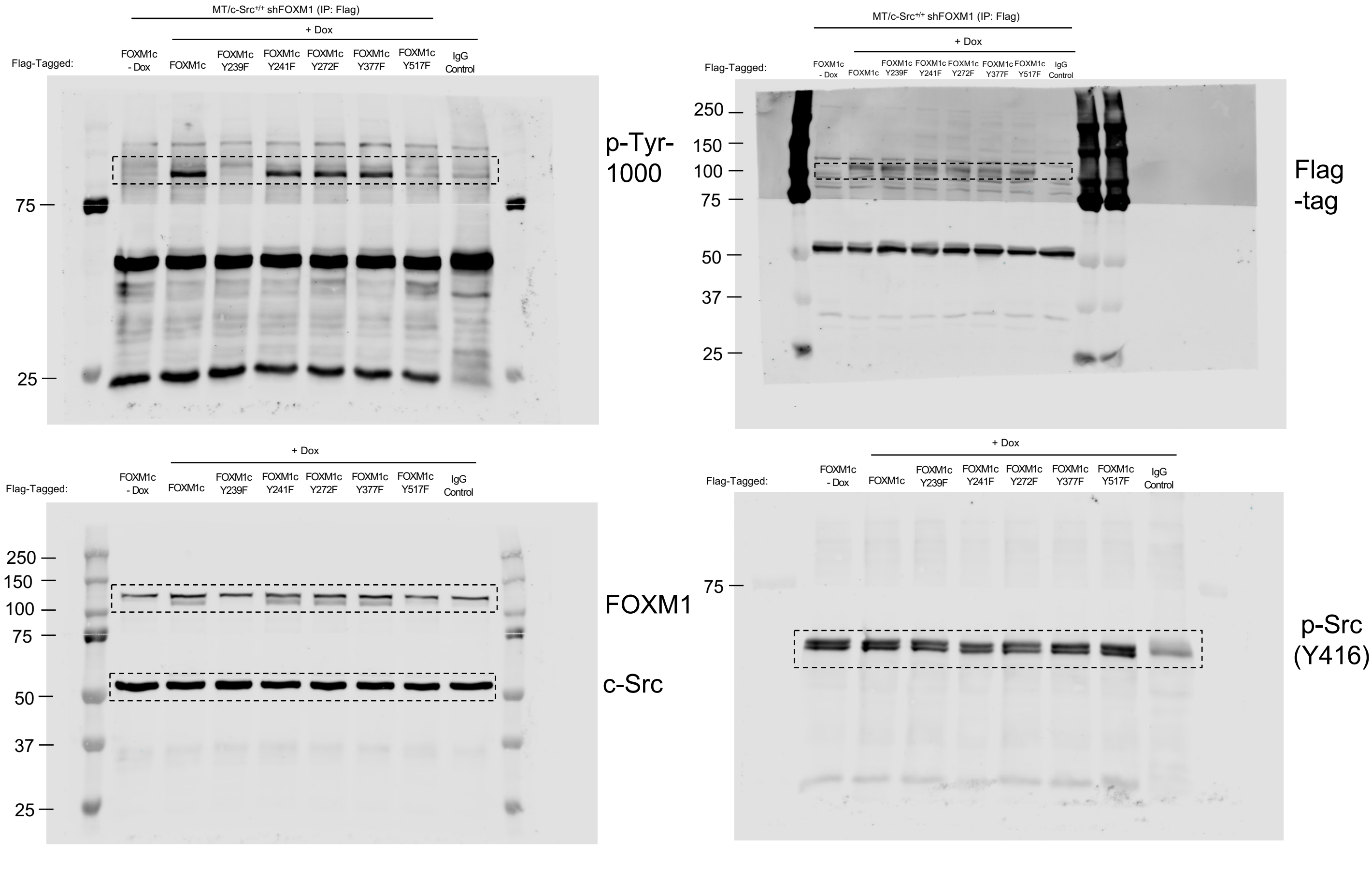

Uncropped western blots for Figure 5B and Figure 5D. Black dotted lines indicate regions that were cropped for the figures.

Full unedited gel for Figure 6E

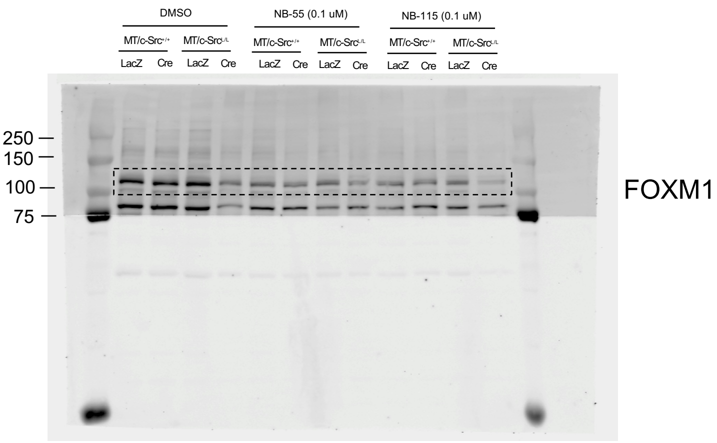

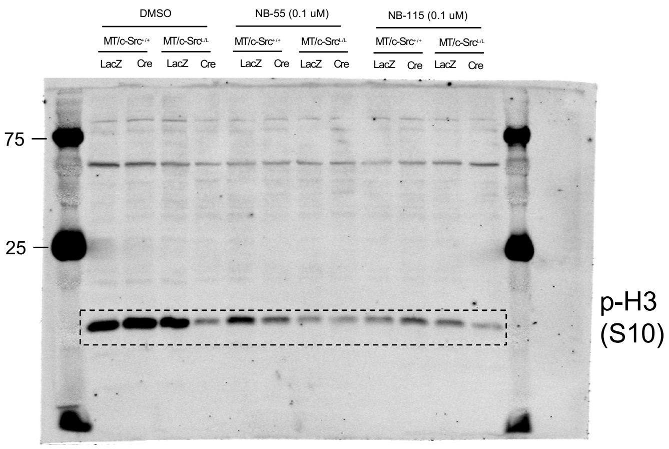

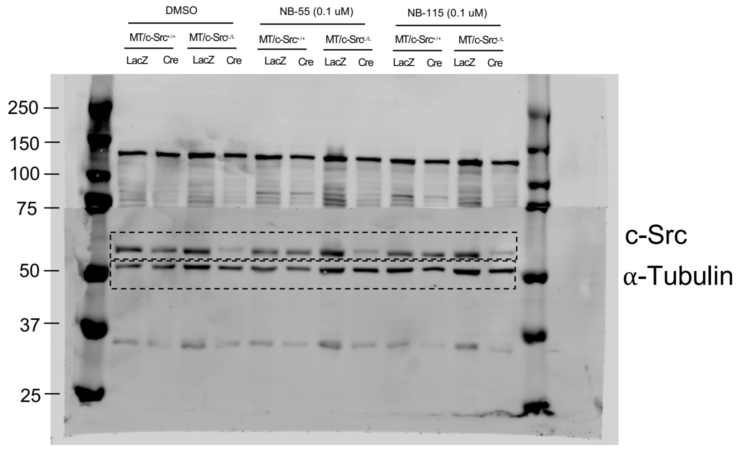

Uncropped western blots for Figure 6E. Black dotted lines indicate regions that were cropped for the figures.

Full unedited gel for Figure 8B

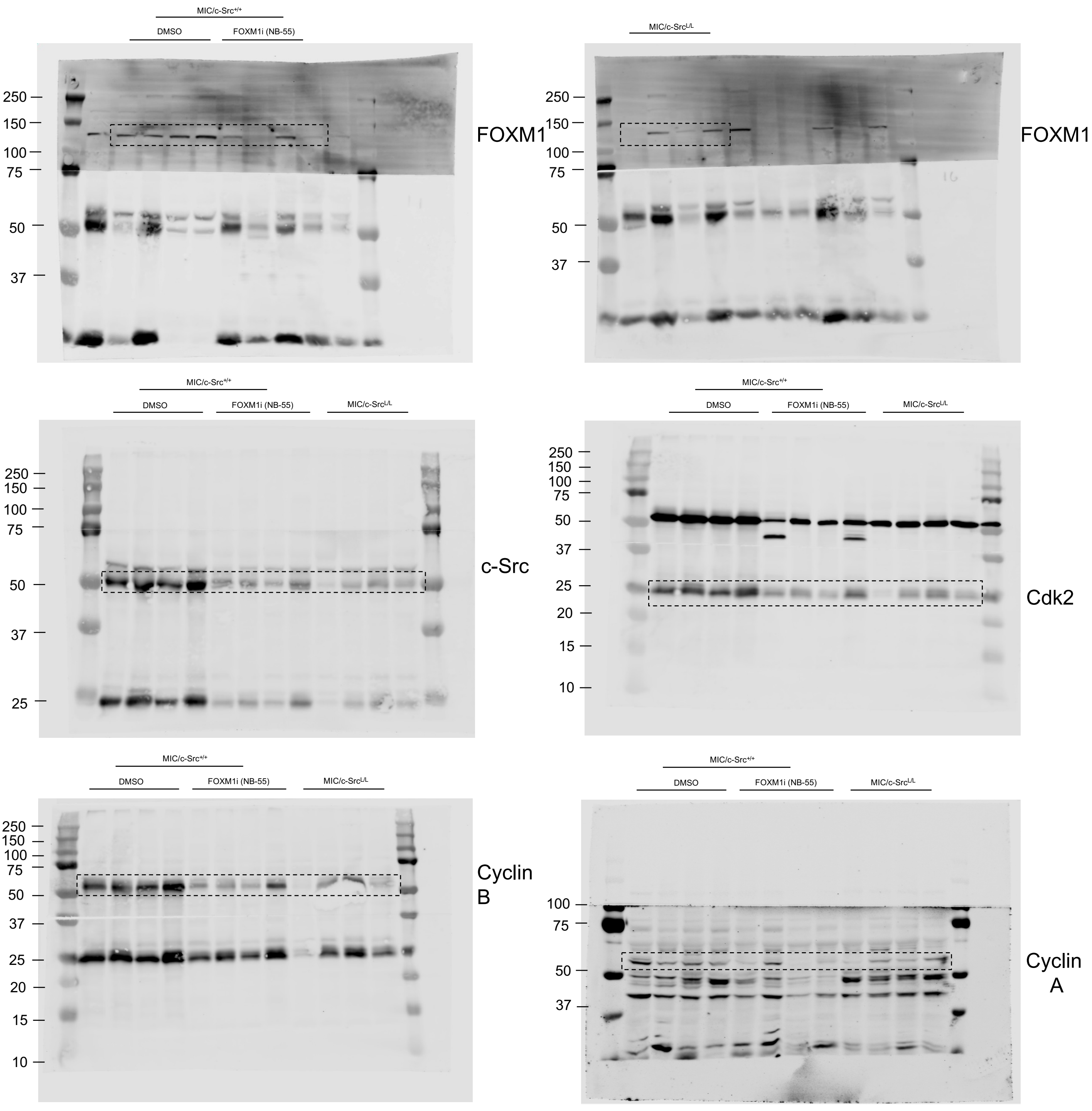

Uncropped western blots for Figure 8B. Black dotted lines indicate regions that were cropped for the figures.

Full unedited gel for Figure 8B (Continued)

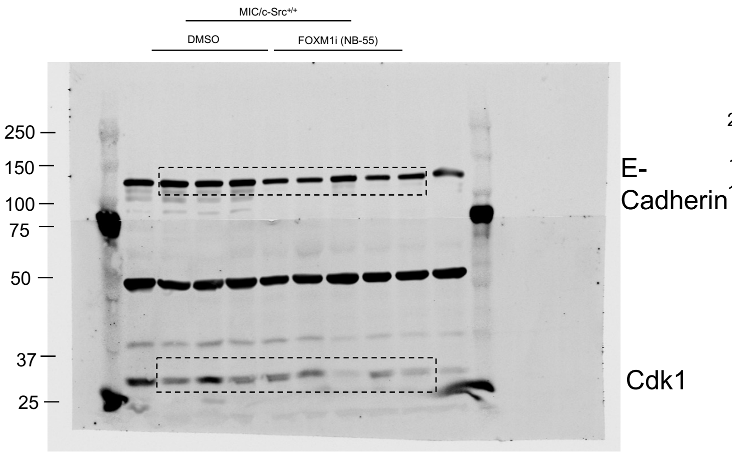

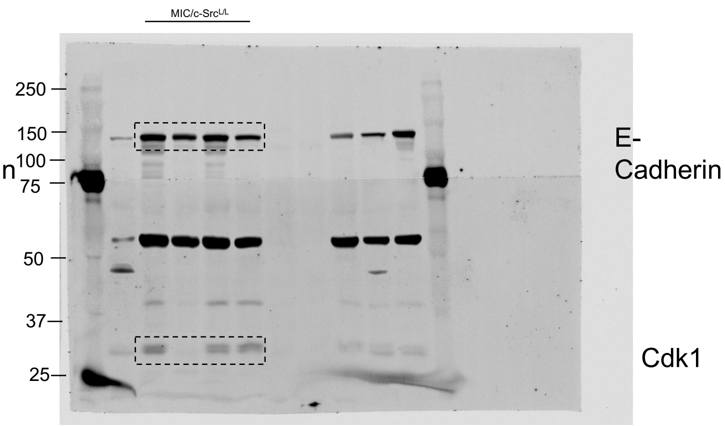

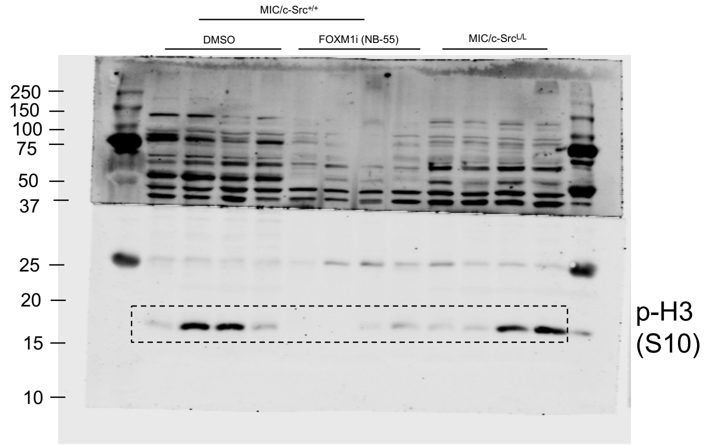

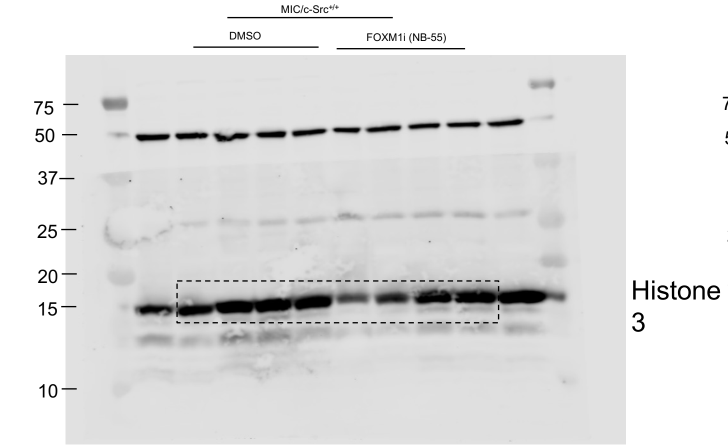

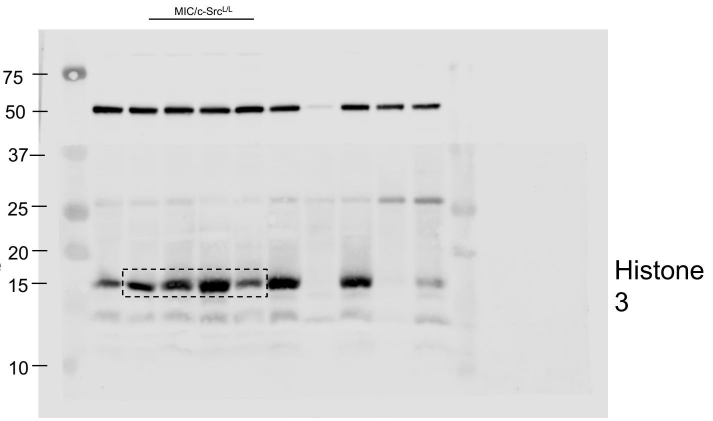

Uncropped western blots for Figure 8B (Continued). Black dotted lines indicate regions that were cropped for the figures.

Full unedited gel for Figure 8B (Continued)

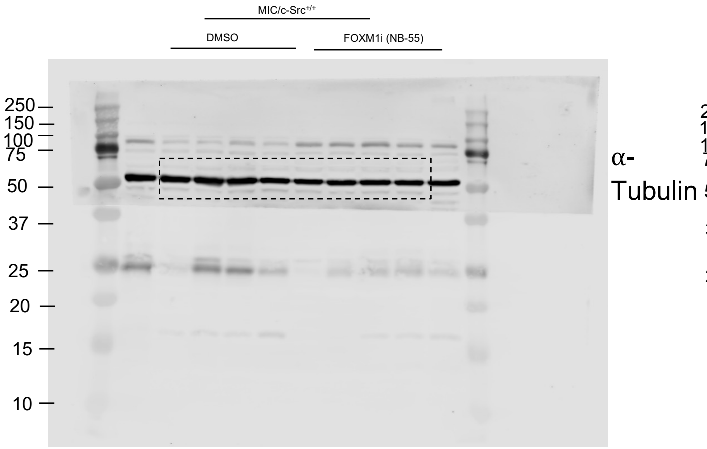

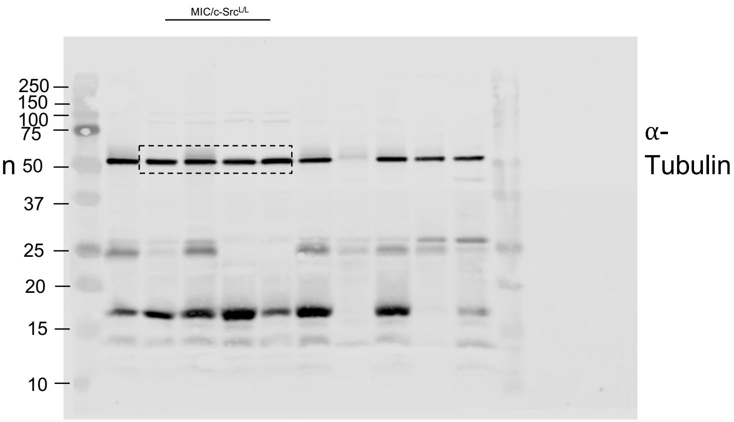

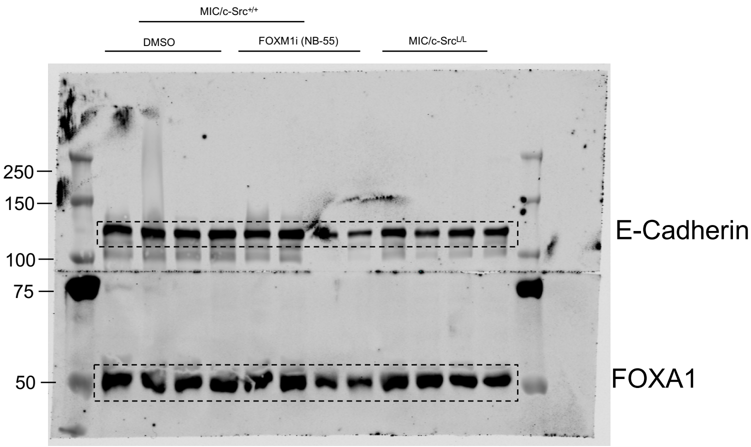

Uncropped western blots for Figure 8B (Continued). Black dotted lines indicate regions that were cropped for the figures.

Full unedited gel for Supplemental Figure 2A

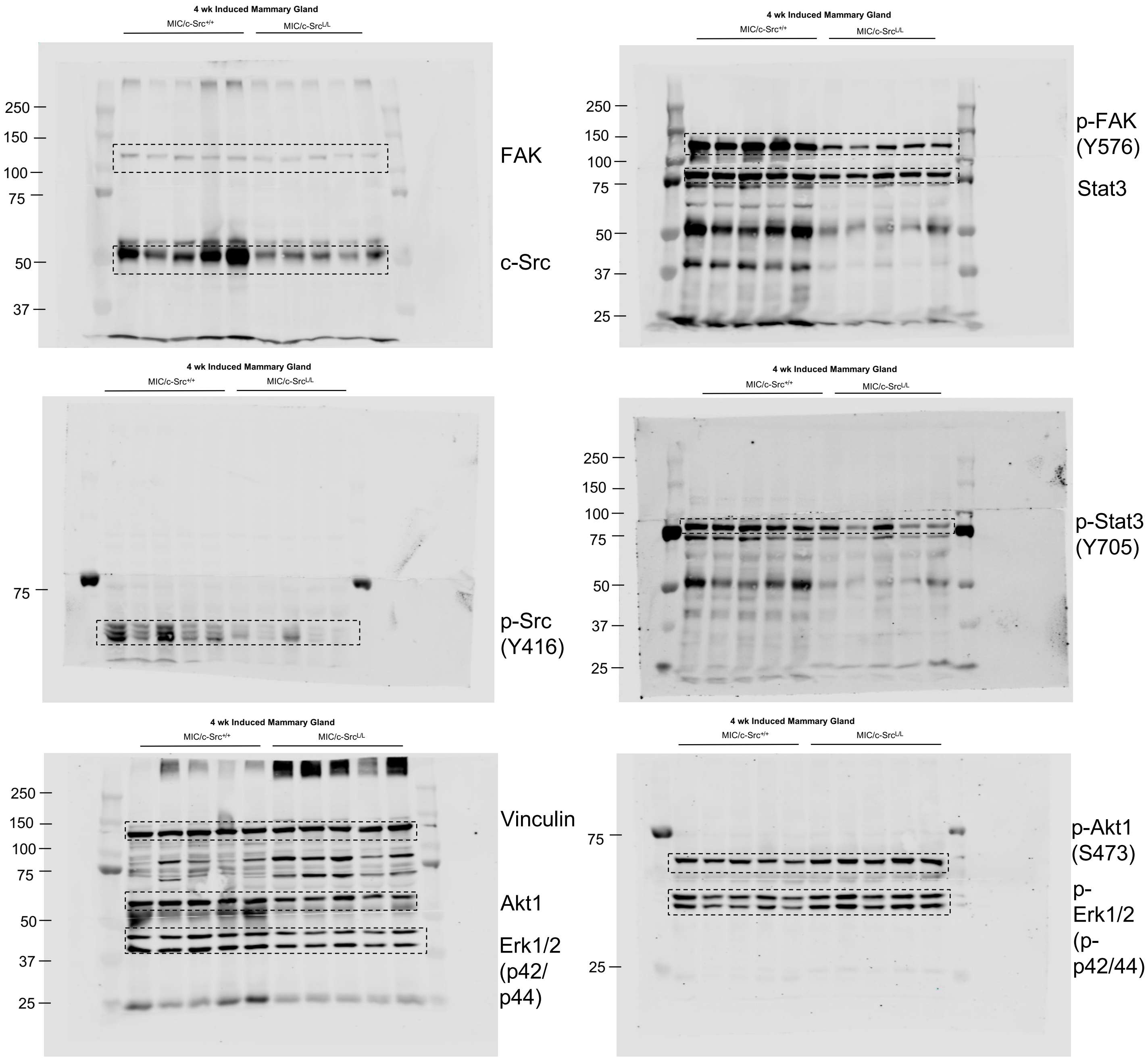

Uncropped western blots for Supplemental Figure 2A. Black dotted lines indicate regions that were cropped for the figures.

Full unedited gel for Supplemental Figure 2A (Continued)

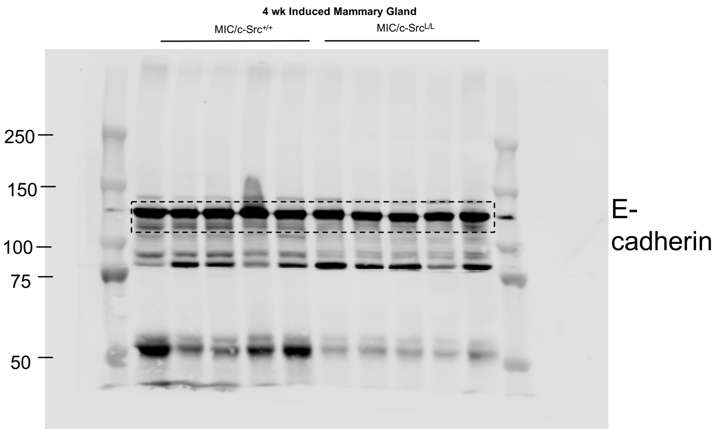

Full unedited gel for Supplemental Figure 4C

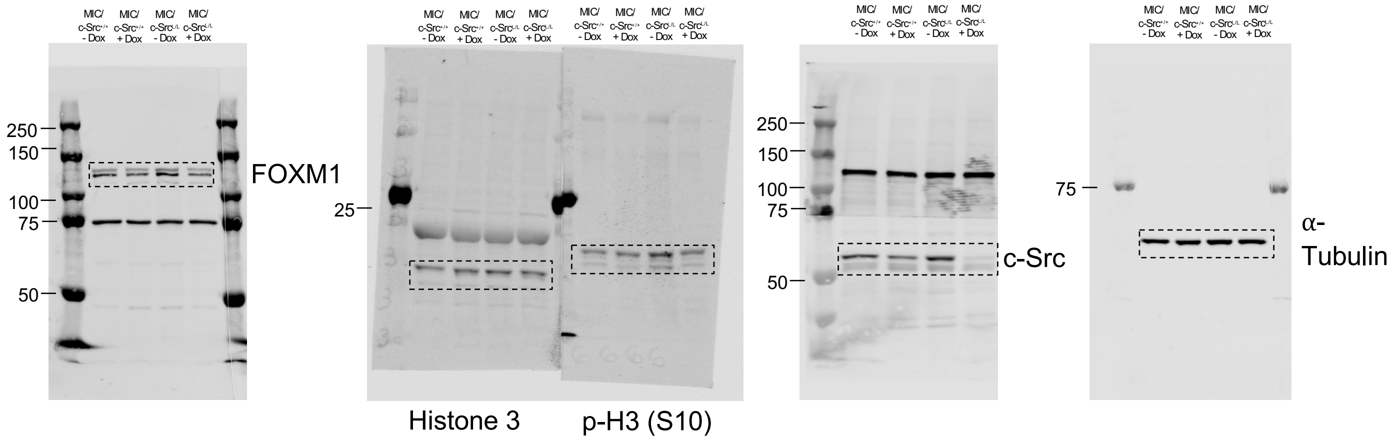

Full unedited gel for Supplemental Figure 4D

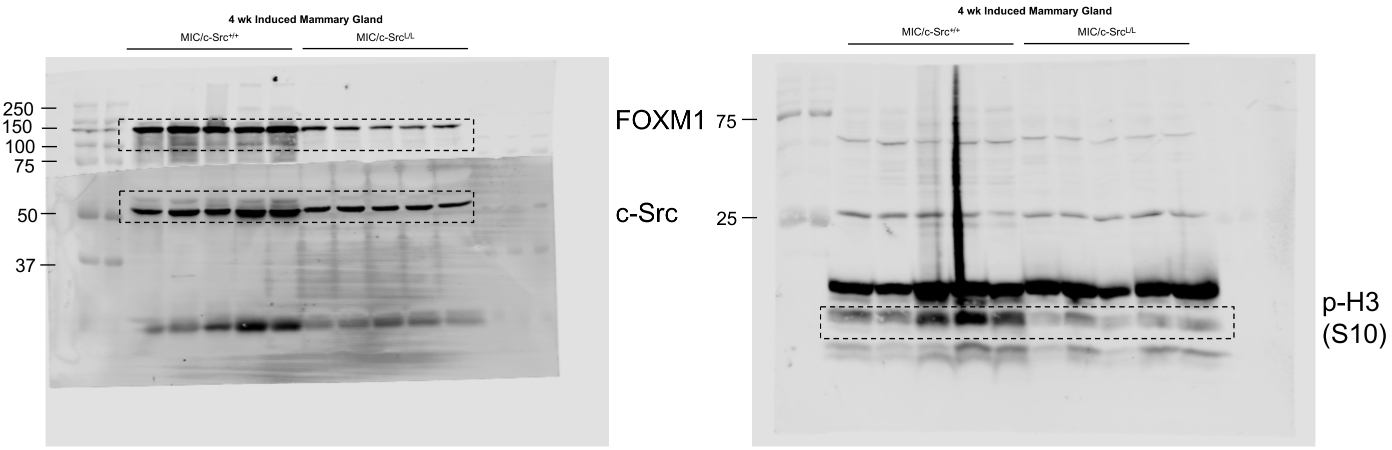

Uncropped western blots for Supplemental Figure 2A (Continued), Supplemental Figure 4C and Supplemental Figure 4D. Black dotted lines indicate regions that were cropped for the figures.

Full unedited gel for Supplemental Figure 4D (Continued)

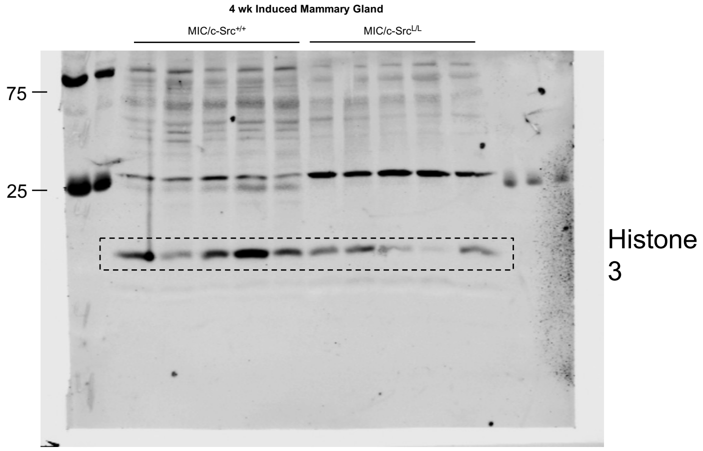

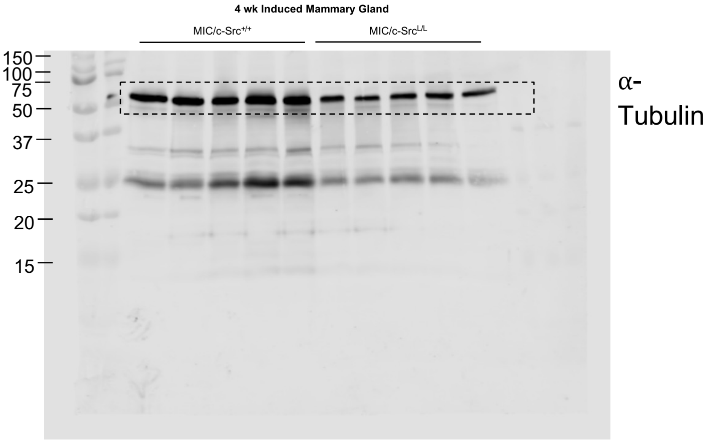

Full unedited gel for Supplemental Figure 5B

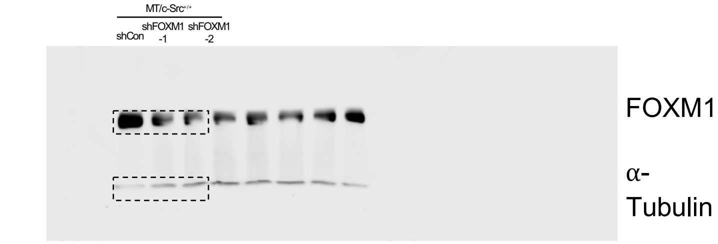

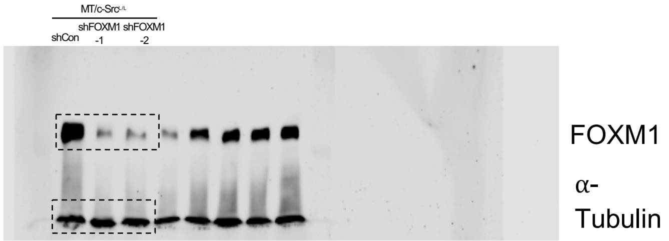

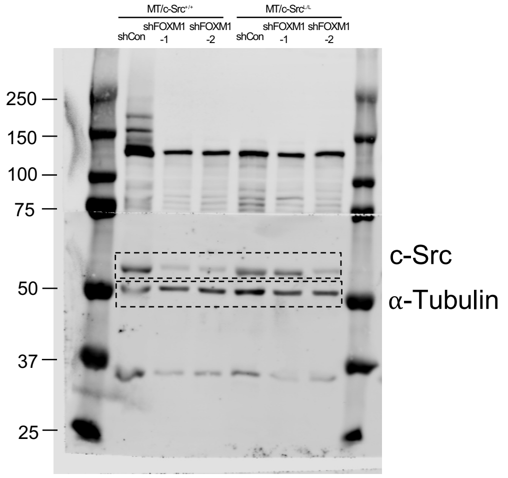

Uncropped western blots for Supplemental Figure 4D (Continued) and Supplemental Figure 5B. Black dotted lines indicate regions that were cropped for the figures.

Full unedited gel for Supplemental Figure 5C

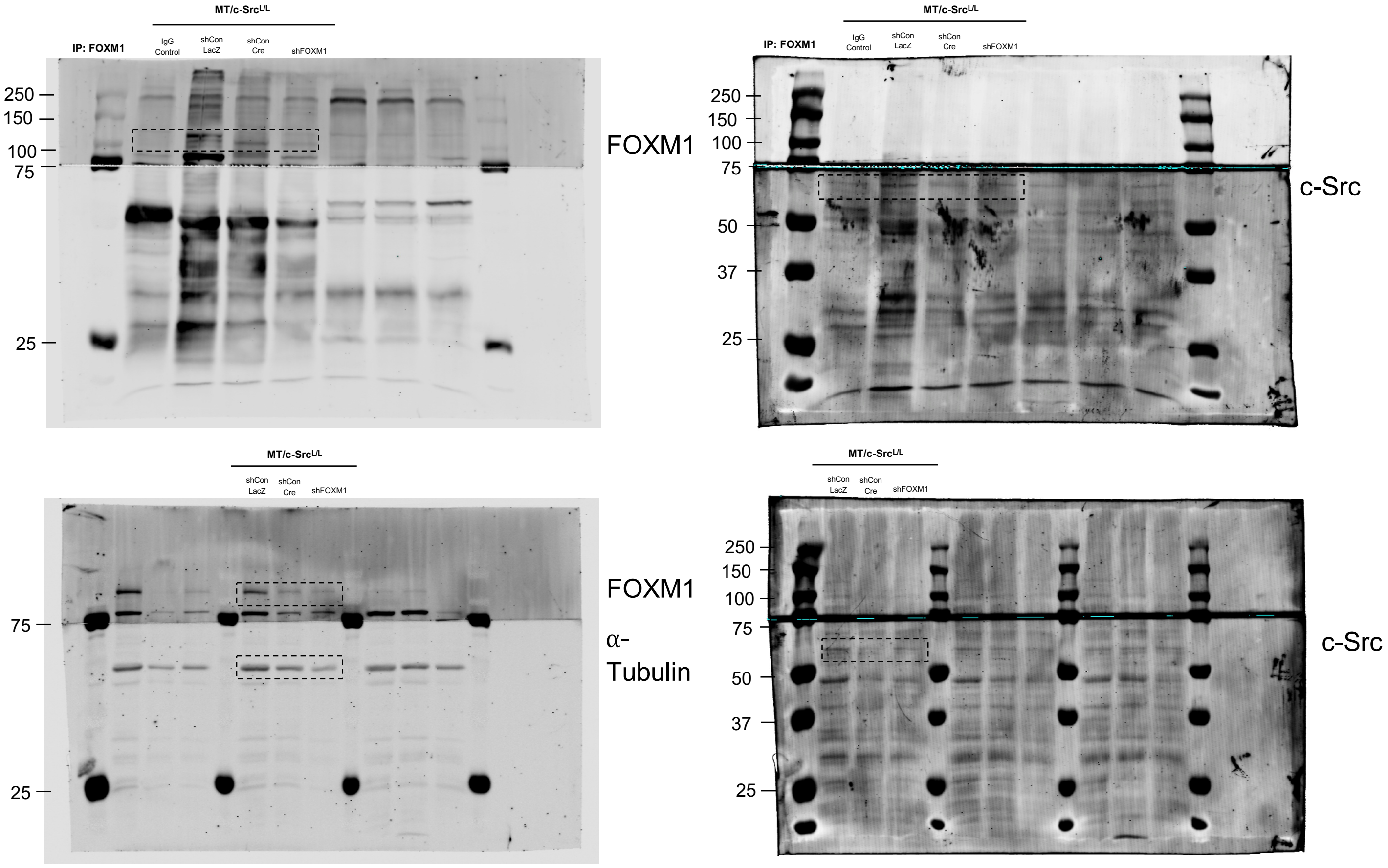

Full unedited gel for Supplemental Figure 5D

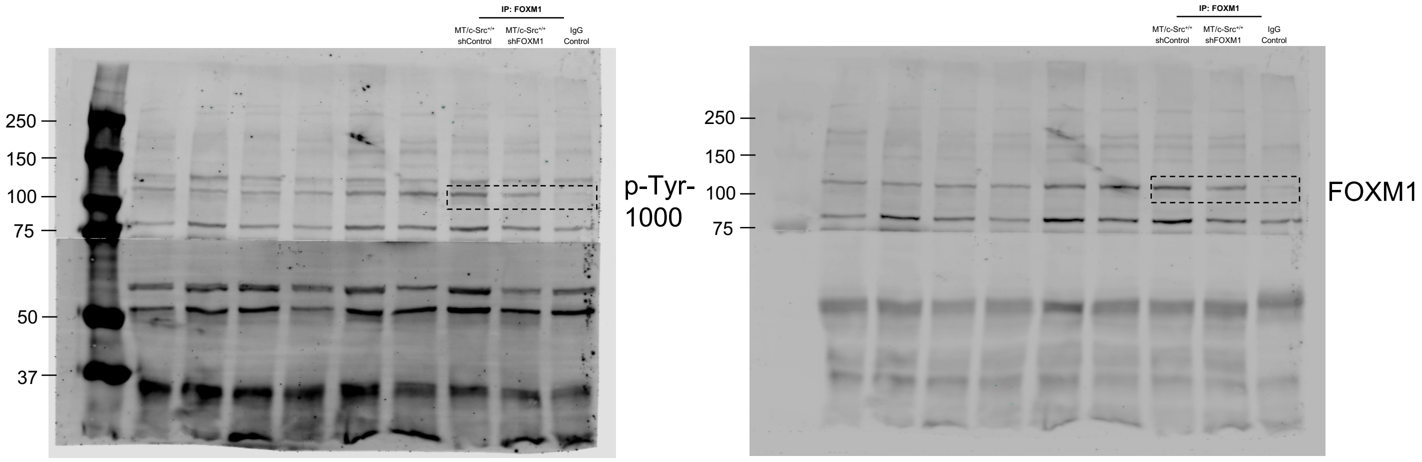

Uncropped western blots for Supplemental Figure 5C and Supplemental Figure 5D. Black dotted lines indicate regions that were cropped for the figures.

Full unedited gel for Supplemental Figure 5E

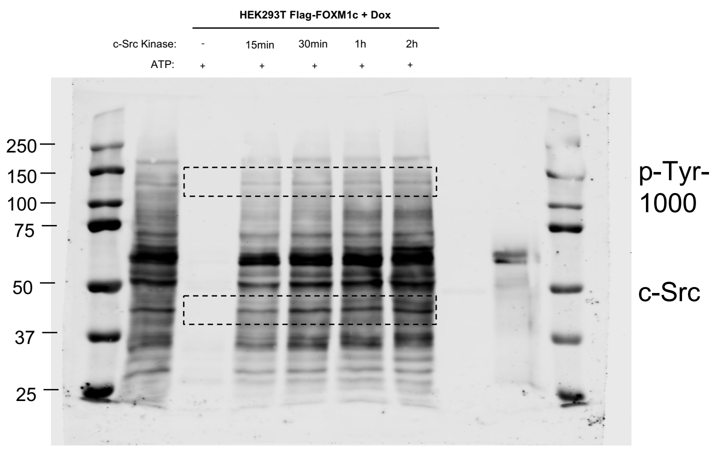

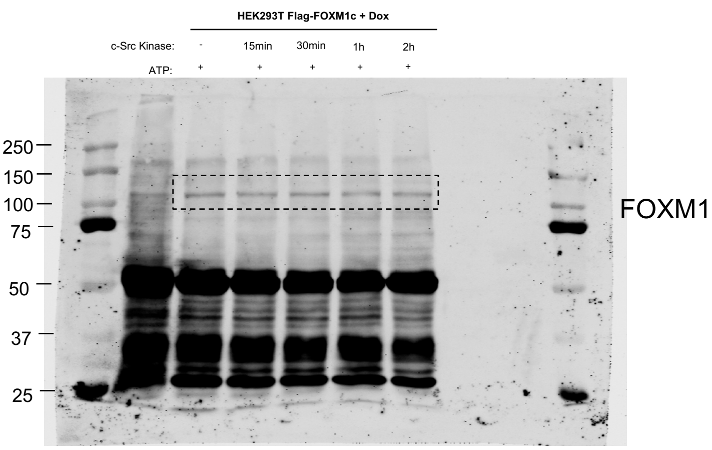

Uncropped western blots for Supplemental Figure 5E. Black dotted lines indicate regions that were cropped for the figures.

Full unedited gel for Supplemental Figure 6A

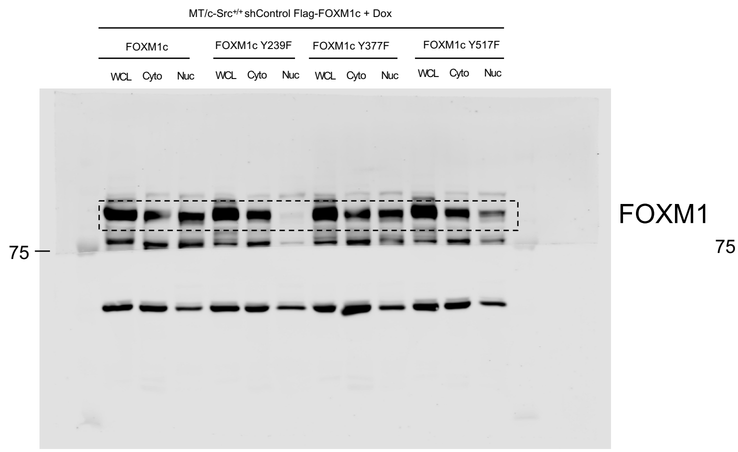

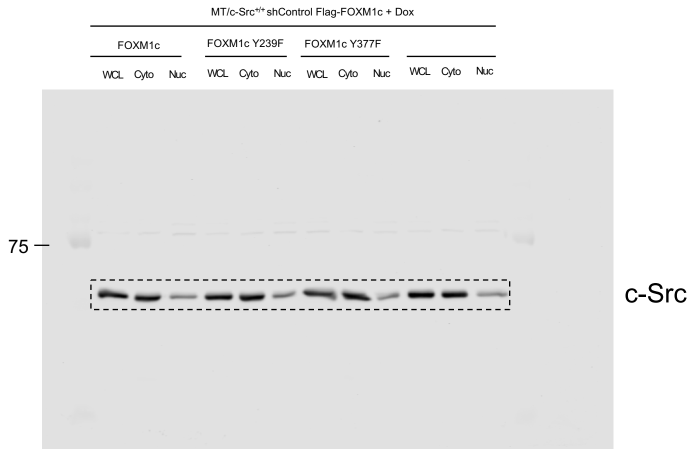

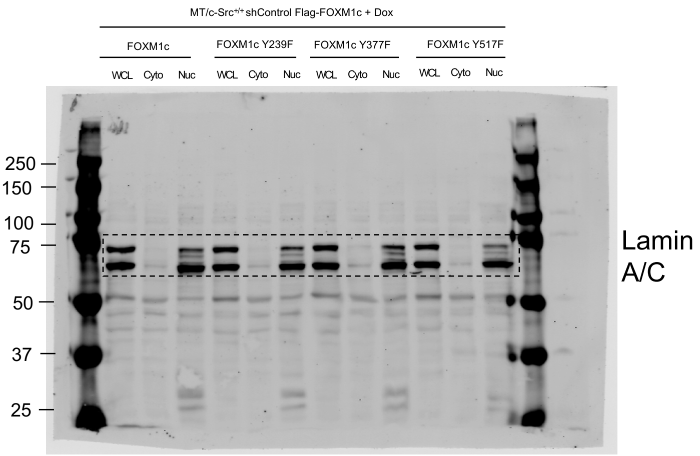

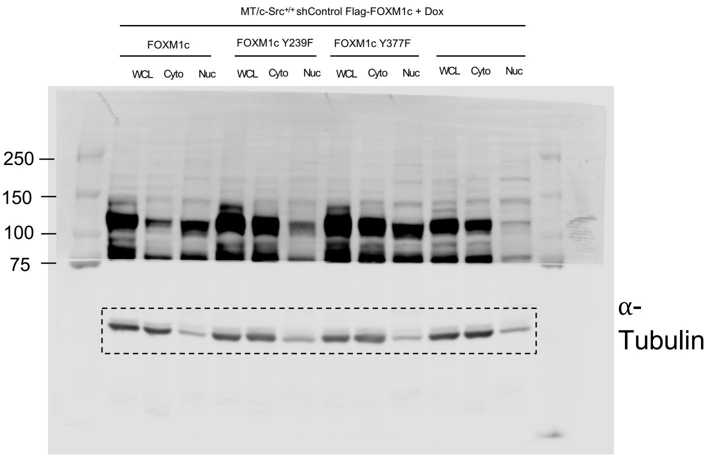

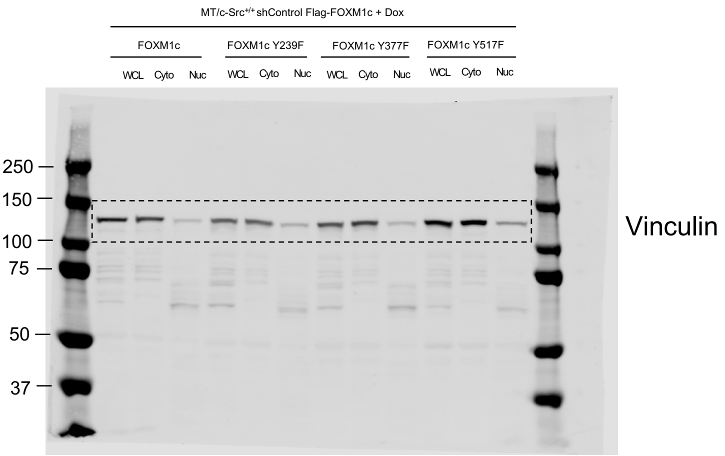

Uncropped western blots for Supplemental Figure 6A. Black dotted lines indicate regions that were cropped for the figures.

Full unedited gel for Supplemental Figure 7B

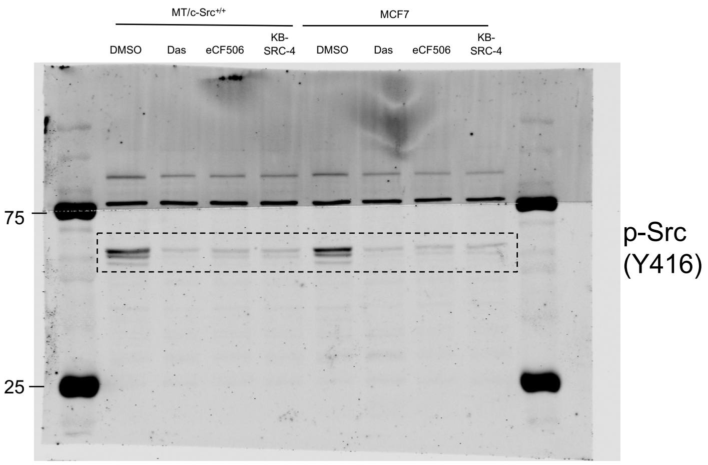

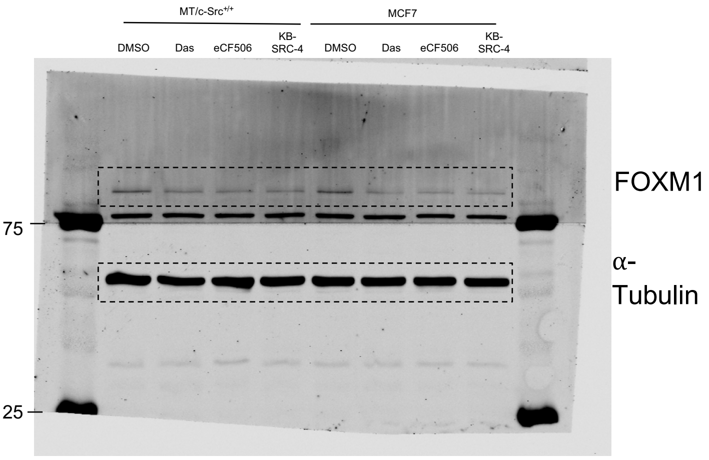

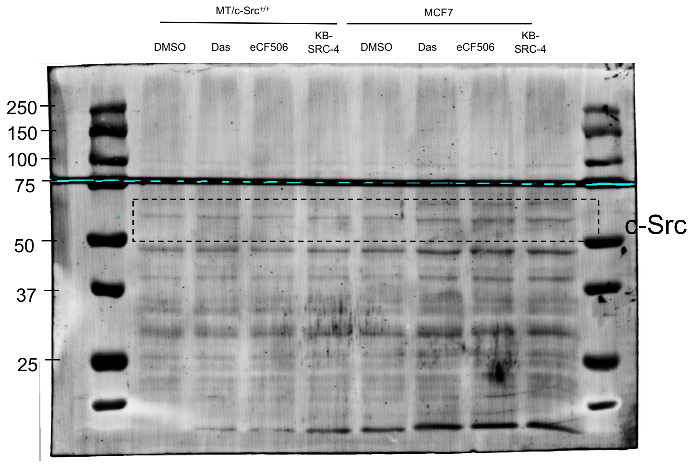

Uncropped western blots for Supplemental Figure 7B. Black dotted lines indicate regions that were cropped for the figures.

Full unedited gel for Supplemental Figure 11C

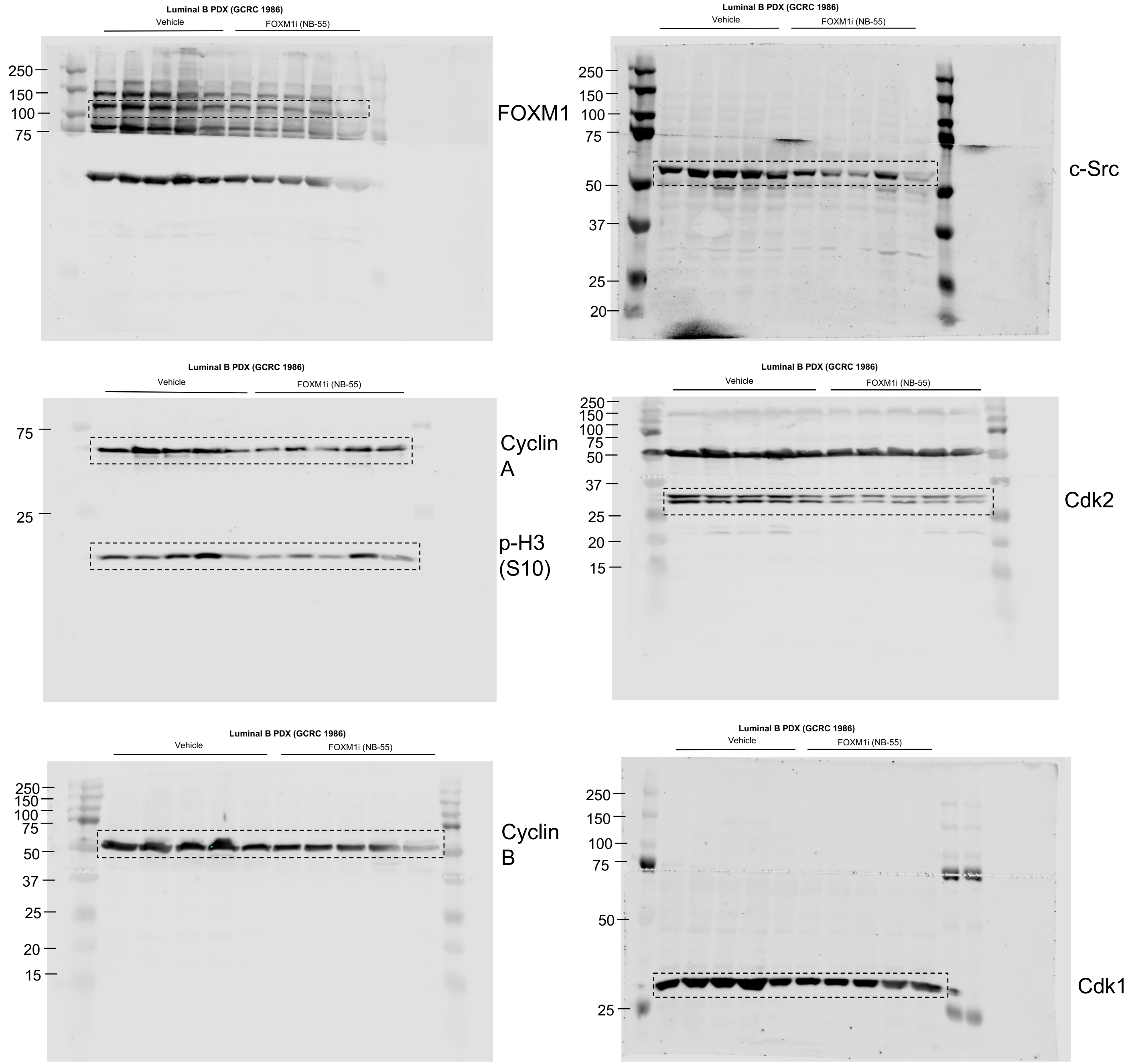

Uncropped western blots for Supplemental Figure 11C. Black dotted lines indicate regions that were cropped for the figures.

Full unedited gel for Supplemental Figure 11C (Continued)

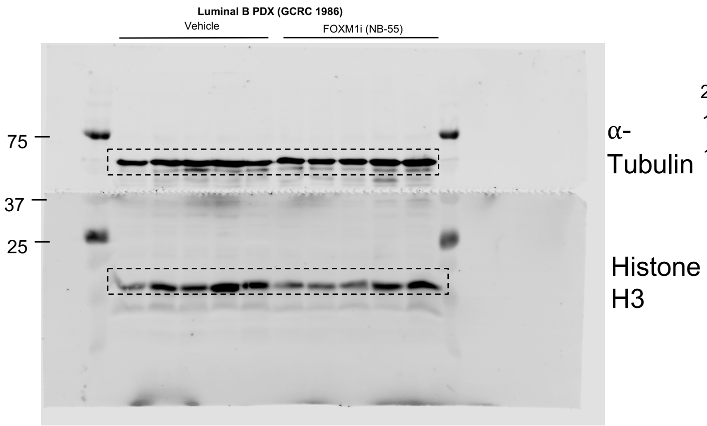

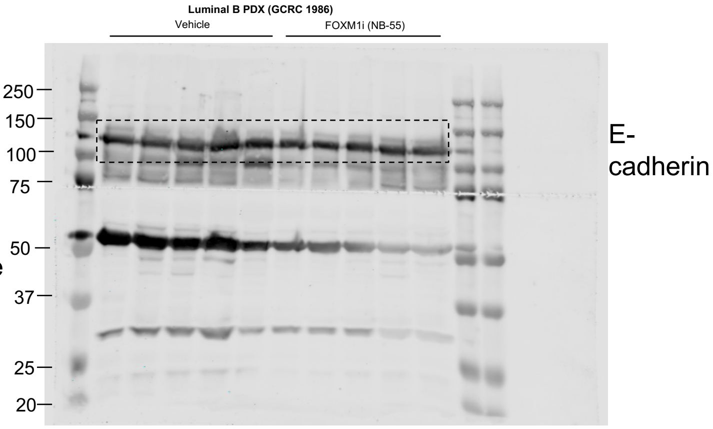

Uncropped western blots for Supplemental Figure 11C (Continued). Black dotted lines indicate regions that were cropped for the figures.
